# Bipyrrole boomerangs via Pd-mediated tandem cyclization–oxygenation. Controlling reaction selectivity and electronic properties

**DOI:** 10.3762/bjoc.16.81

**Published:** 2020-05-04

**Authors:** Liliia Moshniaha, Marika Żyła-Karwowska, Joanna Cybińska, Piotr J Chmielewski, Ludovic Favereau, Marcin Stępień

**Affiliations:** 1 Wydział Chemii, Uniwersytet Wrocławski, ul. F. Joliot-Curie 14, 50-383 Wrocław, Poland; 2 PORT – Polski Ośrodek Rozwoju Technologii, ul. Stabłowicka 147, 54-066 Wrocław, Poland; 3 Université Rennes, CNRS, ISCR (Institut des Sciences Chimiques de Rennes) UMR 6226, F-35000 Rennes, France

**Keywords:** donor–acceptor systems, double C–H bond activation, helicenes, pyrroles

## Abstract

Boomerang-shaped bipyrroles containing donor–acceptor units were obtained through a tandem palladium-mediated reaction consisting of a cyclization step, involving double C–H bond activation, and a double α-oxygenation. The latter reaction can be partly suppressed for the least reactive systems, providing access to α-unsubstituted boomerangs for the first time. These “α-free” systems are highly efficient fluorophores, with emission quantum yields exceeding 80% in toluene. Preliminary measurements show that helicene-like boomerangs may be usable as circularly polarized luminescent materials.

## Introduction

Nanographenes and other polycyclic aromatics as well as their heterocyclic analogues are typically obtained by tandem cyclodehydrogenations of oligoaryl precursors [1–3]. This general strategy is attractive because it does not require prefunctionalization of coupling sites and because it provides rapid access to complex π systems. Such cyclodehydrogenations can be performed using diverse oxidants [4], with FeCl_3_ being particularly notable for its versatility, ease of use, and low price [5]. Nevertheless, the synthetic utility of oxidative couplings is often limited by several factors [6]. Consequently, incomplete ring fusion and various side reactions, e.g., chlorination [7], or unexpected rearrangements, are frequently observed [8]. The use of oxidative couplings is further limited for the synthesis of strained [9–10], electron-deficient, or sterically congested aromatics [11–14]. Some of these limitations can be overcome by using prefunctionalized precursors [11], as exemplified by reductive Ullmann-type chemistry [15], catalytic direct arylations [11,14,16–17], photoinduced cyclodehydrochlorinations [18–19], or nucleophilic oxidative couplings [20], which may offer milder reaction conditions and superior regioselectivities, at the expense of atom and step economy [21]. The latter disadvantage may be obviated by transition-metal-mediated double C–H bond activation [22–23], which is functionally equivalent to conventional oxidative coupling reactions, and has become a powerful synthetic tool with a rapidly growing scope of use [24–26]. However, in the field of π-conjugated materials, this strategy has so far remained relatively unexplored [27].

**Scheme 1:**
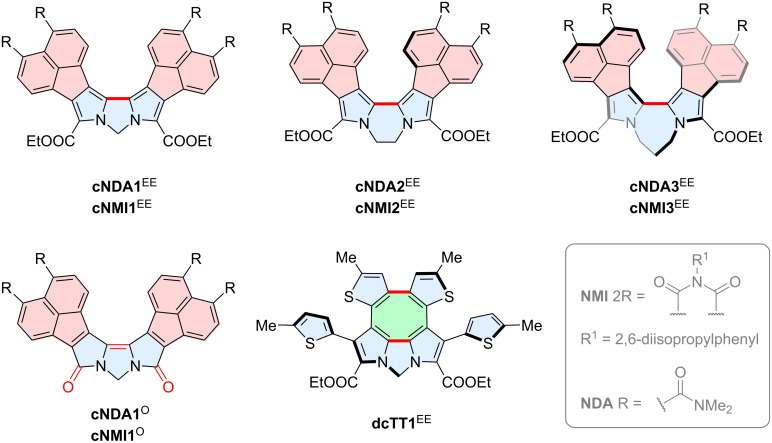
The previously reported family of the boomerang bipyrroles obtained by Pd-induced double C–H bond activation [32].

As a part of our ongoing research on π-extended electron-deficient oligopyrroles [13,28–31], we have recently reported that Pd(II)-mediated double C–H activation can be a useful tool for conversion of 1,*n*-dipyrrolylalkanes into boomerang-shaped *N,N'*-bridged α,α'-bipyrroles that are not accessible by means of conventional oxidative coupling methods (Scheme 1) [32]. Our approach is applicable to electron-deficient and sterically encumbered systems, notably those based on pyrrole derivatives fused with naphthalenediamide (NDA) and naphthalenemonoimide (NMI) moieties. The double C–H bond activation initially used palladium(II) acetate in acetic acid as the coupling system. The subsequent screening revealed, however, that a catalytic coupling could be also achieved in the presence of silver(I) carbonate as the stoichiometric oxidant. The scope of such Pd(II)-induced couplings was further developed into tandem processes involving consecutive cyclization of substituents (**dcTT**^EE^) and oxygenation of pyrrolic α-positions to form lactams **cNDA1**^O^ and **cNMI1**^O^. The mechanism of those transformations was subsequently explored using NMR spectroscopy and DFT calculations [33]. In particular, the unprecedented double α-oxygenation of bipyrroles was shown to occur through stepwise acetoxylation, which we found to compete with α–α oligomerization. These new bipyrrole boomerangs exhibited enhanced fluorescence with Φ_fl_ values of up to 67%, while their bandgaps and chiroptical responses could be tuned by twisting the bipyrrole chromophore. The solvatochromism and apparent superradiance of these chromophores indicated a potential involvement of solvent-induced symmetry-breaking charge transfer in the excited state [34]. Here we report that the above Pd-mediated chemistry is capable of producing highly twisted dilactam boomerangs and provide first examples of α-free boomerang systems. These new derivatives are of interest as emitters for both polarized and unpolarized luminescence.

## Results and Discussion

### Synthesis

**Scheme 2:**
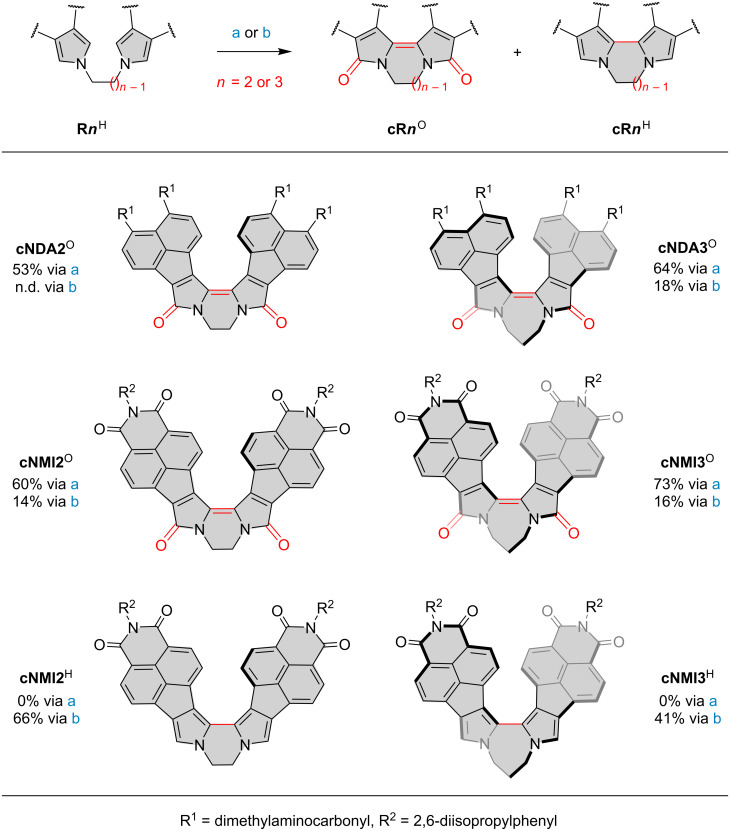
Synthesis and structures of α-free and α-oxygenated bipyrrole boomerangs. Reagents and conditions: (a) 30 mM in AcOH, 3 equiv Pd(OAc)_2_, 6 equiv KOAc, 120 °C, 1 h; (b) 3 mM in AcOH, 3 equiv Pd(OAc)_2_, 120 °C, 1 h. Isolated yields are given for each set of conditions. *M* enantiomers are depicted for **cNDA3**^O^, **cNMI3**^O^, **cNMI3**^H^. n.d. = not determined.

The starting 1,*n*-dipyrrolylalkane precursors (**R*n***^H^, *n* = 2 or 3) were synthesized by reacting the appropriate pyrrolyl anions with either ethylene ditosylate or 1,3-dibromopropane (Scheme S1, Supporting Information File 1). In our initial coupling experiments, 1,2-dipyrrolylethanes (**NDA2**^H^, **NMI2**^H^) and 1,3-dipyrrolylpropanes (**NDA3**^H^, **NMI3**^H^) were reacted with palladium(II) acetate in acetic acid to furnish the expected bipyrrole dilactams in 43–53% yields (Table 1, entries 1, 6, 11 and 17). Remarkably, it was found that **cNMI2**^O^ and **cNMI3**^O^ were obtained along with the intermediate α-unsubstituted boomerangs, **cNMI2**^H^ and **cNMI3**^H^, respectively (Scheme 2). Analogous α-unsubstituted intermediates (**cNDA2**^H^ and **cNDA3**^H^) were not isolated in reactions involving **NDA2**^H^ and **NDA3**^H^. The latter behavior is consistent with the selectivity pattern observed previously for α-unsubstituted dipyrrolylmethanes **NDA1**^H^ and **NMI1**^H^, for which we were only able to isolate the corresponding dilactams **cNDA1**^O^ and **cNMI1**^O^, respectively [32]. Transient formation of **cNDA1**^H^ could, however, be observed in situ for reactions involving **NDA1**^H^ [33].

Subsequent screening revealed that the yield of **cNMI2**^H^ and **cNMI3**^H^ could be increased when reactions were performed in more dilute solutions (Table 1, entries 14 and 20). Following our previous experimental and computational findings [32–33], we also checked whether the yields of dilactam products might be improved by increasing the concentration of acetate anions in the reaction mixture. Indeed, annulations of **NDA*n***^H^ and **NMI*n***^H^ carried out in the presence of 6 equiv of potassium acetate produced the corresponding dilactams in higher yields (53–73%) with complete conversion (Table 1, entries 2, 7, 12, and 18). Under these conditions, the **cNMI2**^H^ and **cNMI3**^H^ intermediates were not isolated. When the same reactions were, however, performed in higher dilution, the yields decreased and the **cNMI*n***^O^ to **cNMI*n***^H^ ratio was almost 1:1, showing the important role of the dipyrrolylalkane concentration in these transformations (Table 1, entries 15 and 21). Additional experiments carried out on **R*n***^H^ precursors revealed that the coupling and α-oxygenation can also be achieved with 1 equiv of Pd(OAc)_2_ in the presence of 2 equiv of silver carbonate. Nevertheless, Ag_2_CO_3_ oxidation provided lower yields and the efficiency of this variant was dramatically diminished when the loading of palladium(II) acetate was decreased below 1 equiv (Table 1, entries 16 and 22).

**Table 1:** Screening of reaction conditions.^a^

entry	reactant	*c* [mM]^b^	Pd(OAc)_2_^c^	additive^d^	**cR*n***^O^ [%]^e^	**cR*n***^H^ [%]^e^

1	**NDA2**^H^	30	3	none	46	0
2	**NDA2**^H^	30	3	KOAc	53	0
3	**NDA2**^H^	30	1	Ag_2_CO_3_	34	0
4	**NDA2**^H^	3	3	none	n.d.^f^	0
5	**NDA2**^H^	3	3	KOAc	n.d.^f^	0
6	**NDA3**^H^	30	3	none	43	0
7	**NDA3**^H^	30	3	KOAc	64	0
8	**NDA3**^H^	30	1	Ag_2_CO_3_	37	0
9	**NDA3**^H^	3	3	none	18	0
10	**NDA3**^H^	3	3	KOAc	23	0
11	**NMI2**^H^	30	3	none	52	6
12	**NMI2**^H^	30	3	KOAc	60	0
13	**NMI2**^H^	30	1	Ag_2_CO_3_	43	14
14	**NMI2**^H^	3	3	none	14	66
15	**NMI2**^H^	3	3	KOAc	32	30
16	**NMI2**^H^	3	0.1	Ag_2_CO_3_	<10%	<10%
17	**NMI3**^H^	30	3	none	56	9
18	**NMI3**^H^	30	3	KOAc	73	0
19	**NMI3**^H^	30	1	Ag_2_CO_3_	34	6
20	**NMI3**^H^	3	3	none	16	41
21	**NMI3**^H^	3	3	KOAc	15	12
22	**NMI3**^H^	3	0.1	Ag_2_CO_3_	19	traces

^a^Conditions: acetic acid, 120 °C, 1 h; ^b^concentration of the starting dipyrrolylalkane (**R*n***^H^); ^c^[equiv] ^d^KOAc (6 equiv), Ag_2_CO_3_ (2 equiv); ^e^isolated yields; ^f^n.d. = not determined. Only traces of **cNDA2**^O^ were present in the crude mixture.

### Structure

The identity of the α-oxygenated products, **cR*n***^O^, was determined on the basis of high-resolution mass spectrometry and ^1^H and ^13^C NMR data. In particular, the ^1^H NMR spectrum of **cNMI2**^O^ revealed the absence of the pyrrolic α-H resonances, whereas the endocyclic CH_2_ moiety yielded a pair of very broad peaks at ca. 4.8–3.4 ppm. This splitting, which was also observed for **cNDA2**^O^, is consistent with slow inversion of helicity occurring at the ethylene bridge. In the ^1^H NMR spectrum of **cNMI2**^H^, the linker CH_2_ moiety and the 2,6-diisopropylphenyl (dipp) CH unit each produced a single broadened signal, indicating that the helix inversion occurs in the fast exchange regime. This apparently faster inversion in **cNMI2**^H^ than in the **cNMI2**^O^ and **cNDA2**^O^ lactams correlates with the higher bond order of the α–α linkage in the latter two systems. The chirality of **cNMI3**^H^ is reflected in its ^1^H NMR spectrum, which shows diastereotopic differentiation of N–CH_2_ protons of the bridge and the CH signals of the dipp substituents, consistent with a rigid *C*_2_-symmetric structure. Analogous diastereotopic effects are observed for **cNDA3**^O^ and **cNMI3**^O^. For **cNDA3**^O^, the ^1^H NMR spectrum is additionally complicated by the partially restricted rotation of the *N*,*N*-dimethylamide substituents in the NDA units. In conjunction with the helicity of the ring system, this restriction leads to effective diastereomerism.

**Figure 1:**
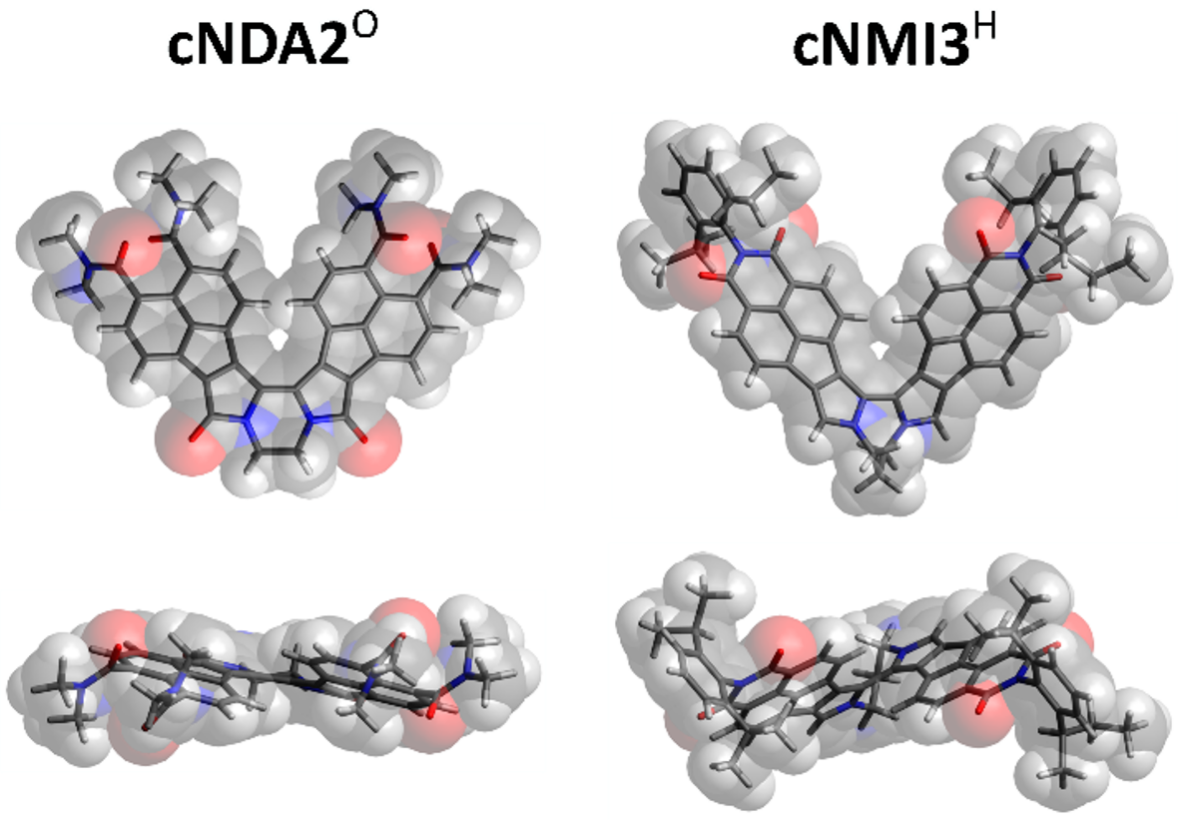
DFT-Optimized structures (B3LYP/6-31G(d,p)) of **cNDA2**^O^ and **cNMI3**^H^.

The three-dimensional structures of all boomerangs were modeled using DFT calculations (Figure 1 and Supporting Information File 1). The length of the linker (*n*) in **cNDA*n***^X^ and **cNMI*n***^X^ controls the in- and out-of-plane geometry of the chromophore. The observed changes can be expressed in terms of two parameters: *α*, the angle between the monopyrrole axis and the N–N vector, and θ, the torsion angle between the two monopyrrole axes (Supporting Information File 1, Table S1). In contrast to the previously reported **cR1**^O^ dilactams, which were nearly planar [32], systems with *n* = 2 and 3 are characterized by a twisting distortion, which produces helicene-like conformations. The distortion results from an increased splay angle α between the two pyrrolic subunits, which leads to a greater steric congestion and, consequently, to an increase of the θ twist.

### Electronic properties

**Figure 2:**
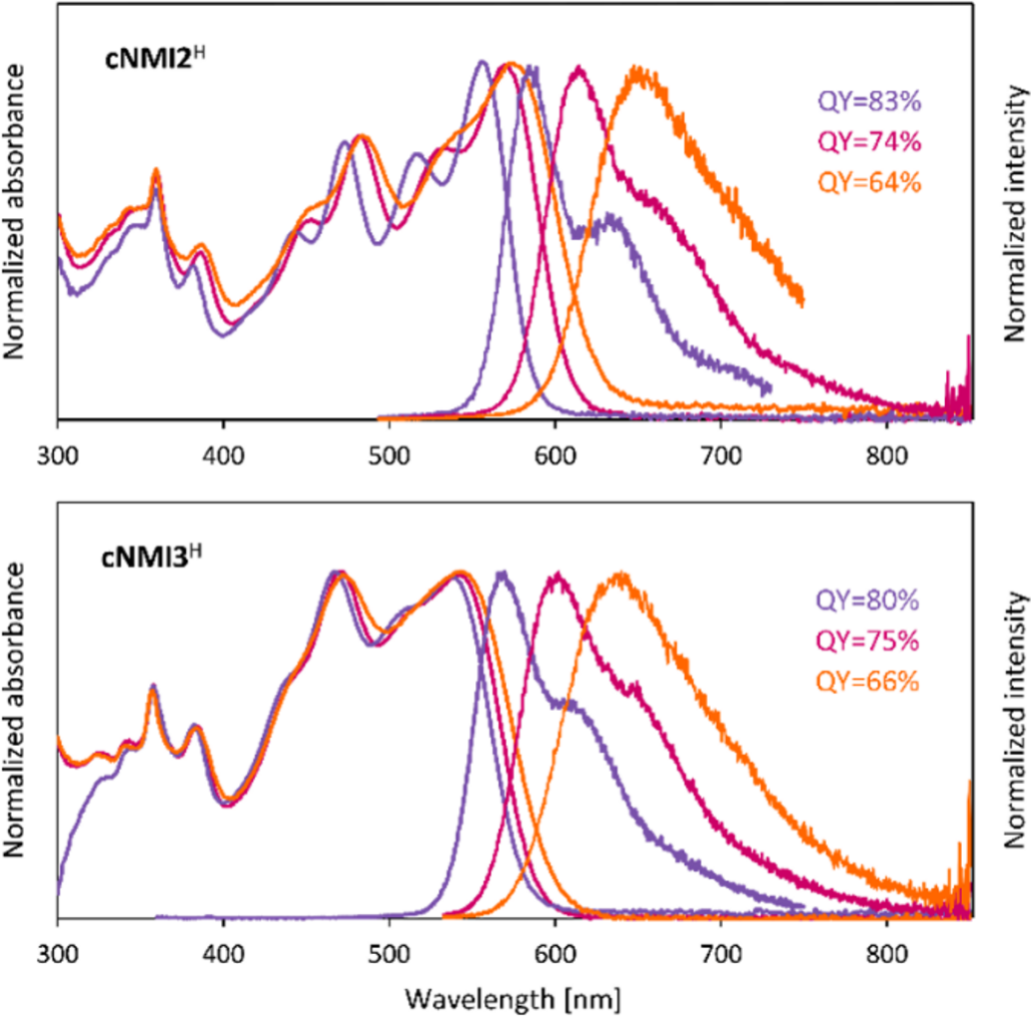
Absorption and emission spectra of **cNMI2**^H^ (top) and **cNMI3**^H^ (bottom) measured in toluene, dichloromethane and acetonitrile.

The absorption spectra of the **cNMI2**^O^ and **cNMI3**^O^ bipyrroles, recorded in dichloromethane, are red-shifted by respectively 74 and 86 nm relative to their **cNDA*n***^O^ congeners (Table 2, Figure 2, see also Supporting Information File 1 for more optical data). Within each lactam series, when the bridge length *n* is increased from 2 to 3, the lowest-energy band is shifted by ca. 17 nm to longer wavelengths. Pairs of lactam rings present in **cNMI2**^O^ and **cNMI3**^O^ form a quinoidal substructure, which produces a very significant bathochromic shift of their lowest-energy absorption bands (up to 176 nm in toluene) in comparison to the of α-unsubstituted analogues. Lactam bipyrroles **cNDA*n***^O^ and **cNMI*n***^O^ show noticeable solvatochromism which is stronger for *n* = 2 and is always negative. In contrast, the solvatochromism of α-free boomerangs is positive and even more pronounced. On going from toluene to acetonitrile, the onset of the lowest-energy band of **cNMI2**^H^ is shifted to longer wavelengths by 25 nm.

The **cNMI2**^H^ and **cNMI3**^H^ bipyrroles are very efficient fluorophores (Table 2), noticeably more emissive than the lactam analogues and the other previously reported boomerangs [32]. Highest fluorescence quantum yields were observed in toluene (83 and 80%, respectively). For comparison, the Φ_fl_ values for the lactams **cNMI2**^O^ and **cNMI3**^O^ are about 1%. Interestingly, the fluorescence of **cNMI*n***^H^ boomerang bipyrroles showed a stronger solvatochromic dependence than observed in their absorption spectra (Figure 2). In the more polar solvents, the emission profiles became significantly red shifted and broadened. At the same time, the fluorescence was only moderately quenched with increasing solvent polarity, and the quantum yields of 64 and 66% were recorded in acetonitrile for **cNMI2**^H^ and **cNMI3**^H^, respectively.

To explore the chiral properties of the helically distorted boomerangs, the separation of enantiomers was attempted for all of them by means of chiral HPLC with CD detection, leading to enantioenriched samples of some boomerangs. The enantiomers of **cR2**^O^ were found to racemize very fast, thus it was not possible to separate them. The **cR3**^X^ systems, which contain a 7-membered ring, showed a remarkably diverse behavior. Enantiomers of **cNMI3**^O^ could not be separated, presumably because of very rapid racemization. The half-life time of **cNDA3**^O^ enantiomers (ca. 0.54 min) was too short to record their CD spectra, but was long enough for a kinetics study (Supporting Information File 1, Figures S8 and S9). Enantioenriched samples of **cNMI3**^H^ were configurationally most stable, showing no loss of their optical activity over the course of several hours in solution. Their CD spectra could be satisfactorily correlated with the TD-DFT data obtained for the **cNMI3**^H^ enantiomers (Supporting Information File 1, Figures S5 and S22). Circularly polarized luminescence (CPL) measurements performed for enantioenriched samples of **cNMI3**^H^ reveled weak signals of opposite signs, with a maximum at ca. 570 nm consistent with the unpolarized luminescence of this system (Supporting Information File 1, Figure S6). The CPL signals rapidly decayed during the measurements, without any significant loss of the unpolarized emission intensity. This behavior, which precluded a quantitative analysis of the CPL properties, may be attributed to a photoinduced racemization process. The differences in configurational stability of boomerangs are reproduced by DFT calculations, which predict inversion barriers Δ*G*^‡,298^ of 20.0 and 24.7 kcal/mol for models of **cNDA3**^O^ and **cNMI3**^H^, respectively (Supporting Information File 1, Figures S31 and S32). The latter value is consistent with the observed greatest stability of **cNMI3**^H^.

Frontier molecular orbitals of the boomerangs reveal features characteristic of donor–acceptor systems (Supporting Information File 1, Figures S25–S30). For dilactams, **cR*n***^O^, the HOMO orbital is primarily localized on the dilactam (bipyrrole) moiety, however, with some non-zero amplitudes on the NDA/NMI fragment, whereas the LUMO level encompasses the π system more evenly. In the **cNMI*n***^H^ series (*n* = 2, 3), the HOMO and LUMO are formed by superposition of the corresponding MOs of the monomeric NMI pyrrole (Supporting Information File 1, Figures S23 and S24). However, the LUMO has observably lower amplitudes on the bipyrrole part of the molecule, whereas a more uniform coverage of the π system is seen for the HOMO orbital. The experimentally observed bandgap variations and absorption profiles (Table 2 and Table 3, Supporting Information File 1, Figures S1–S4, and S11–S16) were qualitatively reproduced in TD-DFT calculations performed for **cNDA2**^O^, **cNDA3**^O^, and **cNMI3**^H^ (Table 2 and Table 3, Supporting Information File 1, Tables S2–S4).

**Table 2:** Experimental and calculated^a^ properties of fused bipyrroles.

species	MO energies [eV]^a^			λ_max_^abs^ [nm]			λ_max_^em^ [nm] (QY)		
	HOMO	LUMO	HLG^b^	tol^c^	DCM^d^	MeCN^e^	tol^c^	DCM^d^	MeCN^e^

**cNDA2**^O^	−5.47	−3.26	2.21	620	610	605	655 (0.07)	–	–
**cNDA3**^O^	−5.34	−3.28	2.06	636	620	617	694 (0.25)	–	–
**cNMI2**^O^	−5.95	−4.03	1.92	693	684	676	738 (0.01)	–	–
**cNMI3**^O^	−5.88	−4.03	1.85	711	706	703	768 (0.01)	–	–
**cNMI2**^H^	−5.50	−2.78	2.72	555	570	580	575 (0.83)	74 (0.61)	650 (0.64)
**cNMI3**^H^	−5.56	−2.73	2.83	535	540	546	574 (0.80)	605 (0.75)	655 (0.66)

^a^Molecular orbital energies, B3LYP/6-31G(d,p); ^b^HOMO–LUMO gap; ^c^in toluene; ^d^in dichloromethane; ^e^in acetonitrile.

The redox properties of the new bipyrrole boomerangs were investigated by means of cyclic (CV) and differential pulse (DPV) voltammetry (Supporting Information File 1, Figures S11–S16). All systems showed at least two reversible one-electron reduction couples and up to two oxidation couples. The first oxidation was reversible for all systems studied except **cNMI2**^H^ and **cNDA3**^O^. The second oxidations were chemically irreversible in all cases and typically produced new irreversible peaks upon the consecutive cathodic scans. In the previously reported **cNMI*n***^EE^ series (*n* = 1, 2, 3) it was also possible to observe one reversible oxidation and four reductions, the first two being reversible [32]. The first oxidation potentials (*E*_Ox1_) vary from 0.62 to 1.09 V, while the first reduction potentials (*E*_Red1_) range from −1.54 to −0.60 V (Table 3). The boomerangs with unsubstituted pyrrole α-positions (**cNMI3**^H^ and **cNMI2**^H^) are characterized by significantly lower oxidation and reduction potentials than the corresponding dilactam analogues. The electrochemical data obtained for **cNMI3**^O^ and **cNMI2**^O^ indicate a destabilization of their HOMOs by about 0.5 eV while relative stabilization of the LUMO approaches 1 eV with respect to **cNMI3**^H^ and **cNMI2**^H^. This comparably stronger LUMO stabilization in the NMI lactam systems results in a considerable decrease of their electrochemical gap (Δ*E*, by ca. 0.5–0.7 V), relative to the α-free analogues. Δ*E* values for lactams **cR*n***^O^ are relatively insensitive to the length of the alkylene linker *n*, in spite of the large changes of the inter-subunit torsion (θ, Supporting Information File 1, Table S1) caused by the increase of *n*. In line with the absorption spectroscopy data, the Δ*E* gap is reduced in the **cNMI*n***^O^ series by 0.25 to 0.36 V relative to the corresponding **cNDA*n***^O^ analogues. Interestingly, the difference between the first and second reduction potentials in the **cR*n***^O^ dilactams is in the range of 0.25 to 0.33 V, indicating a strong coupling between the subunits. In comparison, this potential difference is much smaller in the α-free analogues **cNMI3**^H^ and **cNMI2**^H^ (ca. 0.1 V).

**Table 3:** Electrochemical data for the **cNDA*n***^O^,**cNMI*n***^O^ and **cNMI*n***^H^ boomerangs derived from differential pulse voltammograms.^a^

species	*E*_Red4_	*E*_Red3_	*E*_Red2_	*E*_Red1_	*E*_Ox1_	*E*_Ox2_	Δ*E*^b^

**cNDA1**^O c^	−2.52^d^	−1.95^d^	−1.36	−1.03	0.88^d^	–	1.91
**cNDA2**^O^	–	−1.82^d^	−1.36	−1.07	0.98	1.08^d^	2.05
**cNDA3**^O^	–	–	−1.31	−1.03	0.89^d^	1.01^d^	1.92
**cNMI1**^O c^	−1.84	−1.59	−0.83	−0.57	1.09^d^	1.19^d^	1.66
**cNMI2**^O^	−1.90	−1.65	−0.85	−0.60	1.09	–	1.69
**cNMI3**^O^	−1.97	−1.73	−0.86	−0.61	1.04	1.16^d^	1.65
**cNMI2**^H^	–	−2.46^d^	−1.67	−1.59	0.62^d^	0.76^d^	2.21
**cNMI3**^H^	–	−2.54	−1.80	−1.70	0.68	0.98^d^	2.38

^a^Measurements were performed in dichloromethane solution using glassy carbon, platinum rod, and Ag/AgCl as working, auxiliary, and pseudoreference electrodes, respectively. All electrode potentials are in volt and are referenced with the ferrocene/ferrocenium couple as the internal standard. ^b^Electrochemical HOMO–LUMO gap Δ*E* = *E*_Ox1_ – *E*_Red1_. ^c^Previously reported data [32]. ^d^Irreversible.

## Conclusion

The present work shows that the tandem cyclization–oxygenation reaction is a general strategy for the synthesis of low-bandgap bipyrrole boomerangs and is applicable to targets with variable donor–acceptor character and increasing curvature of the bipyrrole linkage. The efficiency of the oxygenation step is dependent on a several of factors, i.e., Pd loading, concentration, and additives. The isolation of unoxygenated products **cNMI2**^H^ and **cNMI3**^H^ emphasizes the role of acceptor units and decreased inter-pyrrole coupling in moderating the reactivity of α positions toward oxygenation. The latter systems are of interest because of their very high fluorescence quantum yields, the best so far recorded for this family of fluorophores. Our preliminary results indicate that bipyrrole boomerangs may be usable as CPL emitters, provided that their helicene-like twist is further stabilized against racemization. Efforts to achieve this goal are currently ongoing in our laboratory.

## Supporting Information

File 1 Synthetic, spectroscopic, and computational details.

File 2 Cartesian coordinates.
