# Supplementary material for: Bipyrrole boomerangs via Pd-mediated tandem cyclization–oxygenation. Controlling reaction selectivity and electronic properties
Source: Beilstein J Org Chem. 2020 May 4;16:895–903. doi: 10.3762/bjoc.16.81 (PMC7214875; doi:10.3762/bjoc.16.81)
Supplement: File 1 — Synthetic, spectroscopic, and computational details. [file Beilstein_J_Org_Chem-16-895-s001.pdf]

BEILSTEIN JOURNAL OF ORGANIC CHEMISTRY

Supporting Information

for

Bipyrrole boomerangs via Pd-mediated tandem cyclization–oxygenation. Controlling reaction selectivity and electronic properties

Liliia Moshniaha, Marika Żyła-Karwowska, Joanna Cybińska, Piotr J. Chmielewski, Ludovic Favereau and Marcin Stępień

Beilstein J. Org. Chem. **2020,** *16,* 895–903. doi:10.3762/bjoc.16.81

Synthetic, spectroscopic, and computational details

License and Terms: This is a supporting information file under the terms of the Creative Commons Attribution License (http://creativecommons.org/licenses/by/4.0). Please note that the reuse, redistribution and reproduction in particular requires that the authors and source are credited.
The license is subject to the *Beilstein Journal of Organic Chemistry* terms and conditions: (https://www.beilstein-journals.org/bjoc)

Table of contents

Experimental S2

Synthesis S3

Additional figures S9

Additional tables S37

NMR spectra S51

Mass spectra S61

References S67

Experimental

General. Toluene was distilled from calcium hydride and stored over molecular sieves when used for absorption, emission, and quantum yields measurments. *N*,*N*-Dimethylformamide was dried using a commercial solvent purification system. Dichloromethane was distilled from calcium hydride. All other solvents and reagents were used as received. Pyrroles **S1** and **S2** were prepared as previously reported.[1]

^{1}H NMR spectra were recorded on high-field spectrometers (^{1}H frequency 500.13 or 600.13 MHz), equipped with broadband inverse or conventional gradient probeheads. Spectra were referenced to the residual solvent signal (chloroform-*d*, 7.24 ppm). Two-dimensional NMR spectra were recorded with 2048 data points in the t_2 domain and up to 2048 points in the t_1 domain, with a 1.5 s recovery delay. All 2D spectra were recorded with gradient selection, with the exception of NOESY and ROESY. NOESY mixing time and ROESY spinlock time were 500 ms and 300 ms, respectively. ^{13}C NMR spectra were recorded with ^{1}H broadband decoupling and referenced to solvent signals ($^{13}CDCl_3$, 77.0 ppm).

High resolution mass spectra were recorded using MALDI ionization in the positive mode.

Electrochemical measurements (DCM, 0.1 M $[NBu_4][PF_6]$, 293 K) were performed on an Metrohm Autolab potentiostat/galvanostat using a glassy-carbon working electrode, platinum rod as the auxiliary electrode, Ag/AgCl as a peudoreference electrode. The voltammograms were referenced against the half-wave potential of Fc^{+}/Fc.

Enantiomers were separated by means of HPLC, using a chiral stationary-phase column Chirex 3010, with UV–vis and CD detection performed using a Jasco 1500 spectropolarimeter equipped with a flowcell. The separated fractions were analyzed by the same HPLC setup to establish enantiomeric excesses. The CD spectra were recorded in dichloromethane.

Photoluminescence excitation (PLE) and emission (PL) spectra as well as decay kinetics (DEC) were taken with the FSL980-sm Fluorescence Spectrometer from Edinburgh Instruments Ltd. A 450 W Xenon arc lamp (PL and PLE) and a Super Continuum Fianium laser were used as excitation sources. Emission spectra were corrected for the recording system efficiency and excitation spectra were corrected for the incident light intensity. PLE and PL spectra and QY were measured with using cooled extended red Hamamatsu photomultiplier operating in range 200–1050 nm. Quantum yield measurements were performed by using an Edinburgh Instruments integrating sphere equipped with a small elliptical mirror and a baffle plate for beam steering and shielding against directly detected light. For the measurement, the integrating sphere replaces the standard sample holder inside the sample chamber. Calculations of quantum yields were made using the software provided by Edinburgh Instruments.

Computational methods. Density functional theory (DFT) calculations were performed using Gaussian 16.[3] DFT geometry optimizations were carried out in unconstrained C_1 symmetry, using molecular mechanics or semi-empirical models as starting geometries. DFT geometries were refined to meet standard convergence criteria, and the existence of a local minimum was verified by a normal mode frequency calculation. Geometry optimizations, frequency calculations were performed using the hybrid functionals B3LYP and the 6-31G(d,p) basis set.[4–7] For all boomerangs 200 electronic transitions were calculated by means of time-dependent DFT (TD-DFT)[8], using the hybrid functional B3LYP and the 6-31G(d,p). The same approach was also used for calculation of HOMO–LUMO energies.

Synthesis

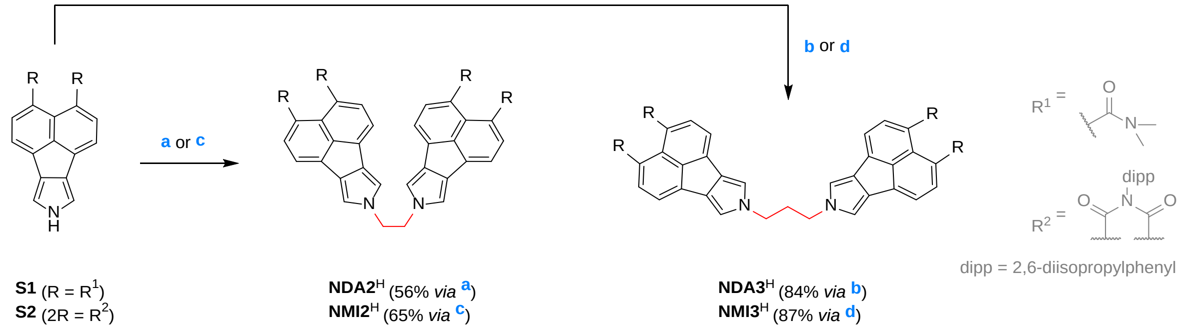

Scheme S1. Synthesis of dipyrrolylalkanes. Reagents and conditions: **(a)** NaH (2.0 equiv), ethylene di(*p*-toluenesulfonate) (0.60 equiv), *N*,*N*-dimethylformamide, 60 °C, overnight;**,** **(b)** NaH (2.0 equiv), 1,3-dibromopropan (0.50 equiv), *N*,*N*-dimethylformamide, 60 °C, overnight; **(c)** Cs_2CO_3 (1.0 equiv), ethylene di(*p*-toluenesulfonate) (0.60 equiv), *N*,*N*-dimethylformamide, 60 °C, overnight;**,** **(d)** Cs_2CO_3 (1.0 equiv), 1,3-dibromopropan (0.50 equiv), *N*,*N*-dimethylformamide, 60 °C, overnight.

General procedure for the synthesis of 1,2-(bis-pyrrolyl)ethanes NDA2^H and NMI2^H. Analogous as described in Reference 2. Analogous as Suitable pyrrole derivative **S1** or **S2** (1.0 equiv), 2.0 equiv of 60% sodium hydride in mineral oil (for **S1**) or 1.0 equiv of cesium carbonate (for **S2**) and *N*,*N*-dimethylformamide were placed in a 25 mL round-bottomed flask equipped with a magnetic stirring bar. After stirring for 30 minutes under nitrogen, ethylene di(*p*-toluenesulfonate) (0.60 equiv) in *N*,*N*-dimethylformamide solution was added and the reaction mixture was heated to 60 °C. Stirring was being continued overnight under inert atmosphere and then the reaction mixture was quenched and diluted with water and small amount of brine, and extracted with chloroform. The combined organic layers were dried over anhydrous sodium sulfate, filtred and evaporated under reduced pressure.

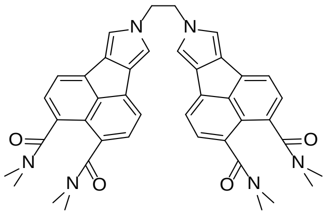

8,8'-(Ethane-1,2-diyl)bis(*N*^{3},*N*^{3},*N*^{4},*N*^{4}-tetramethyl-8*H*-acenaphtho[1,2-*c*]pyrrole-3,4-dicarboxamide) (NDA2^H). Prepared via general procedure using pyrrole **S1** (0.30 g, 0.90 mmol), NaH (0.072 g, 1.80 mmol) in *N*,*N*-dimethylformamide (7.0 mL), and ethylene di(*p*-toluenesulfonate) (0.20 g, 0.54 mmol) in 3.0 mL of *N*,*N*-dimethylformamide. The crude product was purified on column chromatography (silica gel, 1% MeOH w DCM). The third fraction was collected and stripped of solvent on rotary evaporator. Subsequently, the product was recrystallized from CH_2Cl_2/*n*-hexane yielding beige solid. (0.175 g, 56%). **^{1}H NMR** (600 MHz, chloroform-*d* , 300 K): δ 7.40 (1H, m), 7.29 (1H, d, ^3J = 7.2 Hz), 6.68 (1H, m), 4.26 (1H, s), 3.06 (3H, s) 2.87 (3H, s). **^{13}C NMR** (151 MHz, chloroform-*d* , 300 K): δ 171.12 137.69 134.81 131.41 128.83 127.28 124.64 118.32 114.40 114.35 51.70 39.36 34.81. **HRMS** (MALDI–TOF): *m/z*: $[M + Na]^+$ Calcd for $C_{42}H_{40}N_6O_4$: 715.3003; Found 715.3133.

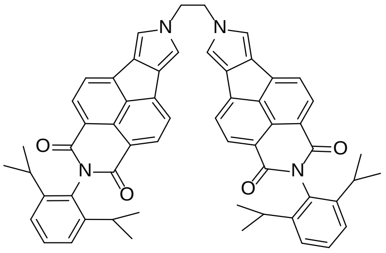

7,7'-(Ethane-1,2-diyl)bis(2-(2,6-diisopropylphenyl)pyrrolo[3',4':2,3]indeno[6,7,1-*def*]isoquinoline-1,3(2*H*,7*H*)-dione) (NMI2^{H}). Prepared via general procedure using pyrrole **S2** (0.15 g, 0.35 mmol), Cs_2CO_3 (0.14 g, 0.42 mmol) in *N*,*N*-dimethylformamide (10.0 mL), and ethylene di(*p*-toluenesulfonate) in 3.0 mL of *N*,*N*-dimethylformamide. The crude product was recrystallized from CH_2Cl_2/*n*-hexane yielding a yellow-brownish solid (100.0 mg, 65%). **^{1}H NMR** (600 MHz, chloroform-*d* , 300 K): δ 8.38 (2H, d, ^{3}J = 7.4 Hz), 7.54 (2H, d, ^{3}J = 7.4 Hz), 7.42 (1H, t, ^{3}J = 7.6 Hz), 7.28 (2H, d, ^{3}J = 7.6 Hz), 6.78 (2H, s), 4.30 (4H, s), 2.76 (1H, sept, ^{3}J = 6.9 Hz), 1.13 (12H, d, ^{3}J = 6.9 Hz). **^{13}C NMR** (151 MHz, chloroform-*d* , 300 K): δ 164.14, 145.84, 139.56, 136.29, 132.94, 131.36, 129.66, 129.25, 126.73, 123.84, 120.04, 119.44, 117.18, 51.64, 29.06, 23.98 x2. **HRMS** (MALDI–TOF): *m/z*: [M + Na]$^{+}$ Calcd for $C_{58}H_{50}N_4O_4$: 889.3724; Found 889.3811.

General procedure for the synthesis of 1,3-(bis-pyrrolyl)propanes NDA3^{H} and NMI3^{H}. Analogous as described in Reference 2. The suitable pyrrole derivative **S1** or **S2** (1.0 equiv) and corresponding base (1.0 equiv of cesium carbonate or 2.0 equiv of 60% NaH in mineral oil), and *N*,*N*-dimethylformamide were placed in a 25 mL round-bottomed flask equipped with a magnetic stirring bar. The mixture was kept stirred under inert atmosphere for 30 minutes, until the color of solution changed. Then, 1,3-dibromopropane (0.5 equiv) was added and the reaction mixture was heated to 60 °C. Stirring was continued overnight under nitrogen atmosphere with further heating. The mixture was cooled down to room temperature, diluted with water and a small amount of brine, and extracted with chloroform. The combined organic layers were dried over anhydrous sodium sulfate, filtred and evaporated under reduced pressure.

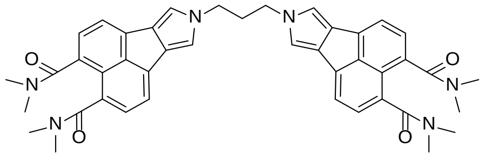

8,8'-(Propane-1,3-diyl)bis(*N*^{3},*N*^{3},*N*^{4},*N*^{4}-tetramethyl-8*H*-acenaphtho[1,2-*c*]pyrrole-3,4-dicarboxamide) (NDA3^{H}). Prepared via general procedure using pyrrole **S1** (0.25 g, 0.75 mmol), NaH (60.0 mg, 1.50 mmol) in *N*,*N*-dimethylformamide (10.0 mL), and 1,3-dibromopropane (38.2 uL, 0.38 mmol). The crude product was purified by column chromatography (silica gel, 4% of methanol in dichloromethane) and then recrystallized from CH_2Cl_2–hexane mixture yielding a brown powder (0.22 g, 84%). **^{1}H NMR** (600 MHz, chloroform-*d* , 300 K): δ 7.47 (2H, d, ^{3}J = 7.1 Hz), 7.34 (2H, d, ^{3}J = 7.1 Hz), 6.85 (2H, s), 3.97 (2H, t, ^{3}J = 6.6 Hz), 3.07 (6H, s) 2.86 (6H, s), 2.38 (1H, quint, ^{3}J = 6.6 Hz). **^{13}C NMR** (151 MHz, chloroform-*d* , 300 K): δ 171.08 137.54 134.94 131.41 128.50 127.36 124.62 118.22 114.29 46.79 39.33 34.82 32.65. **HRMS** (MALDI–TOF): *m/z*: [M + Na]$^{+}$ Calcd for $C_{43}H_{42}N_6O_4$: 729.3160; Found 729.3197.

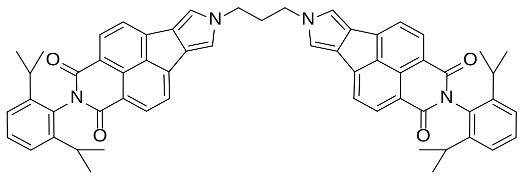

7,7'-(Propane-1,3-diyl)bis(2-(2,6-diisopropylphenyl)pyrrolo[3',4':2,3]indeno[6,7,1-*def*]isoquinoline-1,3(2*H*,7*H*)-dione) (NMI3^{H}).Prepared via general procedure using pyrrole **S2** (0.10 g, 0.24 mmol), Cs_2CO_3 (90 mg, 0.28 mmol) in *N*,*N*-dimethylformamide (10.0 mL), and 1,3-dibromopropane (12.5 µL, 0.12 mmol). The crude product was recrystallized from CH_2Cl_2/*n*-hexane a yielding a yellow solid. (91.0 mg, 87%). **^{1}H NMR** (500 MHz, chloroform-*d* , 300 K): δ 8.41 (2H, d, ^{3}J = 7.3 Hz), 7.60 (2H, d, ^{3}J = 7.3 Hz), 7.43 (1H, t, ^{3}J = 7.8 Hz), 7.29 (2H, d, ^{3}J = 7.8 Hz), 6.92 (2H, s), 4.01 (2H, t, ^{3}J = 6.7 Hz), 2.78 (2H, sept, ^{3}J = 6.8 Hz), 2.44 (1H, quint, ^{3}J = 6.7 Hz), 1.15 (6H, s), 1.13 (6H, s). **^{13}C NMR** (151 MHz, chloroform-*d* , 300 K): δ 164.20, 145.88, 139.75, 136.25, 132.97, 131.41, 129.33, 129.23, 126.74, 123.83, 120.00, 119.28, 117.15, 76.99, 47.02, 31.57, 29.06, 23.98, 22.63, 14.07. **HRMS** (MALDI–TOF): *m/z*: [M + Na]$^{+}$ Calcd for $C_{59}H_{52}N_4O_4$: 903.3881; Found 903.3932.

General procedure for the synthesis of lactams (cNDA2^{O}, cNMI2^{O}, cNDA3^{O}, and cNMI3^{O}) and boomerangs cNMI2^{H} and cNMI3^{H}. To a pressure tube suitable dipyrrolylalkane (**cNDA2**^{H} or **cNMI2**^{H} or **cNDA3**^{H} or **cNMI3**^{H}), palladium(II) acetate (3.0 equiv), AcOK (6.0 equiv), and glacial acetic acid were added. The pressure tube was then sealed and placed in an oil bath preheated to 120 °C. After 1 h the reaction mixture was cooled down to room temperature, quenched and diluted with water and extracted with chloroform. The combined organic layers were washed with water solution of sodium bicarbonate, then dried over anhydrous sodium sulfate, filtred, and evaporated under reduced pressure.

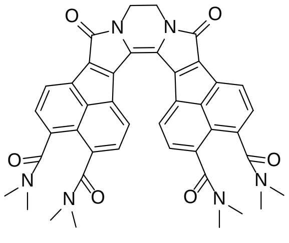

***N*^{1},*N*^{1},*N*^{12},*N*^{12},*N*^{13},*N*^{13},*N*^{18},*N*^{18}-Octamethyl-4,9-dioxo-4,6,7,9-tetrahydroacenaphtho [1',2':3,4]pyrrolo [1,2-*a*]acenaphtho[1',2':3,4]pyrrolo[2,1-*c*]pyrazine-1,12,13,18-tetracarboxamide (cNDA2^{O}).** Prepared via general procedure using **NDA2**^{H} (20.0 mg, 29 µmol), palladium(II) acetate (19.0 mg, 88 µmol), and potassium acetate (16.7 mg, 160 µmol) in 1 mL of acetic acid. The crude product was purified by column chromatography (III grade alumina, 1 % methanol in dichloromethane) yielding a dark blue solid (10.6 mg, 53%). **^{1}H NMR** (500 MHz, chloroform-*d* , 300 K): δ 8.21 (1H, t, ^{3}J = 6.9 Hz), 8.05 (1H, d, ^{3}J = 7.1 Hz), 7.48 (1H, d, ^{3}J = 7.1 Hz), 7.31 (1H, d, ^{3}J = 7.4 Hz), 4.46 (1H, b), 3.67 (1H, b), 3.10 3.082 3.078 (6H, 3x s), 2.97 2.95 2.89 (6H, 3x s). **^{13}C NMR** (151 MHz, chloroform-*d* , 300 K): δ 169.79, 169.71, 161.83, 143.35, 143.30, 138.30, 138.20, 138.07, 137.02, 136.94, 134.66, 131.96, 131.84, 131.02, 127.74, 126.54, 126.41, 126.13, 125.64, 124.25, 121.63, 39.41, 39.38, 38.28, 34.81. **HRMS** (MALDI–TOF): *m/z*: [M + Na]$^{+}$ Calcd for $C_{42}H_{36}N_6O_6$: 743.2589; Found 743.2608. **UV-vis** (dichloromethane, 300 K) λ [nm] (ε in M^{-1} cm^{-1}): 390 (18 200), 570 (21 900), 610 (21 600).

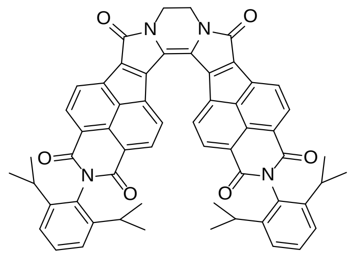

(cNMI2^O). Prepared *via* general procedure using **NMI2**^H (20.0 mg, 24 µmol), palladium(II) acetate (16.9 mg, 72 µmol), and potassium acetate (14.2 mg, 144 µmol) in 1.0 mL of acetic acid. The crude product was purified by column chromatography (V grade alumina, chloroform stabilized with amylene, second fraction) yielding a green solid (12.0 mg, 60%). **^{1}H NMR** (600 MHz, chloroform-*d* , 300 K): δ 8.54 (1H, d, ^3J = 7.6 Hz), 8.39 – 8.34 (2H, m), 8.23 (1H, d, ^3J = 7.1 Hz), 7.47 (1H, t, ^3J = 7.8 Hz), 7.32 (2H, d, ^3J = 8.0 Hz), 4.80 – 3.49 (2H, b), 2.80 – 2.71 (2H, m), 1.15, 1.14 (12H, 2x s). **^{13}C NMR** (151 MHz, chloroform-*d* , 300 K): δ 163.09, 162.94, 161.00, 145.78, 145.26, 135.81, 134.67, 133.15, 133.10, 131.22, 130.39, 129.74, 127.02, 126.81, 125.81, 125.34, 124.15, 124.05, 122.51, 38.32, 29.19, 24.03, 24.00. **HRMS** (ESI–TOF): *m/z*: [M]$^-$ Calcd for $C_{58}H_{48}N_4O_4$: 894.3412; Found 894.3294. **UV-vis** (dichloromethane, 300 K) λ [nm] (ε in M^{-1} cm^{-1}): 410 (20 900), 630 (19 300), 690 (18 600).

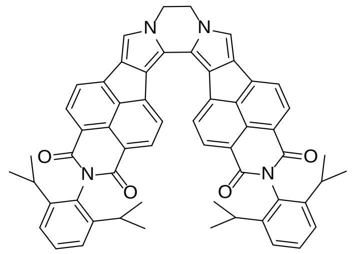

(cNMI2^H). Prepared via general procedure using **NMI2**^H (20.0 mg, 24 µmol), palladium(II) acetate (16.9 mg, 72 µmol) in 6.0 mL of of acetic acid. The crude product was purified by column chromatography (V grade alumina, chloroform stabilized with amylene, first fraction) yielding a reddish solid (13.2 mg, 66%). **^{1}H NMR** (600 MHz, chloroform-*d* , 300 K): δ 8.50 (1H, d, ^3J = 7.3 Hz), 8.48 (1H, d, ^3J = 7.4 Hz), 7.76 (1H, d, ^3J = 7.1 Hz), 7.44 (1H, t, ^3J = 7.7 Hz), 7.30 (2H, d, ^3J = 7.7 Hz), 7.21 (1H, s), 7.03 (1H, d, ^3J = 7.2 Hz), 4.44 (2H, b), 2.81 (2H, b), 1.16 (6H, s), 1.15 (6H, s). **^{13}C NMR** (151 MHz, chloroform-*d* , 300 K): δ 164.22, 164.12, 145.94, 138.93, 138.33, 136.45, 132.82, 132.57, 131.42, 129.29, 129.24, 126.82, 124.93, 123.92, 123.88, 120.31, 120.29, 119.80, 119.63, 118.06, 45.64, 29.10, 24.06, 24.03 . **HRMS** (MALDI–TOF): *m/z*: [M + Na]$^+$ Calcd for $C_{58}H_{48}N_4O_4$: 887.3568; Found 887.3878. **UV-vis** (dichloromethane, 300 K) λ [nm] (ε in M^{-1} cm^{-1}): 360 (12 000), 480 (13 700), 530 (13 200), 570 (17 200).

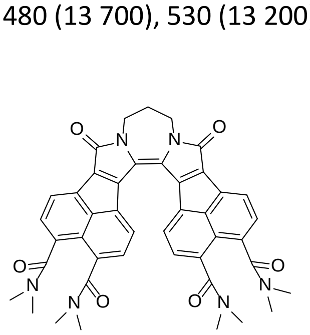

***N*^{1},*N*^{1},*N*^{13},*N*^{13},*N*^{14},*N*^{14},*N*^{19},*N*^{19}-Octamethyl-4,10-dioxo-4,7,8,10-tetrahydro-6*H*-acenaphtho[1',2':3,4] pyrrolo[1,2-*a*]acenaphtho[1',2':3,4]pyrrolo[2,1-*c*][1,4]diazepine-1,13,14,19-tetracarboxamide (cNDA3^O).** Prepared *via* general procedure using **NDA3**^H (20.0 mg, 27 µmol), palladium(II) acetate (18.7 mg, 81 µmol), and potassium acetate (16.7 mg, 160 µmol) in 1 mL of acetic acid. The crude product

was purified by column chromatography (III grade alumina, 1 % methanol in dichloromethane) yielding a blue solid (12.7 mg, 64%). **^{1}H NMR** (500 MHz, chloroform-*d* , 300 K): δ 8.07-7.89 (4H, m) 7.47 (2H, m) 7.30-7.10 (2H, m), 4.68 (2H, m), 3.49 (2H, m), 3.16-2.76 (24H, m) 2.25 (2H, m). **^{13}C NMR** (151 MHz, chloroform-*d* , 300 K): δ 169.9, 164.3, 144.5, 138.1, 137.6, 137.5, 136.8, 136.3, 132.2, 131.3, 127.8, 127.4, 126.8, 126.6, 126.1, 125.6, 124.3, 39.3, 34.9, 26.7. **HRMS** (MALDI–TOF): *m/z*: $[M + Na]^+$ Calcd for $C_{43}H_{38}N_6O_6$: 757.2745; Found 757.2826. **UV-vis** (dichloromethane, 300 K) λ [nm] (ε in M^{-1} cm^{-1}): 390 (13 700), 590 (14 000), 625 (14 200).

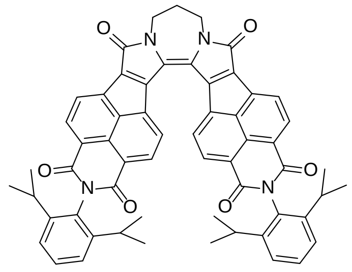

(cNMI3^O). Prepared *via* general procedure using **NMI3**^{H} (20.0 mg, 23 µmol), palladium(II) acetate (16.2 mg, 68 µmol), and potassium acetate (13.4 mg, 136 µmol) in 1 mL of glacial acetic acid. The crude product was purified by column chromatography (V grade alumina, chloroform stabilized with amylene, second fraction) yielding a green solid (14.6 mg, 73%). **^{1}H NMR** (600 MHz, chloroform-*d* , 300 K): δ 8.53 (1H, d, ^3J = 7.1 Hz), 8.25 (1H, d, ^3J = 7.4 Hz), 8.22 (1H, d, ^3J = 7.1 Hz), 8.12 (1H, d, ^3J = 7.4 Hz), 7.46 (1H, t, ^3J = 7.8 Hz), 7.32 – 7.28 (4H, m), 4.77 – 4.71 (2H, m), 3.58 – 3.51 (2H, m), 2.78 (1H, sept, ^3J = 6.8 Hz), 2.69 (1H, sept, ^3J = 6.8 Hz), 2.34 – 2.28 (1H, b), 1.18 1.17 (3H, 2x s), 1.16 1.15 (3H, 2x s), 1.13 1.12 (3H, 2x s), 1.1 1.10 (3H, 2x s). **^{13}C NMR** (600 MHz, chloroform-*d* , 300 K): δ 163.19, 163.12, 162.98, 146.32, 145.83, 145.79, 139.58, 135.89, 134.94, 133.54, 133.19, 131.21, 130.46, 129.71, 127.97, 127.76, 126.65, 125.78, 125.10, 124.11, 124.05, 124.01, 39.78, 29.21, 29.16, 24.091, 24.071, 23.99, 23.98. **HRMS** (MALDI–TOF): *m/z*: $[M + Na]^+$ Calcd for $C_{59}H_{48}N_4O_6$: 931.3466; Found 931.3582. **UV-vis** (dichloromethane, 300 K) λ [nm] (ε in M^{-1} cm^{-1}): 390 (17 500), 405 (18 800), 650 (15 500), 705 (16 000).

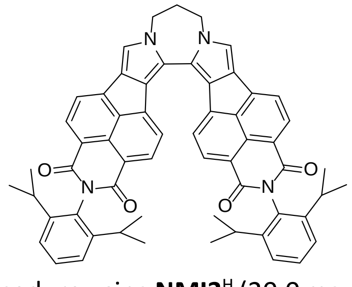

(cNMI3^H). Prepared *via* general procedure using **NMI3**^{H} (20.0 mg, 23 µmol), palladium(II) acetate (16.2 mg, 68 µmol) in 6 mL of acetic acid. The crude product was purified by column chromatography (V grade alumina, chloroform stabilized with amylene, first fraction) yielding a green solid (8.0 mg, 41%). **^{1}H NMR** (600 MHz, chloroform-*d* , 300 K): δ 8.48 (1H, d, ^3J = 7.3 Hz), 8.32 (1H, d, ^3J = 7.3 Hz), 7.74 (1H, d, ^3J = 7.3 Hz), 7.43 (1H, t, ^3J = 7.8 Hz), 7.31 – 7.27 (2H, m), 7.18 (1H, s), 6.93 (1H, d, ^3J = 7.3 Hz), 4.26 – 4.22 (2H, m), 2.83 (1H, sept, ^3J = 7.0 Hz), 2.77 (1H, sept, ^3J = 6.9 Hz), 2.56 – 2.49 (1H, m), 1.19, 1.18, 1.16, 1.15, 1.14, 1.13, 1.12, 1.11 (12 x 1H, s). **^{13}C NMR** (600 MHz, chloroform-*d* , 300 K): δ 164.21, 164.14, 145.94, 145.81, 139.25, 138.44, 136.45, 132.92, 132.72, 131.35, 129.26, 128.43, 128.02, 126.68, 123.91, 123.79, 122.29, 121.64, 120.09, 119.93, 119.53, 119.45, 45.56, 29.69, 29.08, 29.02, 24.10, 24.06, 24.03, 24.01. **HRMS** (MALDI–TOF): *m/z*: $[M + Na]^+$ Calcd for $C_{59}H_{50}N_4O_4$: 901.3724; Found

901.3790. **UV-vis** (dichloromethane, 300 K) λ [nm] (ε in M^{-1} cm^{-1}): 360 (13 100), 470 (19 400), 510 (17 000), 545 (19 200).

Additional figures

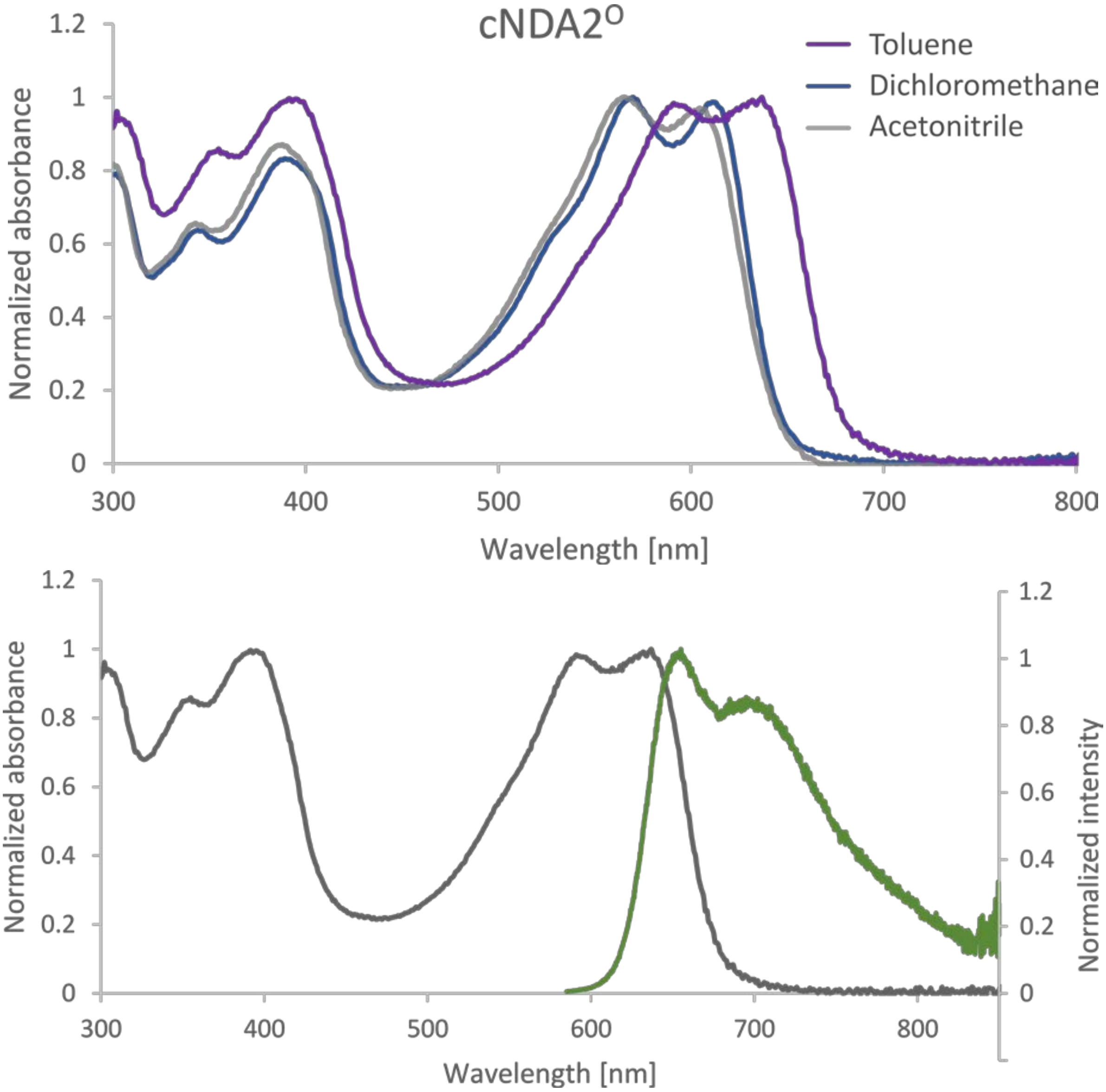

Figure S1. Top: absorption spectra in toluene, dichloromethane and acetonitrile; bottom: absorption and emission spectra in toluene measured at 293 K for **cNDA2^{O}**.

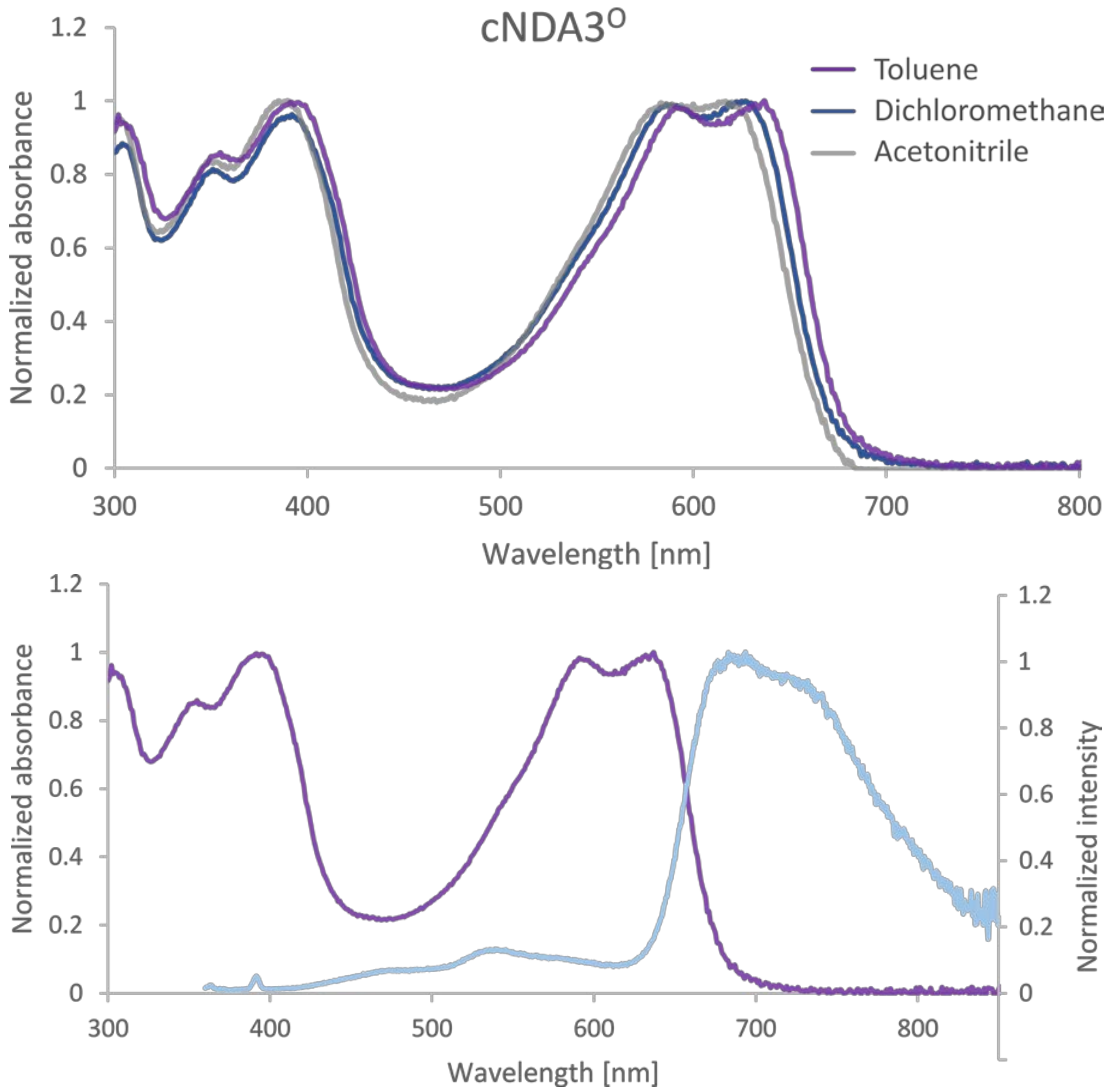

Figure S2. Top: absorption spectra in toluene, dichloromethane and acetonitrile; bottom: absorption and emission spectra in toluene measured at 293 K for **cNDA3^{O}**.

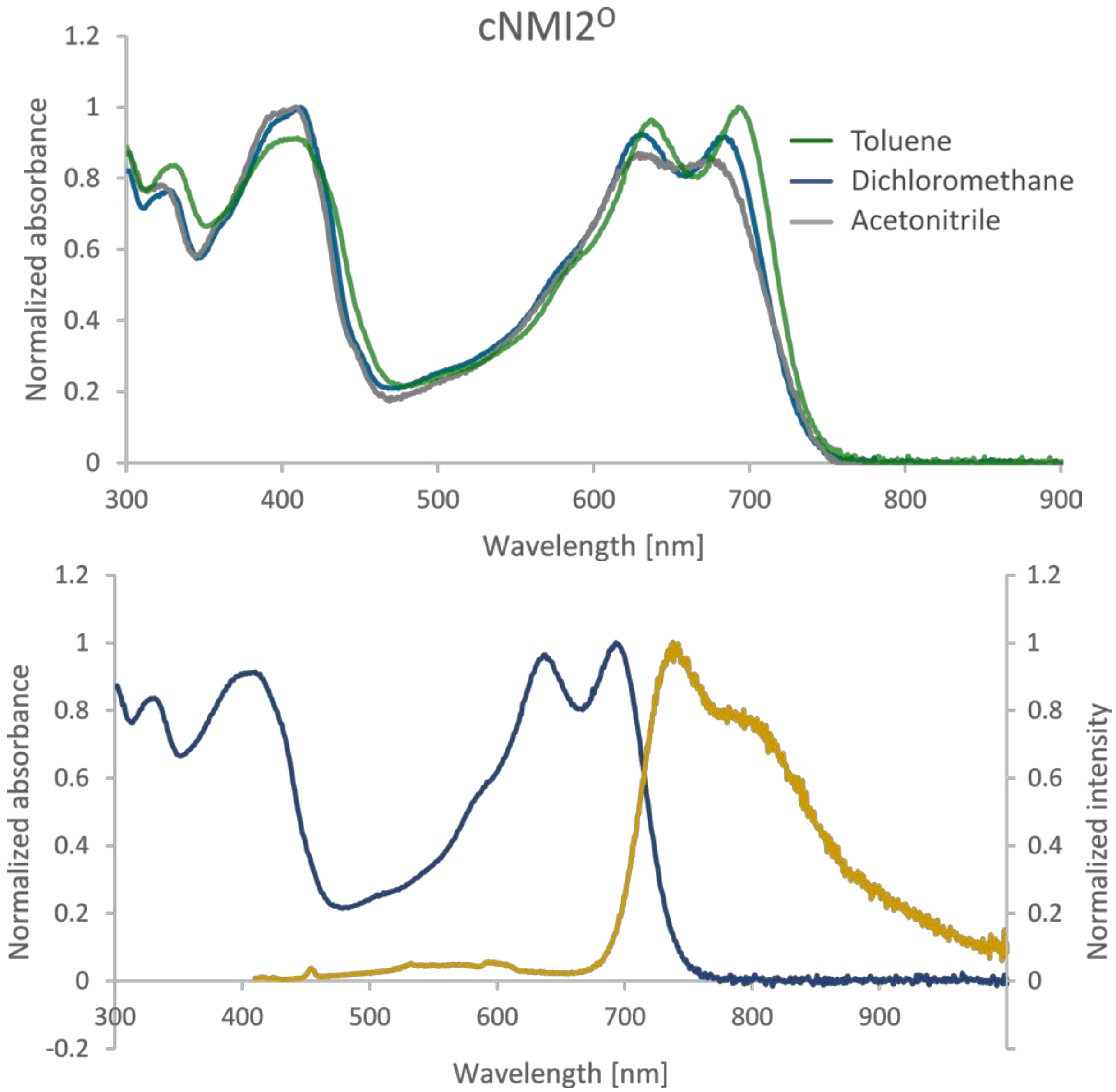

Figure S3. Top: absorption spectra in toluene, dichloromethane and acetonitrile measured at 293 K for **cNMI2^{O}** . Bottom: absorption and emission spectra in toluene measured for **cNMI2^{O}**.

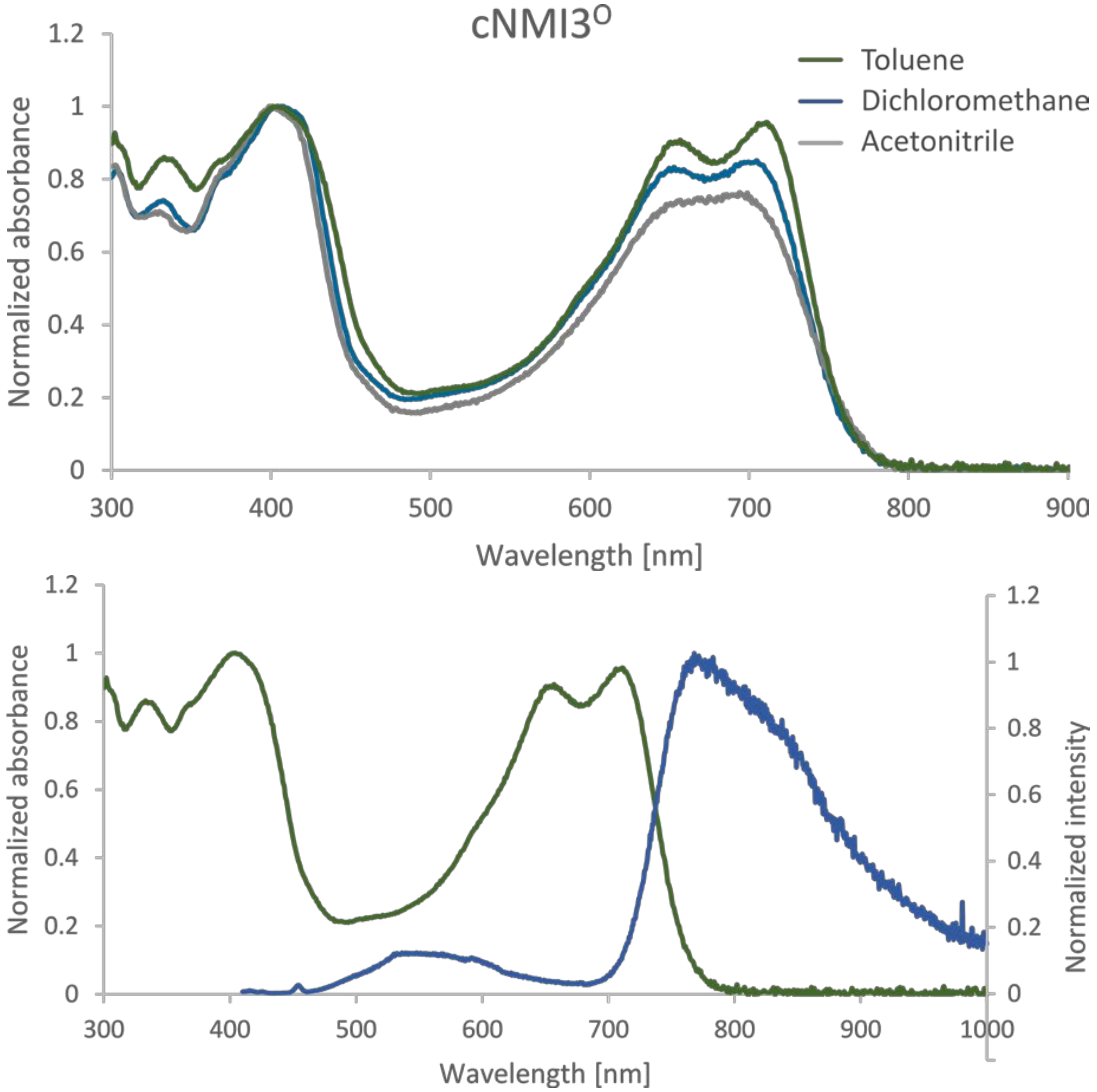

Figure S4. Top: absorption spectra in toluene, dichloromethane and acetonitrile; bottom: absorption and emission spectra in toluene measured at 293 K for **cNMI3^{O}**.

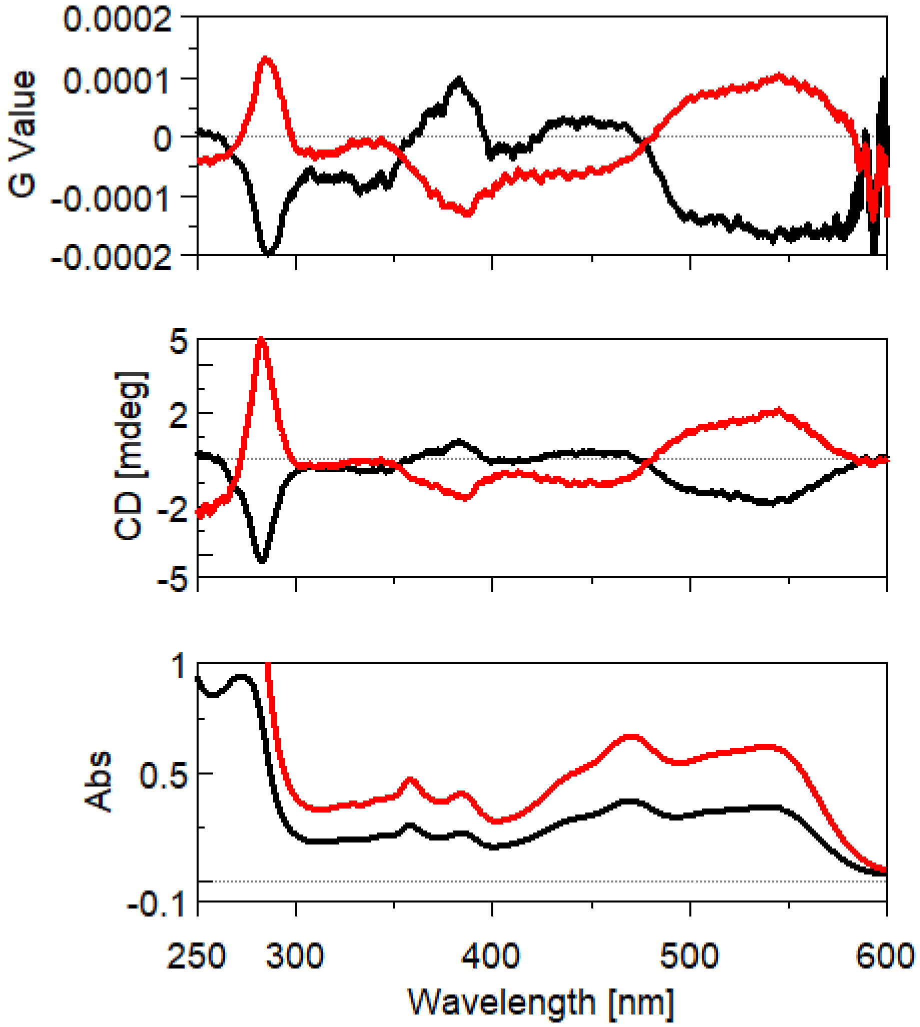

Figure S5. Circular dichroism of **cNMI3^H** in dichloromethane: (two from top) with correction of enantiomeric excess obtained by integration of HPLC signals; (bottom) UV–vis spectrum with molar absorptivity.

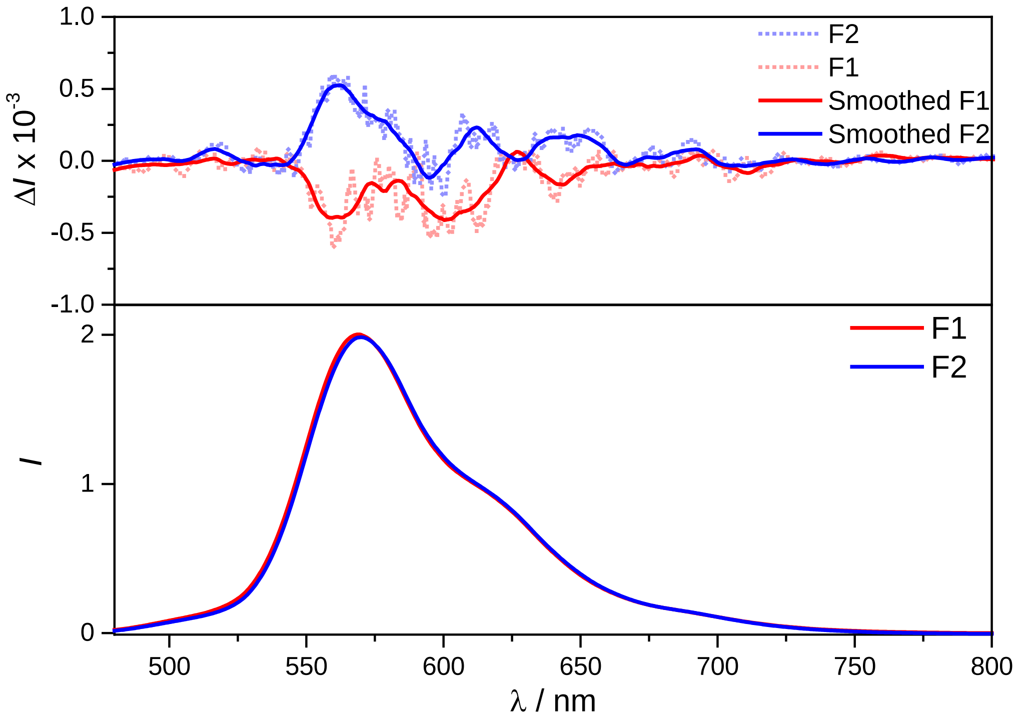

Figure S6. Circularly polarized luminescence recorded for enantioenriched samples of **cNMI3**^{H} (toluene).

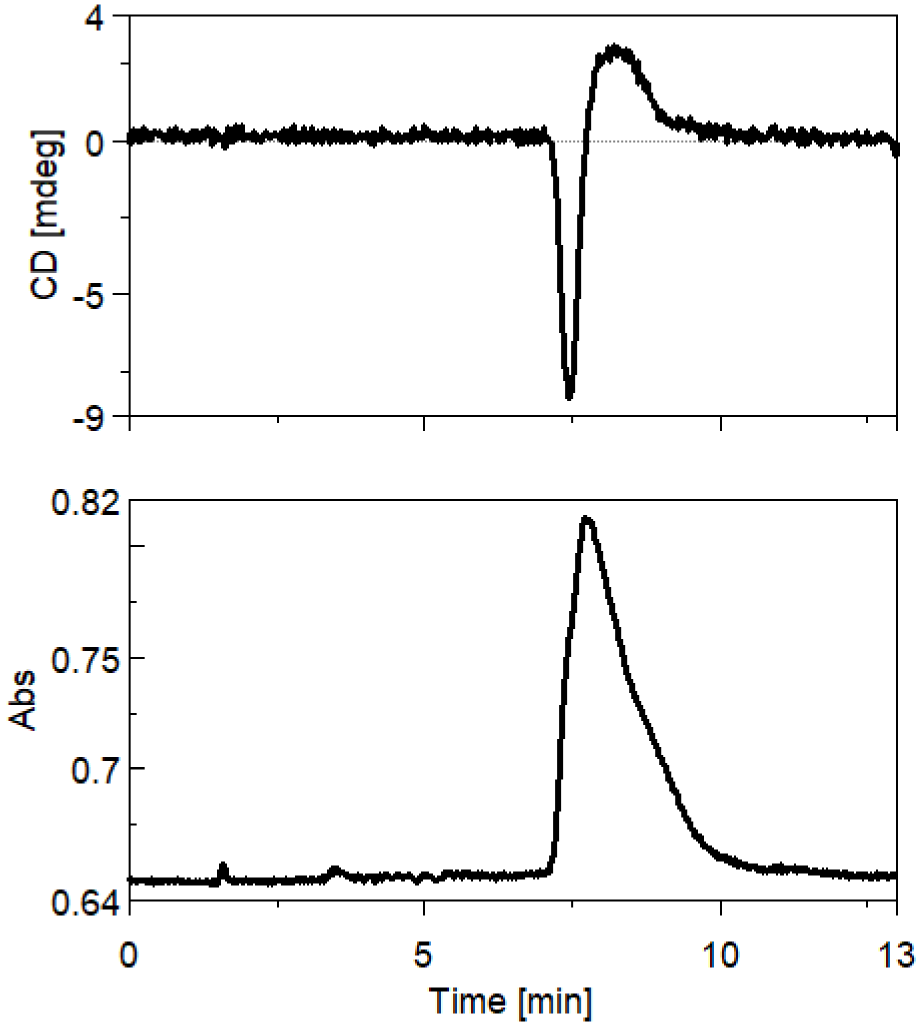

Figure S7. HPLC of **cNMI3**$^{\mathbf{H}}$ DCM/benzene (ratio: 50/50); eluen flow: 2 mL/min; detection of absorbance at 543 nm .

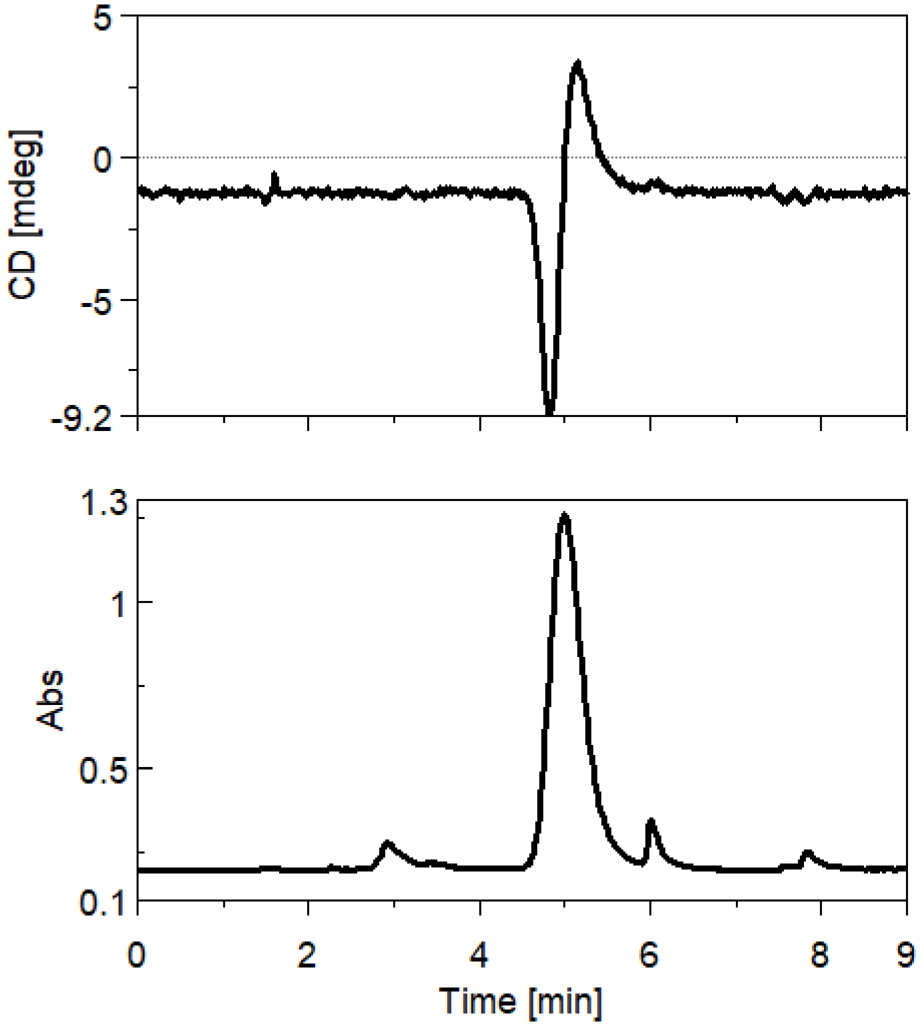

Figure S8. HPLC of **cNDA3°** dichloromethane/methanol (ratio 97/3); eluent flow 2 mL/min; absorbance detected at 397 nm.

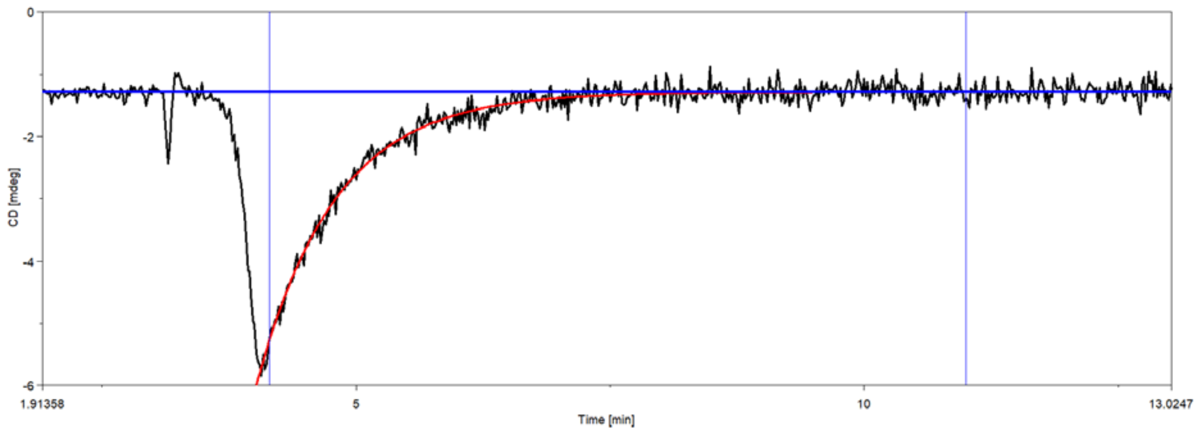

Figure S9. Scheme showing the rapid decreace of enantio excess of separated isomer for **cNDA3°**. Rate equation: Y(t) = −846.935 exp(−t / 0.773951). Time constant: 0.773951 [min]. Rate constant: 1.29207 [min^{-1}] Half-life: 0.536462 [min]. [Parameters]: reaction steps:1; baseline equation: Y(t) = −1.28803; reaction range: 4.15–11 [min]; base range 11 [min].

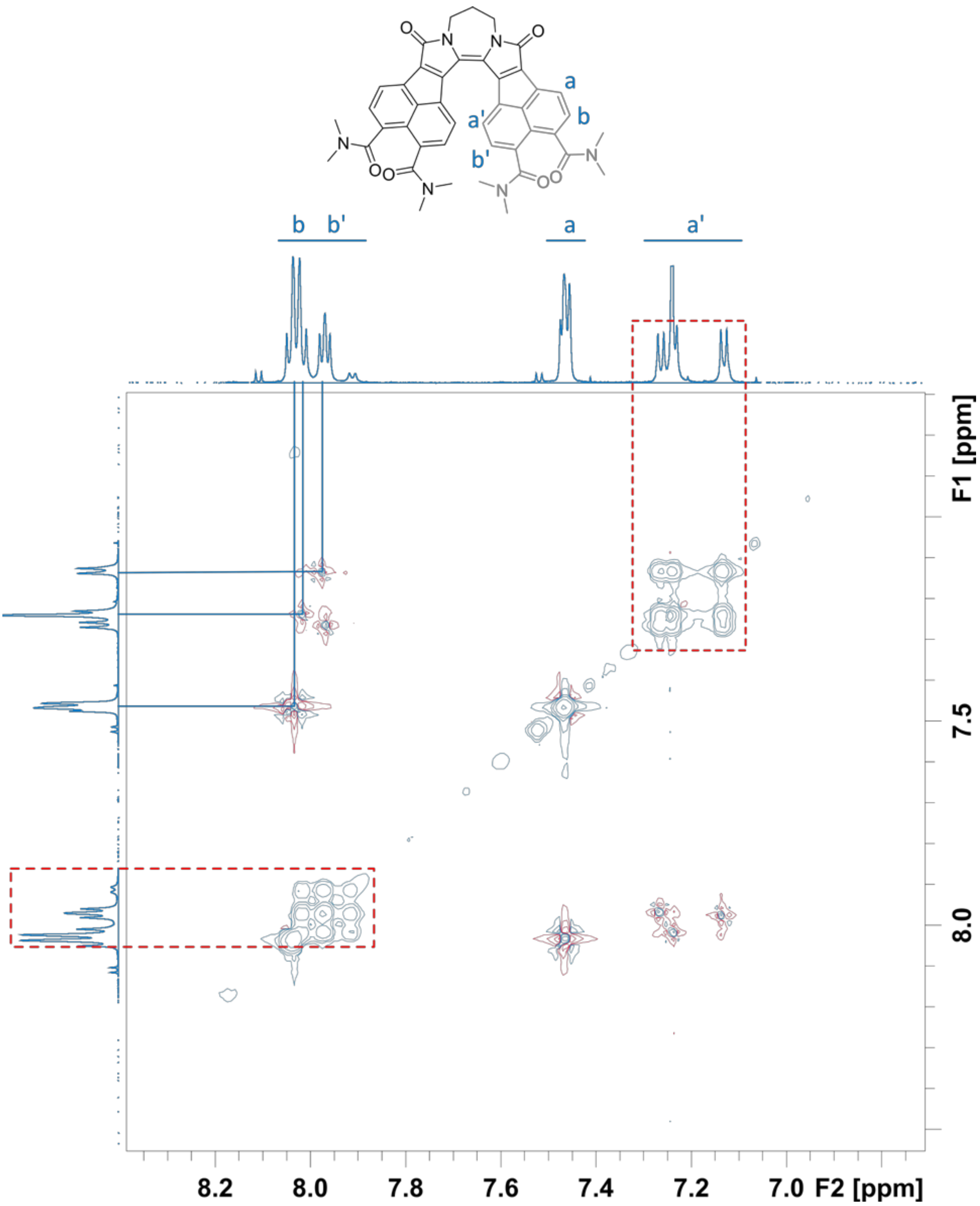

Figure S10. NOESY NMR spectrum of **cNDA3°** (600 MHz, chloroform-*d*, 300K). Red boxes indicate the active exchange peaks corresponding to few (at least three) different conformers. Blue lines shows the correlation peaks.

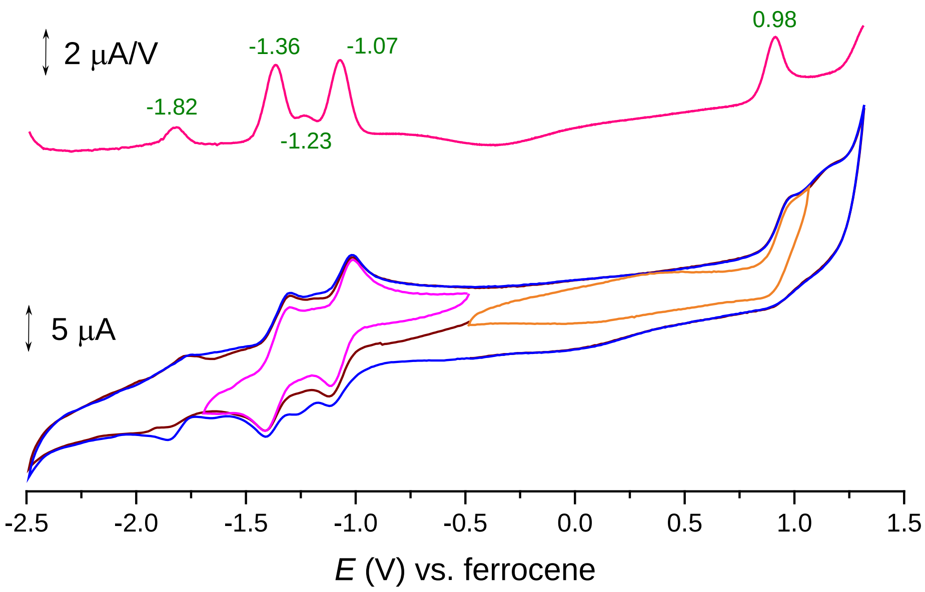

Figure S11. Top: differential pulse voltammetry of **cNDA2^O^**. Bottom: Two consecutive cyclic voltammograms of **cNDA2^O^** were measured under following conditions: $[BuN_4]PF_6$ in dichloromethane, scan rate: 100 mV s^{-1}; the voltammograms were referenced with ferrocene/ferrocenium couple as an internal standard.

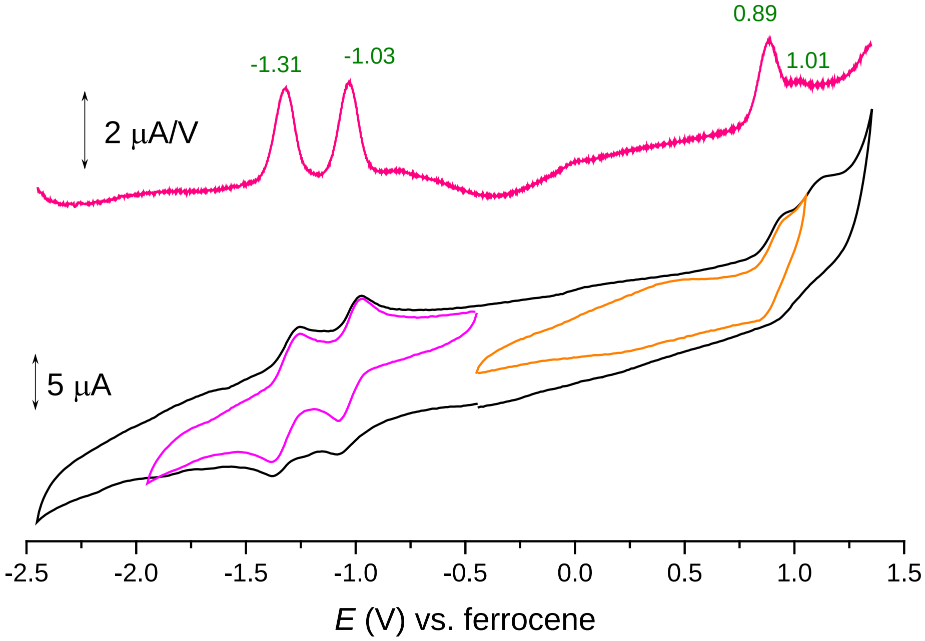

Figure S12. Top: differential pulse voltammetry of **cNDA3^O^**. Bottom: Two consecutive cyclic voltammograms of **cNDA3^O^** were measured under following conditions: $[BuN_4]PF_6$ in dichloromethane, scan rate: 100 mV s^{-1}; the voltammograms were referenced with ferrocene/ferrocenium couple as an internal standard.

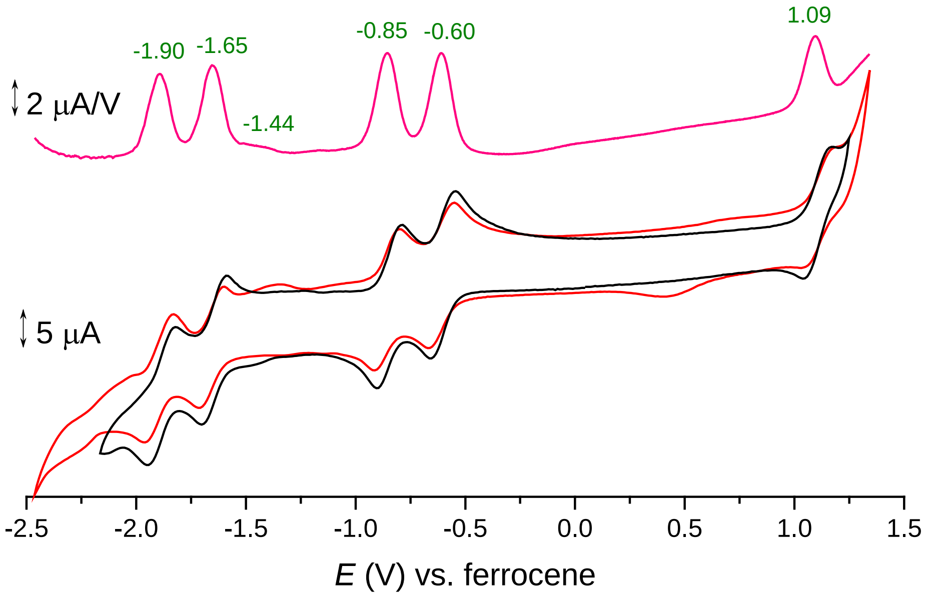

Figure S13. Top: differential pulse voltammetry of **cNMI2^{O}**. Bottom: Two consecutive cyclic voltammograms of **cNMI2^{O}** were measured under following conditions: $[BuN_4]PF_6$ in dichloromethane, scan rate: 100 mV s^{-1}; the voltammograms were referenced with ferrocene/ferrocenium couple as an internal standard.

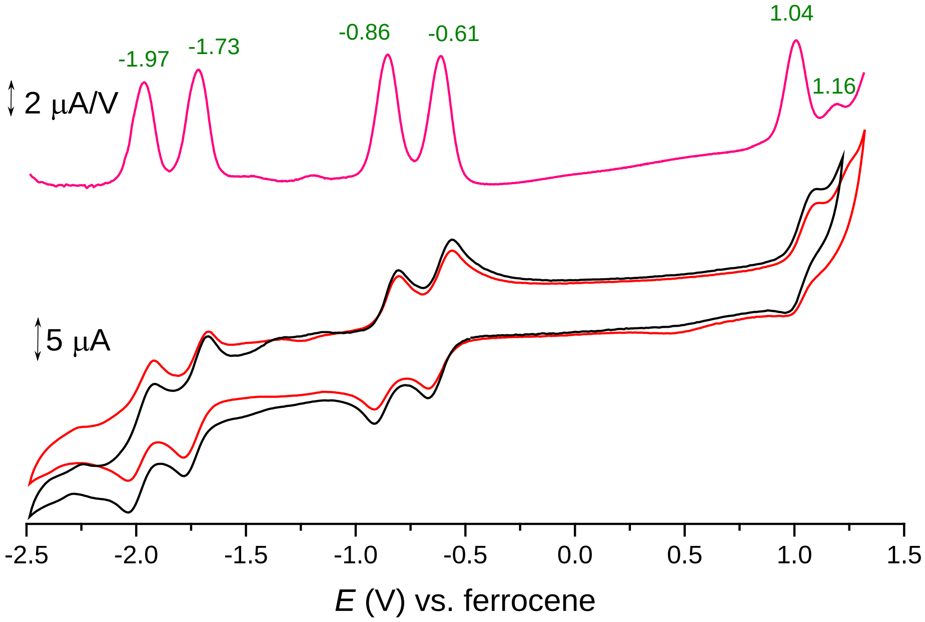

Figure S14. Top: differential pulse voltammetry of **cNMI3^{O}**. Bottom: Two consecutive cyclic voltammograms of **cNMI3^{O}** were measured under following conditions: $[BuN_4]PF_6$ in dichloromethane, scan rate: 100 mV s^{-1}; the voltammograms were referenced with ferrocene/ferrocenium couple as an internal standard.

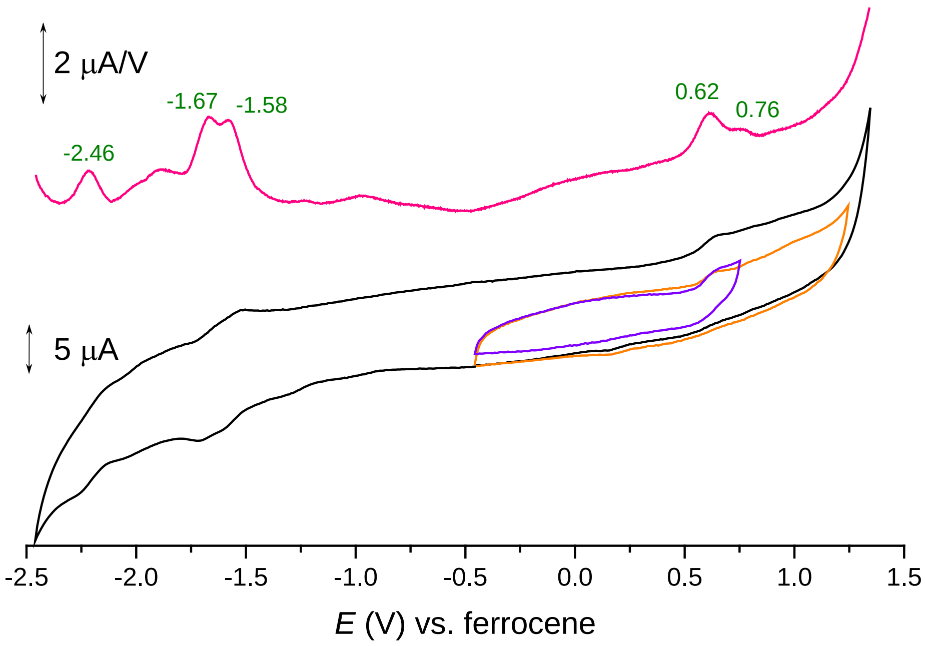

Figure S15. Top: differential pulse voltammetry of **cNMI2^{H}**. Bottom: Two consecutive cyclic voltammograms of **cNMI2^{H}** were measured under following conditions: $[BuN_4]PF_6$ in dichloromethane, scan rate: 100 mV s^{-1}; the voltammograms were referenced with ferrocene/ferrocenium couple as an internal standard.

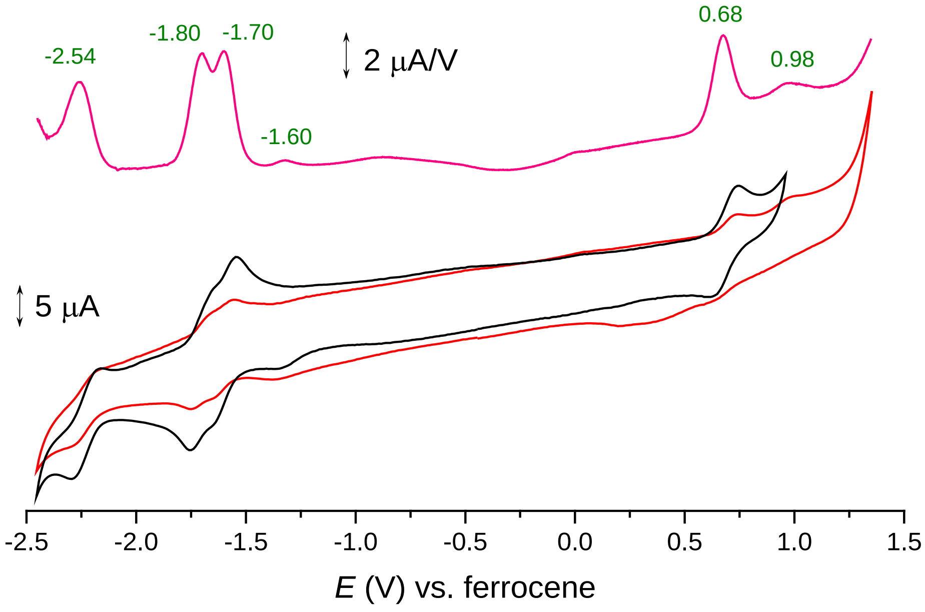

Figure S16. Top: differential pulse voltammetry of **cNMI3^{H}**. Bottom: Two consecutive cyclic voltammograms of **cNMI3^{O}** were measured under following conditions: $[BuN_4]PF_6$ in dichloromethane, scan rate: 100 mV s^{-1}; the voltammograms were referenced with ferrocene/ferrocenium couple as an internal standard.

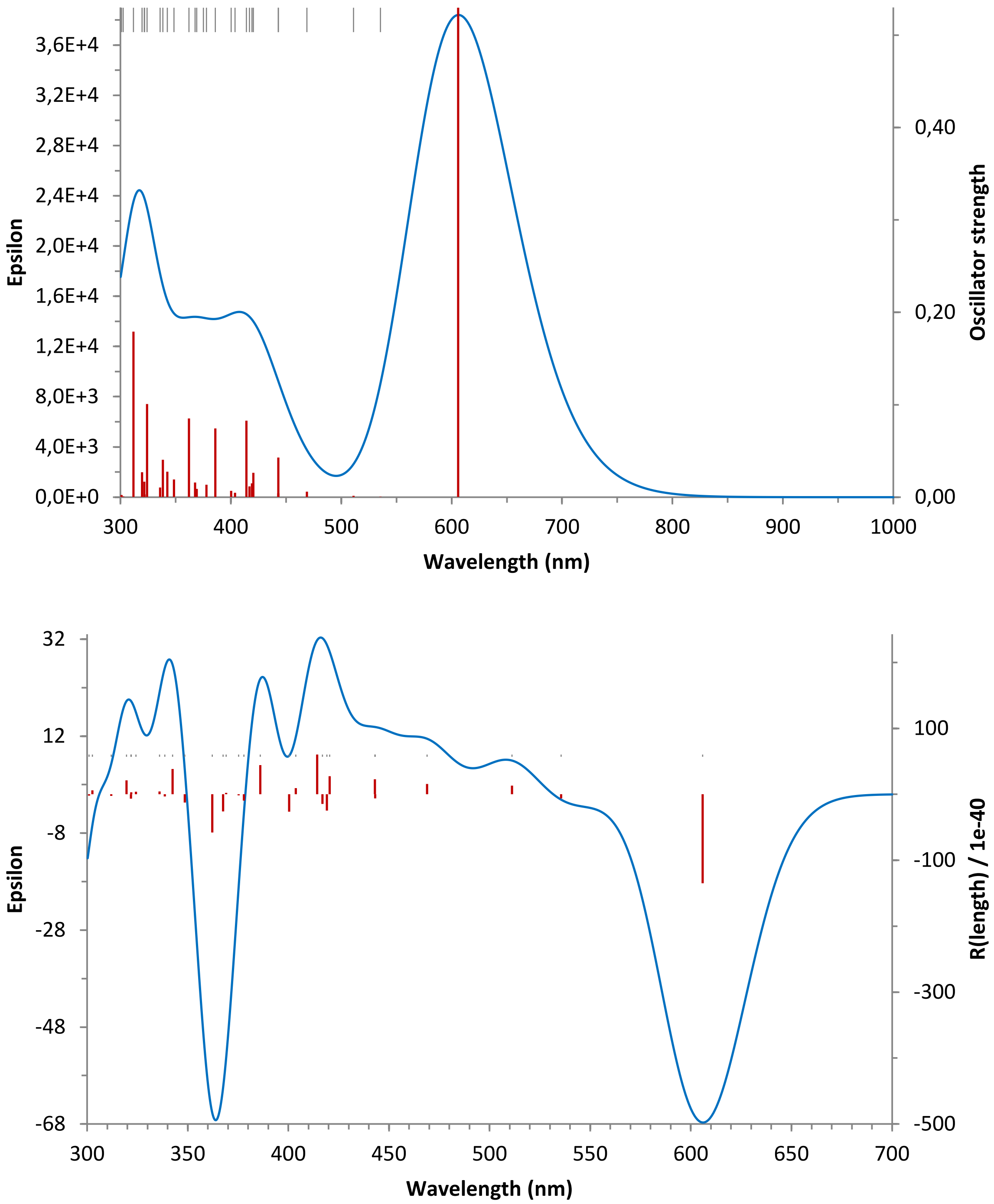

Figure S17. TDA–DFT spectra (top: UV–vis, bottom – CD) for **cNDA2**^{O} (B3LYP/6–31G(d,p)). Individual transitions are shown as red sticks. The blue envelope is a sum of Gaussian profiles with halfwidths of 1500 cm^{-1}.

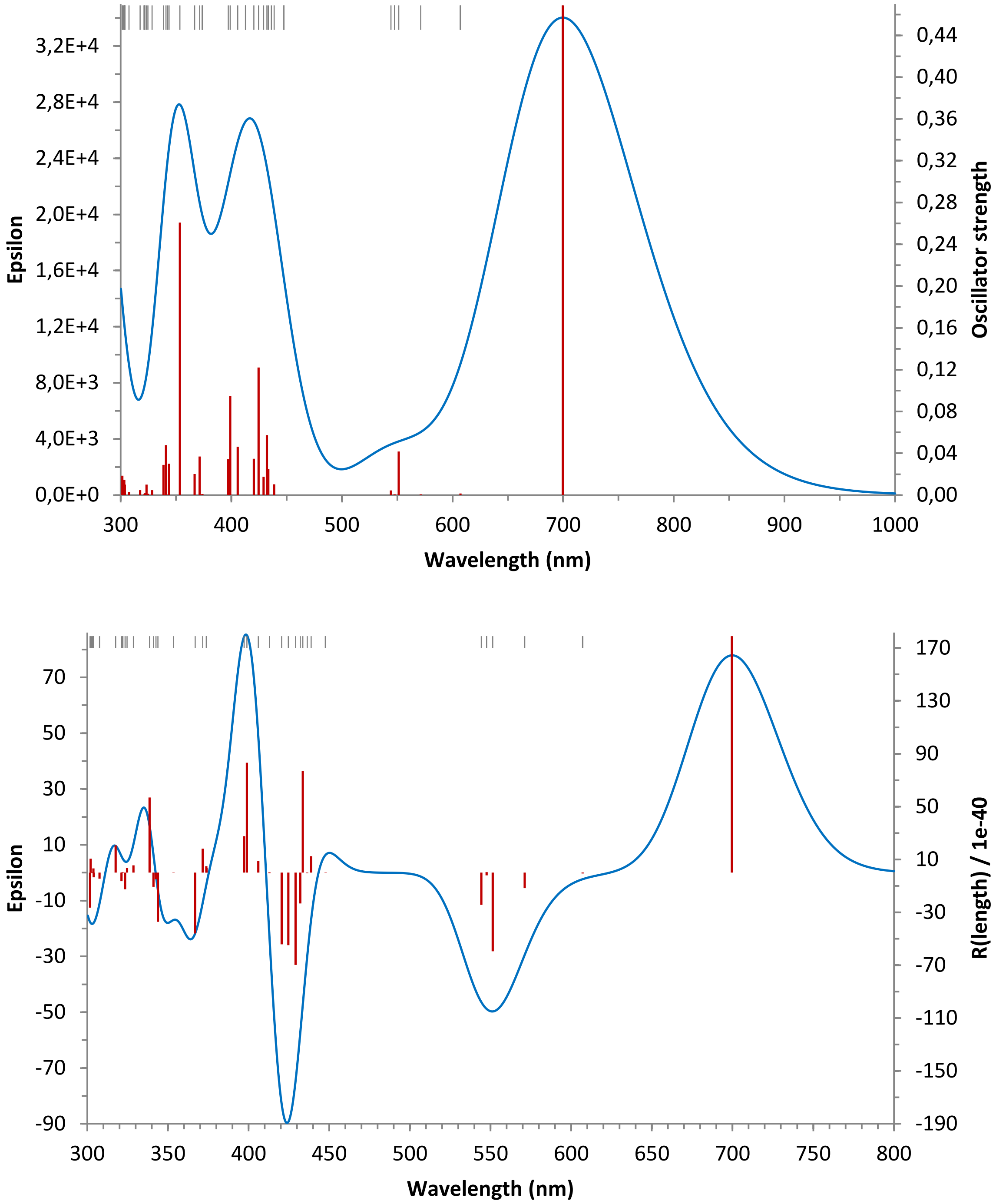

Figure S18. TDA−DFT spectra (top: UV–vis, bottom – CD) for **cNMI2^{O}** (B3LYP/6−31G(d,p)). Individual transitions are shown as red sticks. The blue envelope is a sum of Gaussian profiles with halfwidths of 1500 cm^{-1}.

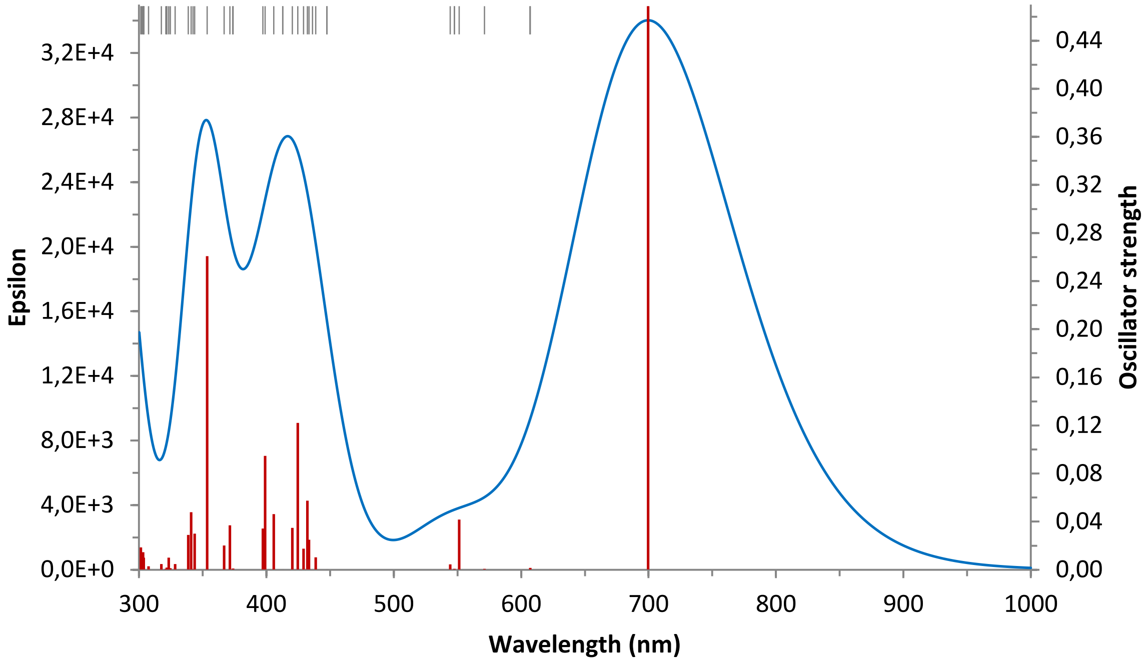

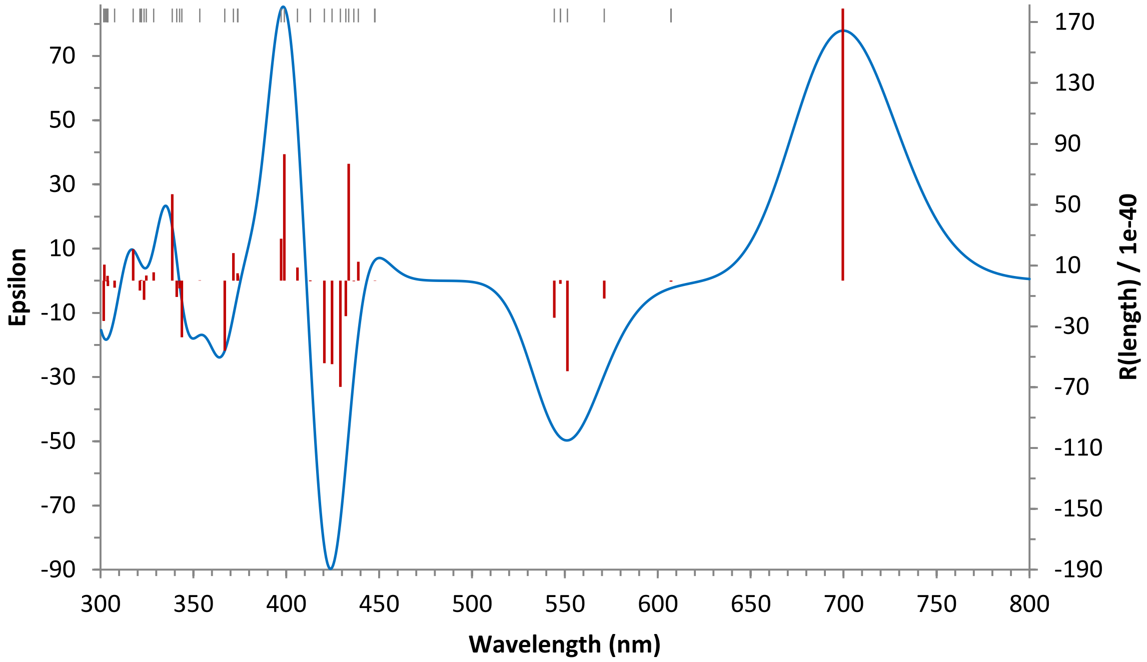

Figure S19. TDA–DFT spectra (top: UV–vis, bottom – CD) for **cNMI2^{H}** (B3LYP/6–31G(d,p)). Individual transitions are shown as red sticks. The blue envelope is a sum of Gaussian profiles with halfwidths of 1500 cm^{-1}.

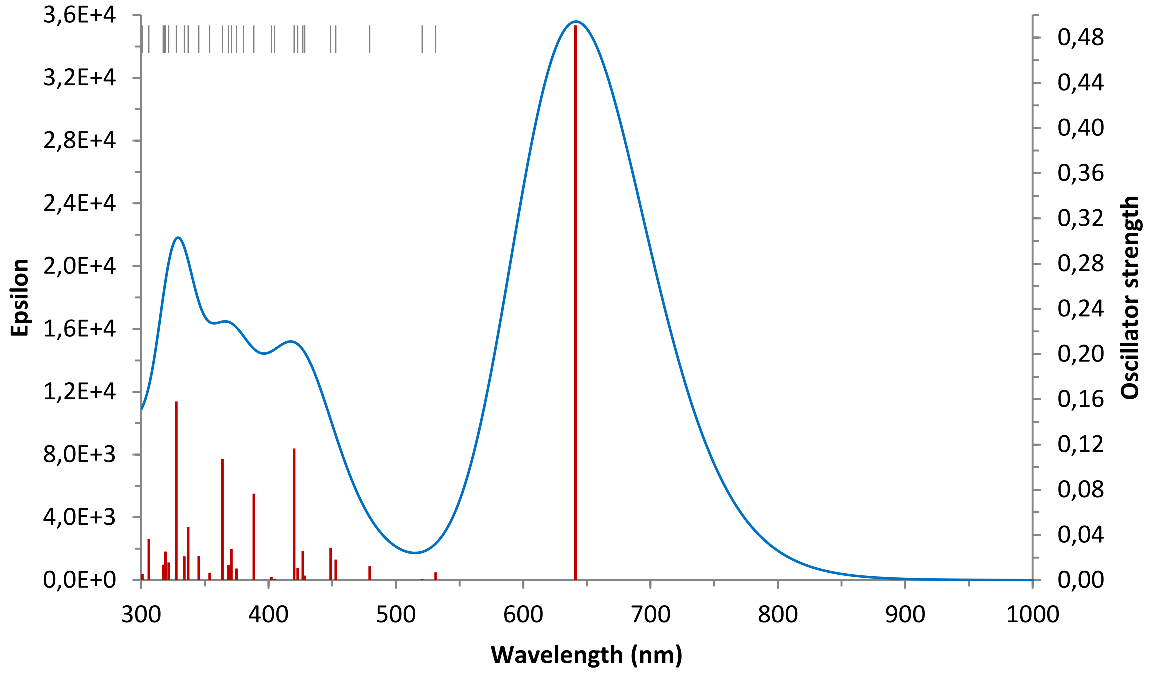

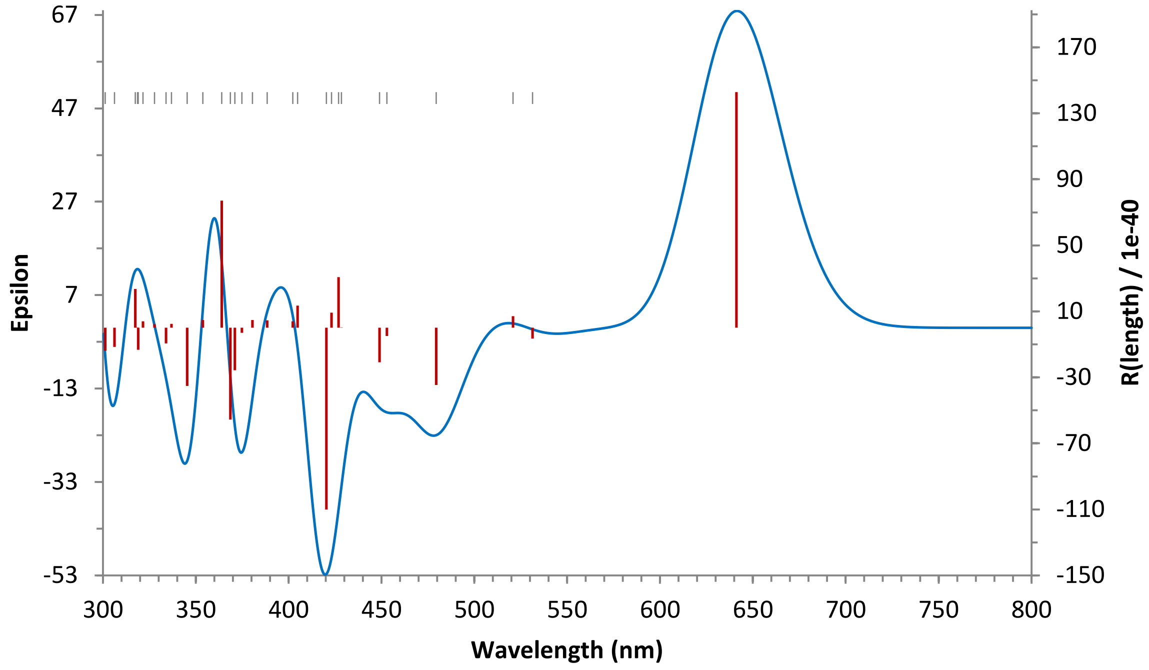

Figure S20. TDA–DFT spectra (top: UV–vis, bottom – CD) for **cNDA3**^{O} (B3LYP/6–31G(d,p)). Individual transitions are shown as red sticks. The blue envelope is a sum of Gaussian profiles with halfwidths of 1500 cm^{-1}.

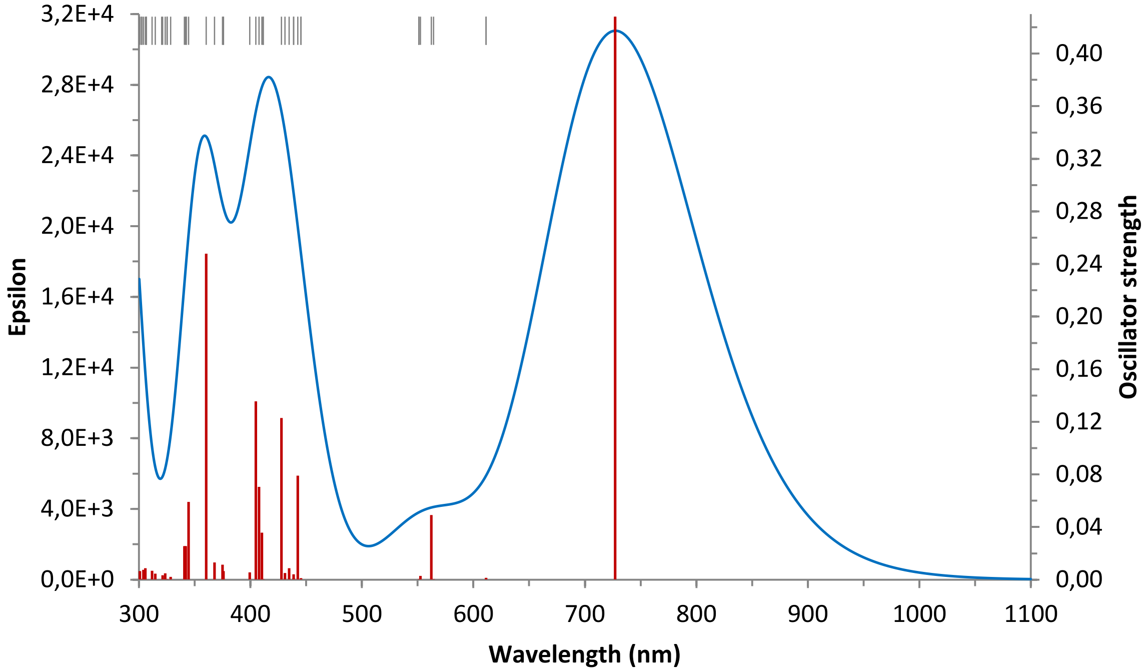

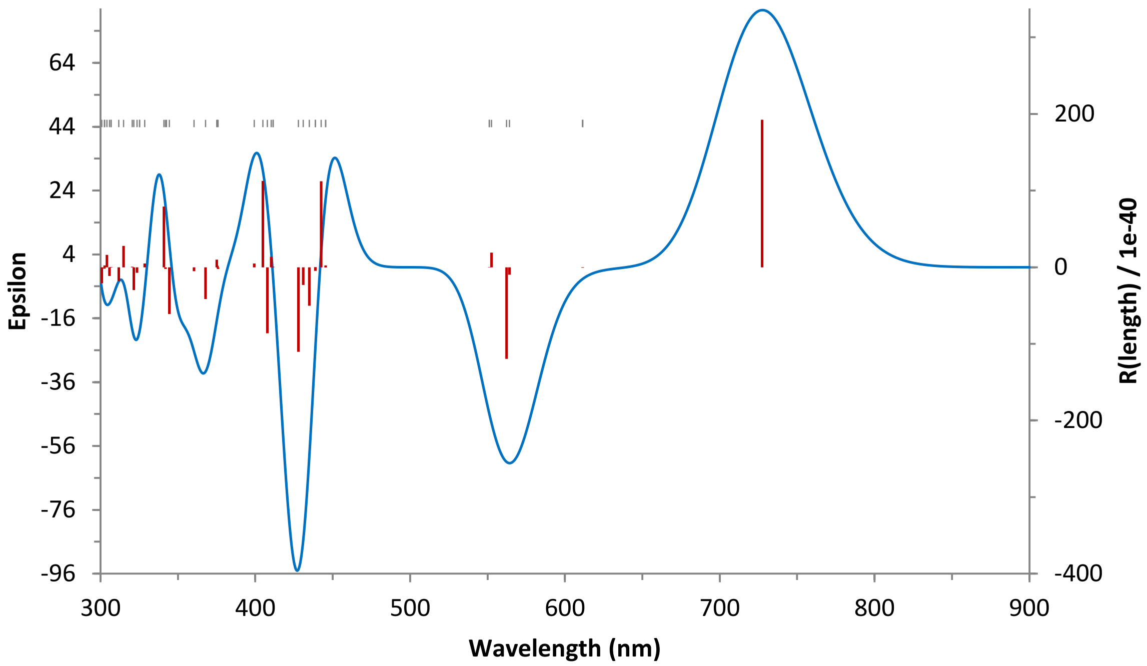

Figure S21. TDA−DFT spectra (top: UV–vis, bottom – CD) for **cNMI3^{O}** (B3LYP/6−31G(d,p)). Individual transitions are shown as red sticks. The blue envelope is a sum of Gaussian profiles with halfwidths of 1500 cm^{-1}.

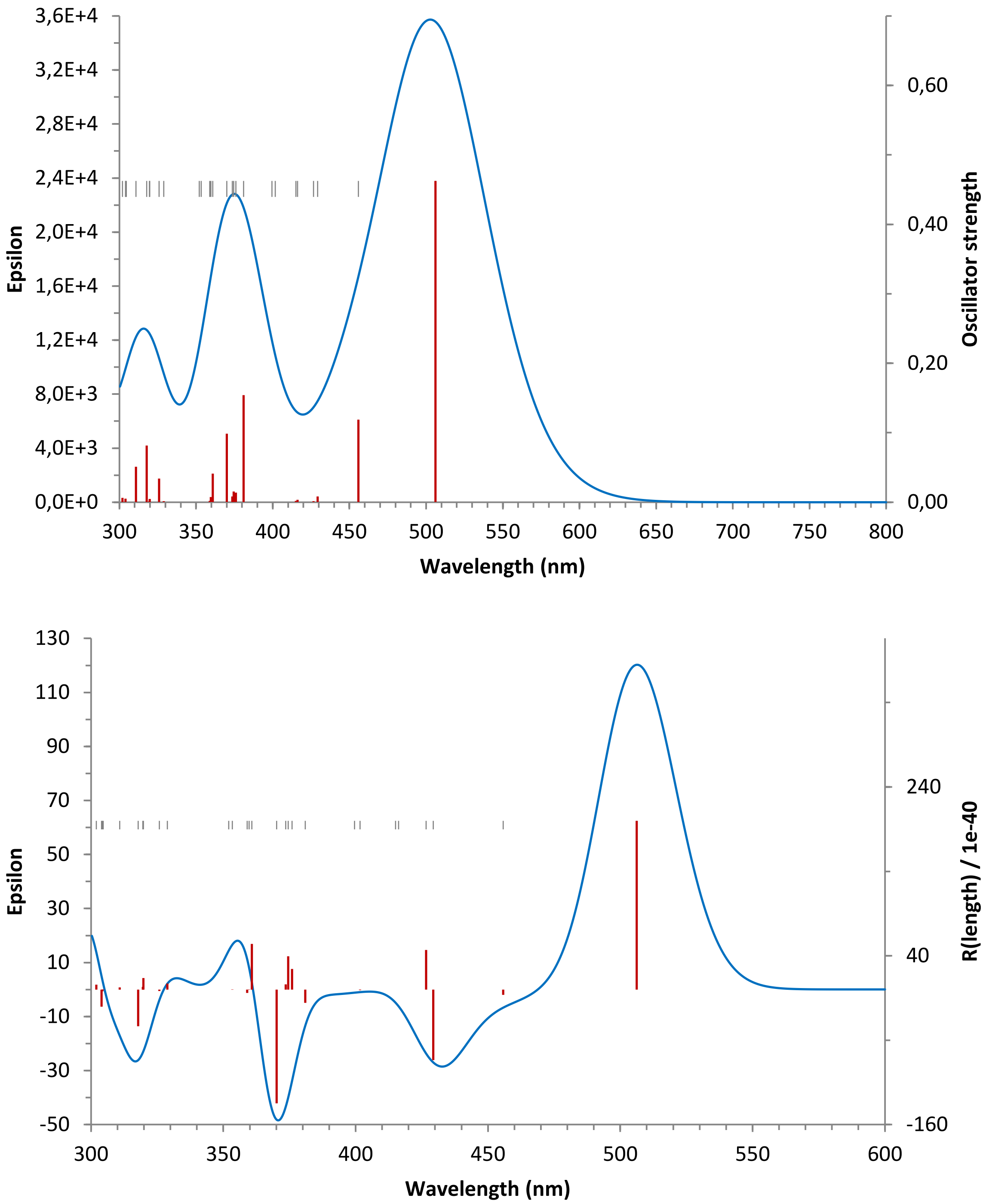

Figure S22. TDA–DFT spectra (top: UV–vis, bottom – CD) for **cNMI3**^{H} (B3LYP/6–31G(d,p)). Individual transitions are shown as red sticks. The blue envelope is a sum of Gaussian profiles with halfwidths of 1500 cm^{-1}.

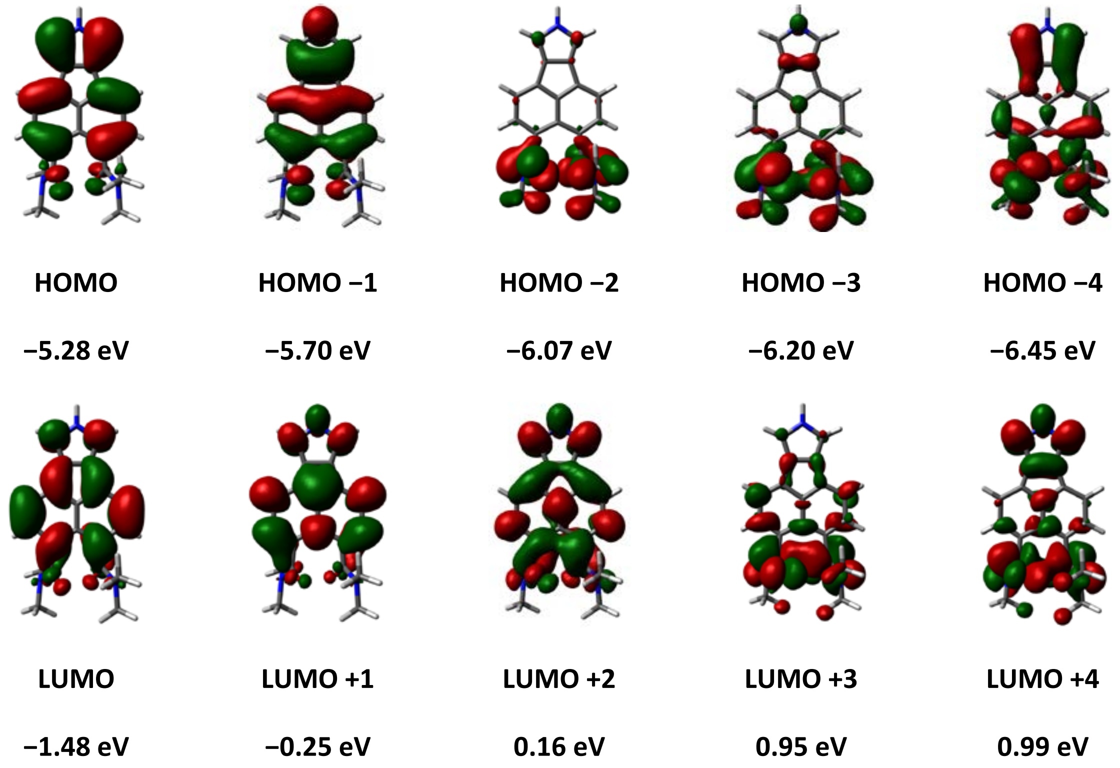

Figure S23. Frontier molecular orbitals (isovalue = 0.02) and orbital energies of **pyrrole NDA**.

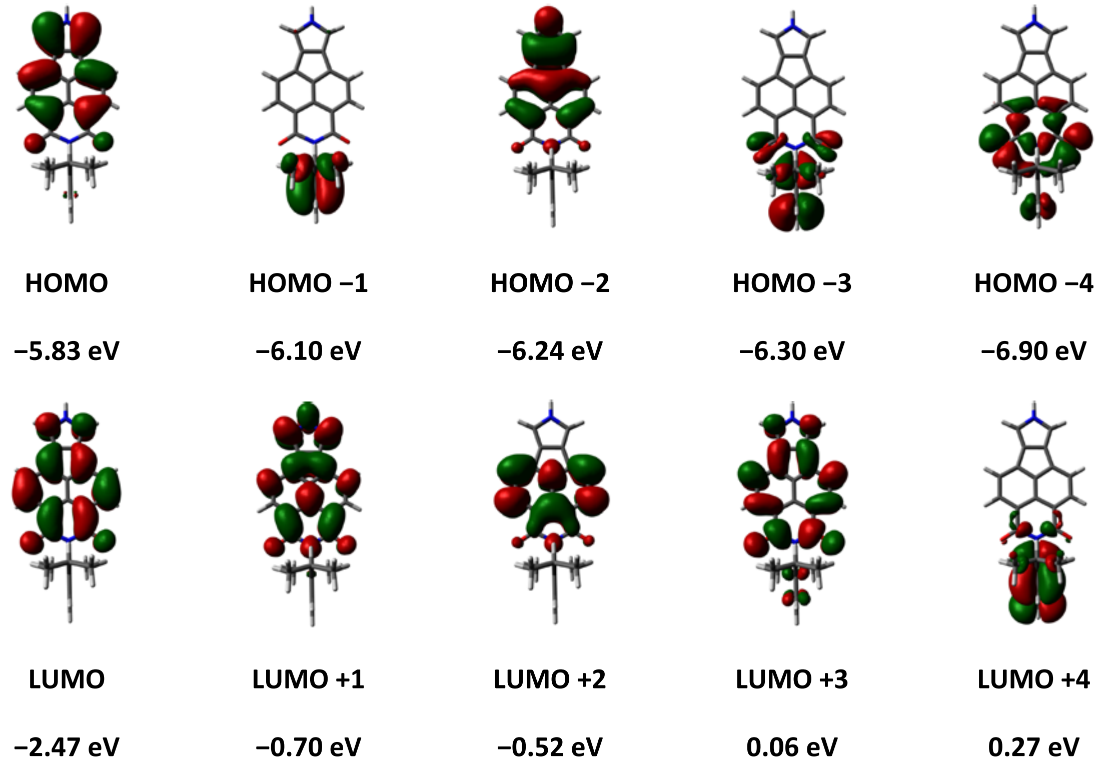

Figure S24. Frontier molecular orbitals (isovalue = 0.02) and orbital energies of **pyrrole NMI**.

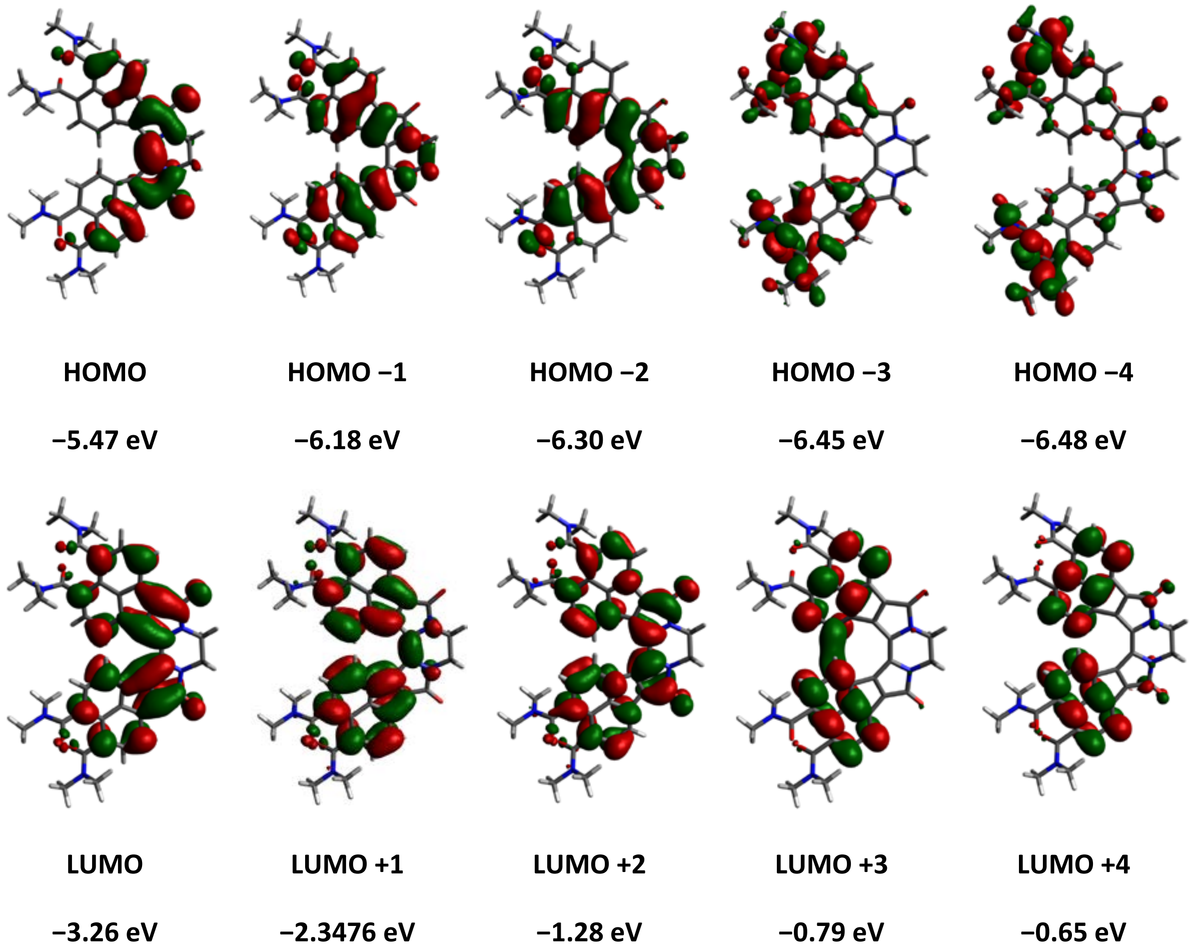

Figure S25. Frontier molecular orbitals (isovalue = 0.02) and orbital energies of **cNDA2^{O}**.

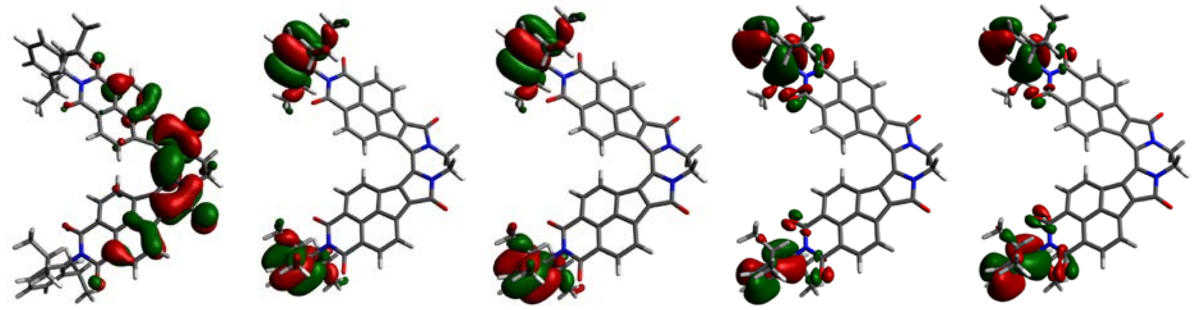

HOMO	HOMO −1	HOMO −2	HOMO −3	HOMO −4
−5.95 eV	−6.40 eV	−6.40 eV	−6.66 eV	−6.66 eV

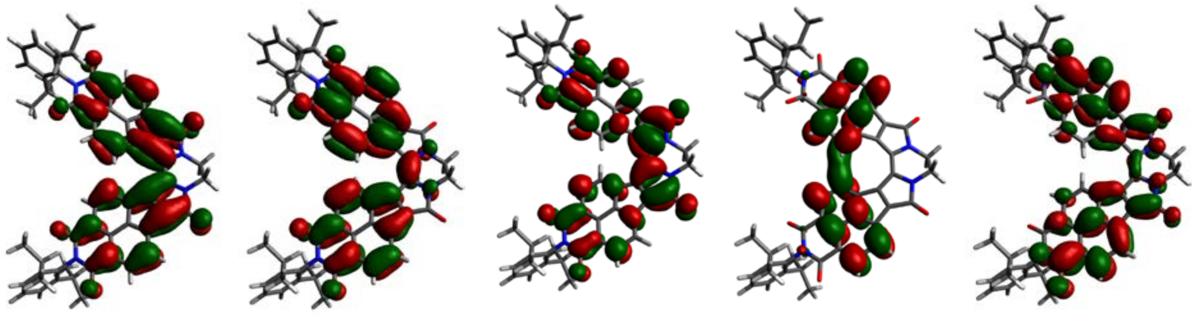

LUMO	LUMO +1	LUMO +2	LUMO +3	LUMO +4
−4.03 eV	−3.28 eV	−2.22 eV	−1.21 eV	−1.10 eV

Figure S26. Frontier molecular orbitals (isovalue = 0.02) and orbital energies of **cNMI2^{O}**.

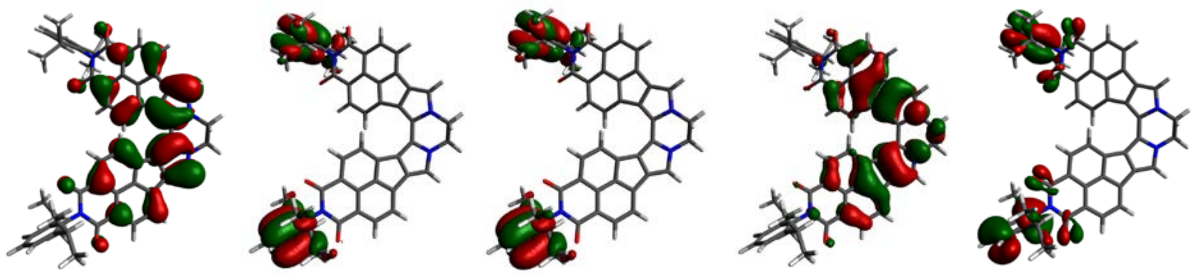

HOMO	HOMO −1	HOMO −2	HOMO −3	HOMO −4
−5.50 eV	−6.12 eV	−6.12 eV	−6.33 eV	−6.35 eV

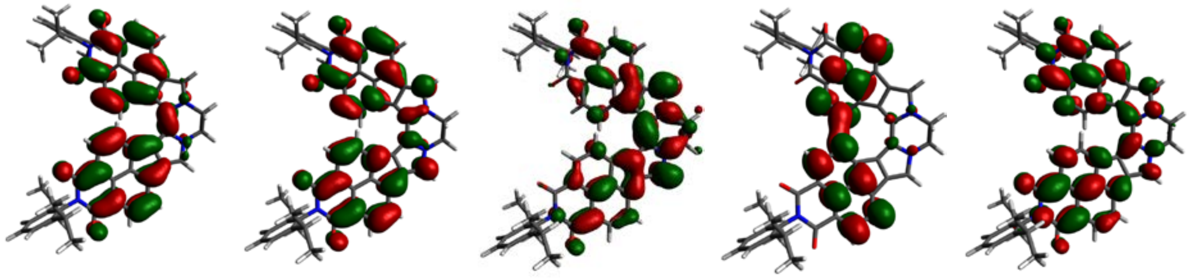

LUMO	LUMO +1	LUMO +2	LUMO +3	LUMO +4
−3.79 eV	−3.76 eV	−3.65 eV	−0.72 eV	−0.66 eV

Figure S27. Frontier molecular orbitals (isovalue = 0.02) and orbital energies of **cNMI2^{H}**.

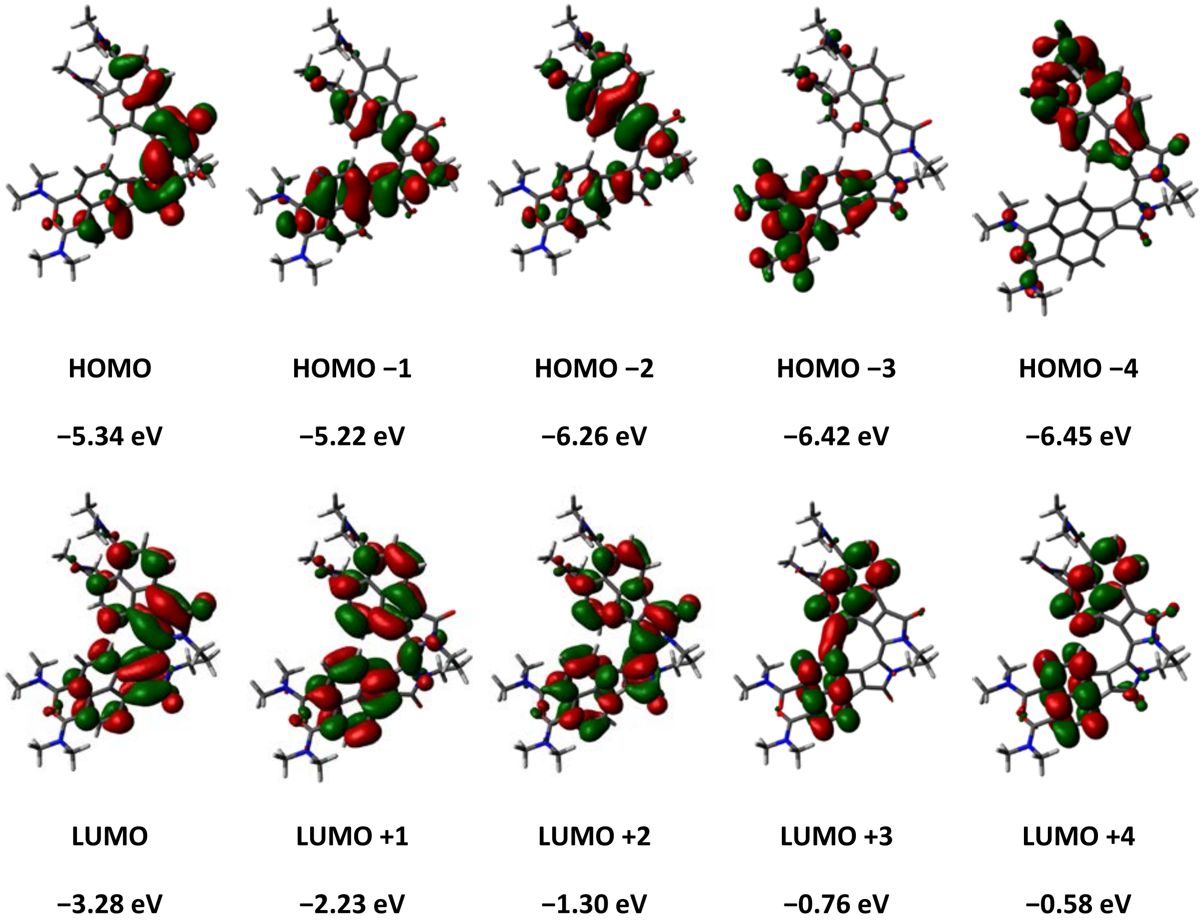

Figure S28. Frontier molecular orbitals (isovalue = 0.02) and orbital energies of **cNDA3^{O}**.

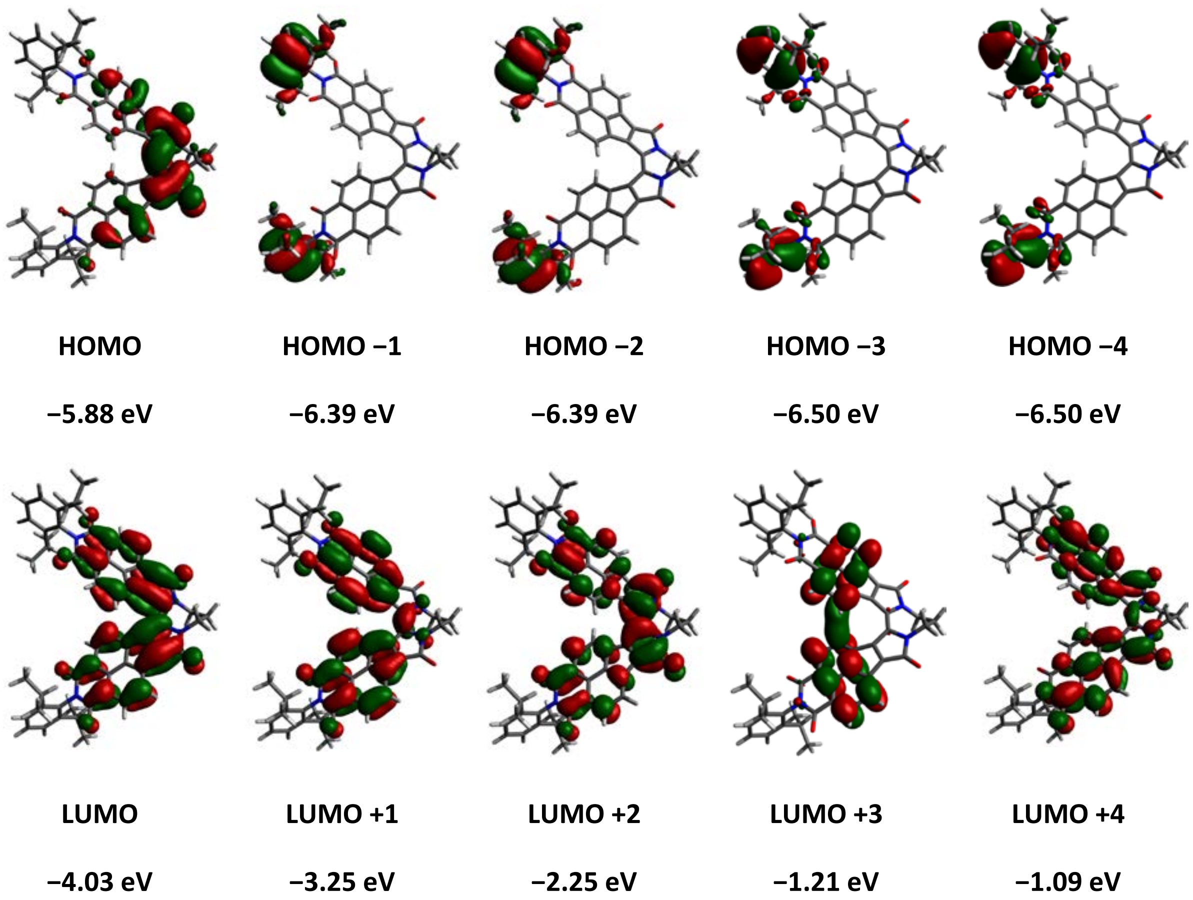

Figure S29. Frontier molecular orbitals (isovalue = 0.02) and orbital energies of **cNMI3°**.

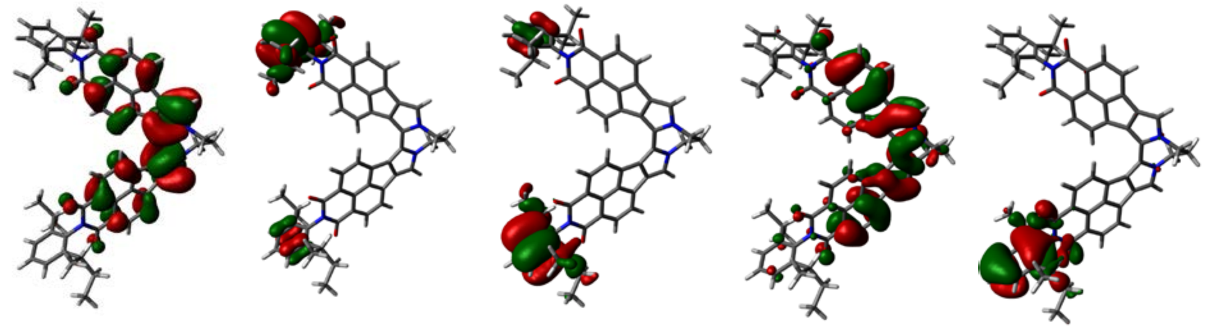

HOMO	HOMO −1	HOMO −2	HOMO −3	HOMO −4
−5.56 eV	−6.11 eV	−6.12 eV	−6.27 eV	−6.32 eV

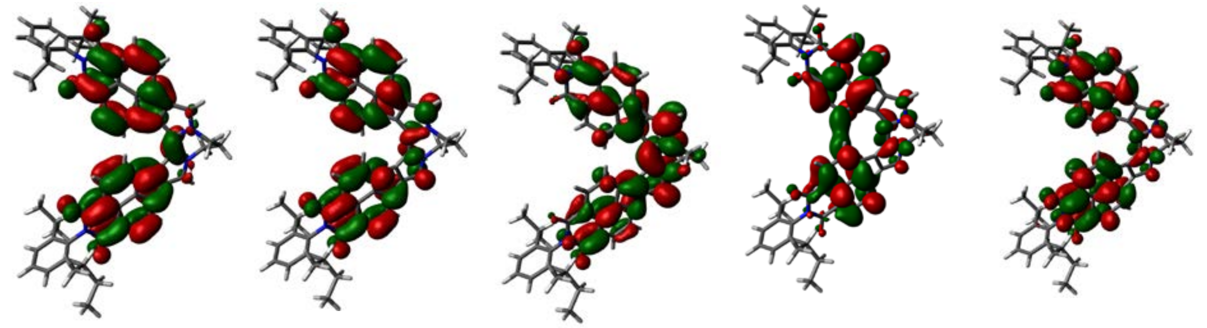

LUMO	LUMO +1	LUMO +2	LUMO +3	LUMO +4
−2.73 eV	−2.51 eV	−1.20 eV	−0.72 eV	−0.68 eV

Figure S30. Frontier molecular orbitals (isovalue = 0.02) and orbital energies of **cNMI3^{H}**.

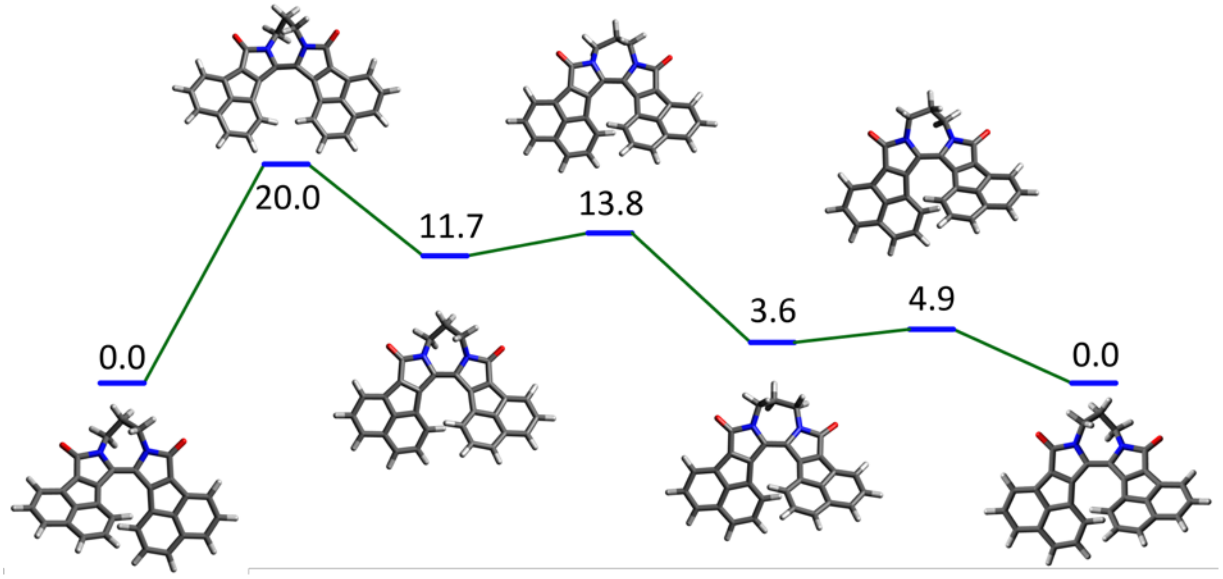

Figure S31. Inversion pathway determined for an analogue of **cNDA3**^{O} using DFT calculations (B3LYP/6-31G(d,p), dimethylaminocarbonyl groups replaced with hydrogens). ΔG^{298} values (kcal/mol) are given relative to lowest energy conformation.

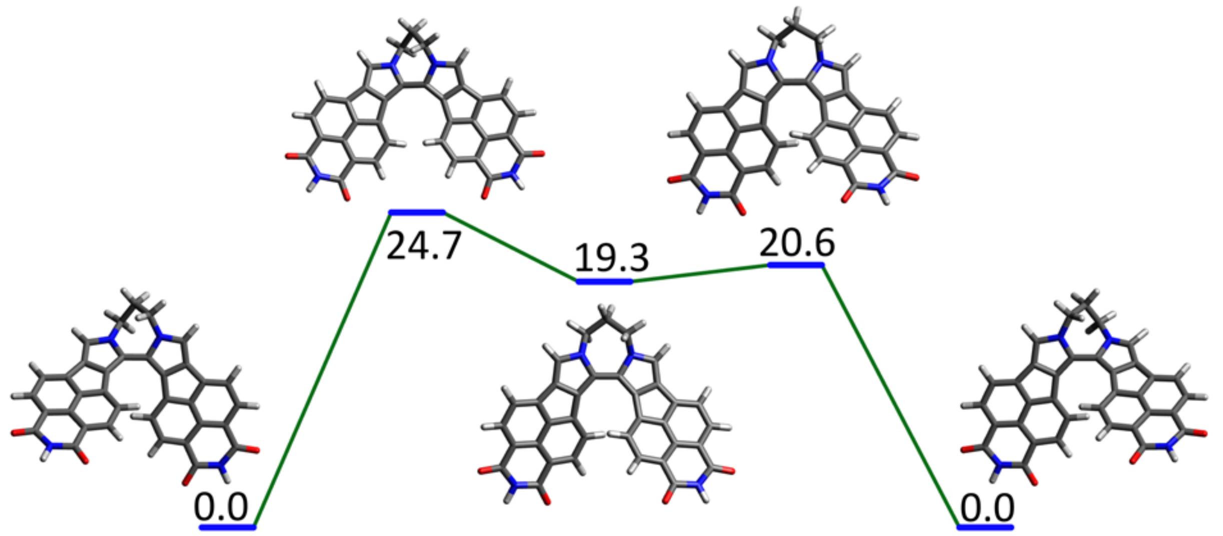

Figure S32. Inversion pathway determined for an analogue of **cNMI3**^{H} using DFT calculations (B3LYP/6-31G(d,p), dipp substituents replaced with hydrogens). ΔG^{298} values (kcal/mol) are given relative to lowest energy conformation.

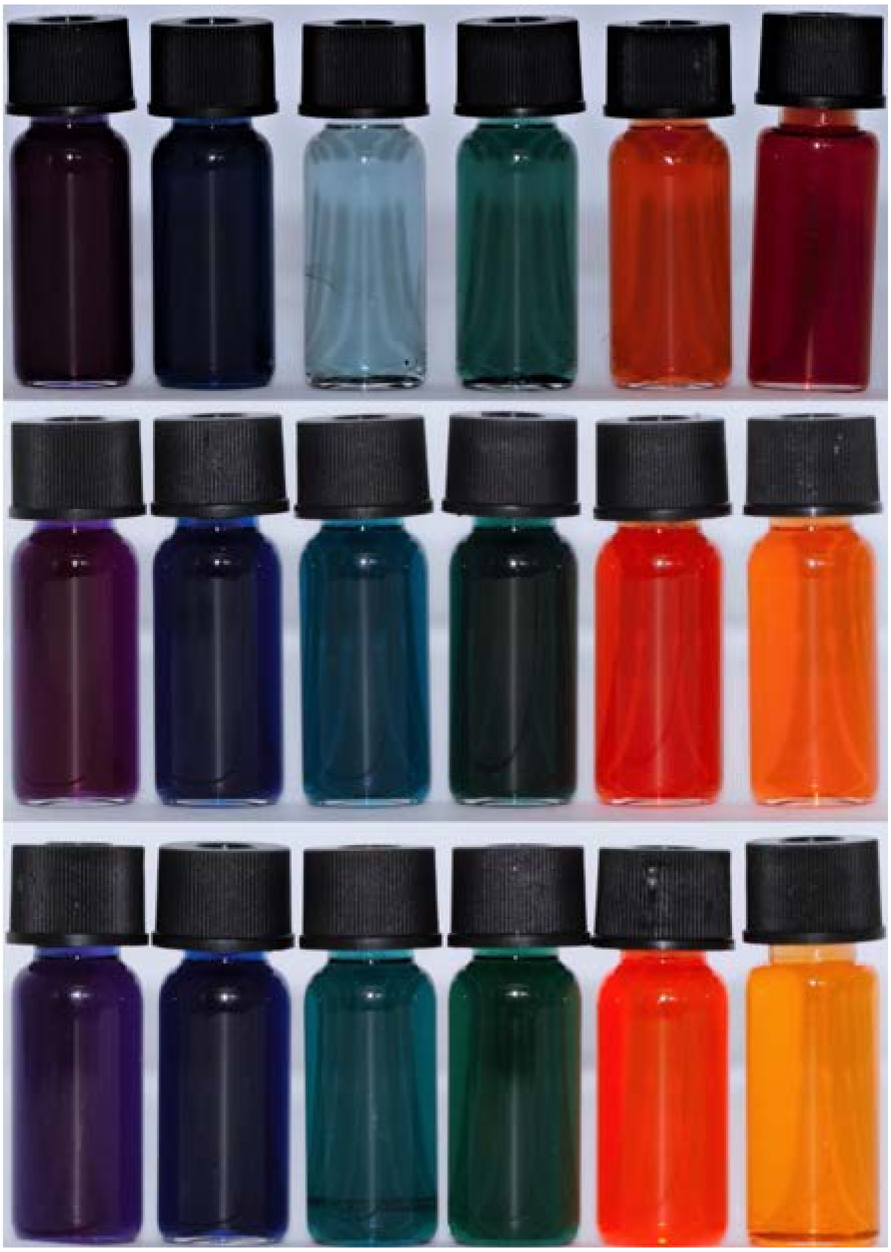

Figure S33. Photos of boomerangs described in this work. From left: **cNDA2^{O}, cNDA3^{O},cNMI2^{O}, cNMI3^{O}, cNMI2^{H}, cNMI3^{H}**. From top: dissolved in acenitrile, dichloromethane, toluene.

Additional tables

Table S1. Calculated[a] values of α and Θ angles for the fused bipyrroles.

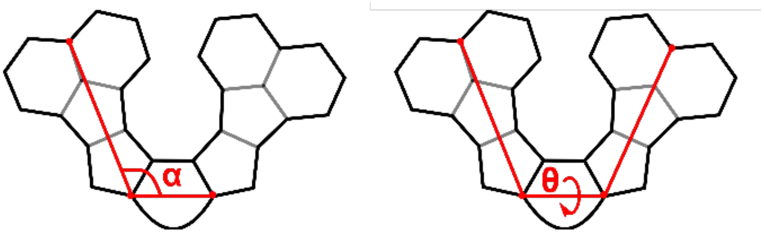

	cNDA2^{O}	cNDA3^{O}	cNMI2^{O}	cNMI3^{O}	cNMI2^{H}	cNMI3^{H}
**α [°]**	119	110	116	110	116	108
**θ [°]**	21	48	35	48	41	58

[a] B3LYP/6-31G(d,p).

Table S2. Electronic transitions calculated for **cNDA2^{O}** using the TDA/B3LYP/6-31G(d,p) level of theory.

No.	Energy (cm^{-1})	λ (nm)	*f*[a]	Major excitations[b]
1	16505	605.9	0.530	HOMO»LUMO (97%)
2	18675	535.5	0.001	H–1»LUMO (97%)
3	19565	511.1	0.002	H–2»LUMO (95%)
4	21327	468.9	0.006	HOMO»L+1 (87%)
5	22567	443.1	0.004	H–4»LUMO (95%)
6	22571	443.1	0.043	H–3»LUMO (86%)
7	23779	420.5	0.026	H–8»LUMO (28%)<br>H–5»LUMO (66%)
8	23860	419.1	0.015	H–7»LUMO (18%)<br>H–6»LUMO (73%)
9	23983	417.0	0.012	H–7»LUMO (74%)<br>H–6»LUMO (13%)
10	24139	414.3	0.083	H–8»LUMO (66%)<br>H–5»LUMO (23%)
11	24768	403.8	0.005	H–12»LUMO (26%)<br>H–11»LUMO (55%)
12	24977	400.4	0.007	H–14»LUMO (67%)<br>H–13»LUMO (21%)
13	25899	386.1	0.074	H–9»LUMO (33%)<br>H–1»L+1 (59%)
14	26457	378.0	0.014	H–9»LUMO (56%)<br>H–1»L+1 (35%)
15	26650	375.2	0.000	H–10»LUMO (86%)
16	27097	369.0	0.009	H–12»LUMO (66%)<br>H–11»LUMO (25%)
17	27207	367.6	0.016	H–14»LUMO (14%)<br>H–13»LUMO (27%)<br>H–2»L+1 (48%)
18	27605	362.3	0.085	H–14»LUMO (12%)<br>H–13»LUMO (38%)<br>H–2»L+1 (34%)
19	28689	348.6	0.019	H–15»LUMO (91%)
20	29198	342.5	0.027	H–16»LUMO (84%)
21	29538	338.6	0.040	H–3»L+1 (92%)
22	29768	335.9	0.010	H–4»L+1 (93%)
23	30842	324.2	0.101	H–8»L+1 (19%)<br>H–5»L+1 (64%)<br>HOMO»L+2 (12%)
24	31068	321.9	0.003	H–8»L+1 (70%)<br>H–5»L+1 (24%)
25	31081	321.7	0.017	H–7»L+1 (93%)
26	31292	319.6	0.027	H–6»L+1 (93%)
27	32055	312.0	0.179	HOMO»L+2 (67%)
28	33049	302.6	0.001	H–17»LUMO (27%)<br>H–9»L+1 (54%)
29	33222	301.0	0.002	H–10»L+1 (35%)<br>HOMO»L+3 (52%)
30	33761	296.2	0.023	H–17»LUMO (28%)<br>H–9»L+1 (20%)<br>H–1»L+2 (30%)<br>HOMO»L+4 (10%)
31	34024	293.9	0.004	H–11»L+1 (87%)
32	34099	293.3	0.062	H–14»L+1 (11%)<br>H–10»L+1 (50%)<br>HOMO»L+3 (28%)
33	34333	291.3	0.002	H–12»L+1 (80%)
34	34353	291.1	0.021	H–14»L+1 (35%)<br>H–13»L+1 (49%)
35	34627	288.8	0.007	H–14»L+1 (48%)<br>H–13»L+1 (37%)
36	34678	288.4	0.001	H–17»LUMO (22%)<br>H–1»L+2 (52%)<br>HOMO»L+4 (18%)
37	34949	286.1	0.002	H–17»LUMO (11%)<br>H–15»L+1 (21%)<br>H–9»L+1 (10%)<br>HOMO»L+4 (44%)
38	35396	282.5	0.002	H–2»L+2 (87%)
39	36184	276.4	0.000	H–15»L+1 (66%)<br>HOMO»L+4 (17%)
40	36380	274.9	0.071	H–16»L+1 (78%)
41	38217	261.7	0.000	H–3»L+2 (91%)
42	38468	260.0	0.005	H–4»L+2 (91%)
43	38783	257.8	0.003	H–1»L+3 (63%)<br>HOMO»L+5 (19%)
44	39047	256.1	0.124	H–1»L+3 (18%)<br>HOMO»L+5 (67%)
45	39178	255.2	0.005	H–2»L+3 (44%)<br>H–1»L+4 (41%)
46	39570	252.7	0.073	H–18»LUMO (11%)<br>HOMO»L+6 (71%)
47	39634	252.3	0.000	H–5»L+2 (91%)
48	39762	251.5	0.006	H–6»L+2 (82%)
49	39943	250.4	0.000	H–8»L+2 (91%)
50	39957	250.3	0.006	H–7»L+2 (84%)
51	40594	246.3	0.055	H–18»LUMO (10%)<br>H–17»L+1 (12%)<br>H–4»L+3 (20%)<br>H–3»L+4 (11%)
52	40776	245.2	0.025	H–18»LUMO (24%)<br>H–11»L+2 (20%)
53	40862	244.7	0.013	H–4»L+4 (20%)<br>H–3»L+3 (54%)
54	40938	244.3	0.012	H–18»LUMO (12%)<br>H–17»L+1 (17%)<br>H–12»L+2 (13%)<br>H–11»L+2 (28%)<br>H–9»L+2 (10%)
55	41091	243.4	0.000	H–14»L+2 (59%)<br>H–13»L+2 (22%)
56	41116	243.2	0.003	H–4»L+3 (19%)<br>H–3»L+4 (10%)<br>H–2»L+3 (29%)<br>H–1»L+4 (35%)
57	41451	241.2	0.010	H–2»L+4 (72%)<br>H–1»L+3 (11%)
58	41504	240.9	0.013	H–18»LUMO (23%)<br>H–17»L+1 (54%)
59	41688	239.9	0.002	H–18»LUMO (10%)<br>H–9»L+2 (38%)
60	41860	238.9	0.003	H–10»L+2 (36%)<br>H–5»L+3 (22%)
61	42295	236.4	0.000	H–8»L+3 (38%)<br>H–7»L+4 (20%)<br>H–5»L+3 (18%)
62	42309	236.4	0.000	H–8»L+4 (20%)<br>H–7»L+3 (48%)<br>H–6»L+3 (11%)
63	42610	234.7	0.011	HOMO»L+7 (76%)
64	42713	234.1	0.020	H–12»L+2 (29%)<br>H–11»L+2 (16%)
65	42801	233.6	0.002	H–13»L+2 (24%)
66	42907	233.1	0.028	H–22»LUMO (19%)<br>H–20»LUMO (47%)
67	42942	232.9	0.001	H–21»LUMO (47%)<br>H–19»LUMO (30%)

68	43023	232.4	0.020	H–20»LUMO (13%)
69	43045	232.3	0.049	H–13»L+2 (10%)<br>H–8»L+3 (10%)
70	43508	229.8	0.017	H–12»L+2 (20%)<br>H–1»L+5 (29%)
71	43648	229.1	0.030	H–21»LUMO (15%)<br>H–19»LUMO (12%)<br>H–13»L+2 (22%)
72	43655	229.1	0.007	H–6»L+3 (17%)<br>H–4»L+3 (27%)<br>H–3»L+4 (49%)
73	43744	228.6	0.002	H–4»L+4 (49%)<br>H–3»L+3 (27%)
74	44165	226.4	0.000	H–21»LUMO (13%)<br>H–19»LUMO (24%)
75	44224	226.1	0.173	H–9»L+2 (12%)<br>H–3»L+4 (10%)
76	44275	225.9	0.000	H–10»L+2 (16%)
77	44456	224.9	0.002	H–15»L+2 (45%)<br>HOMO»L+8 (17%)
78	44525	224.6	0.017	H–22»LUMO (20%)<br>H–15»L+2 (16%)<br>HOMO»L+8 (21%)
79	44613	224.2	0.002	H–16»L+2 (39%)<br>H–13»L+3 (18%)<br>H–12»L+4 (10%)
80	44707	223.7	0.053	H–22»LUMO (15%)<br>H–1»L+5 (28%)
81	44869	222.9	0.053	H–22»LUMO (23%)<br>H–20»LUMO (11%)<br>HOMO»L+8 (29%)
82	45128	221.6	0.001	H–8»L+4 (24%)<br>H–6»L+3 (17%)<br>H–5»L+4 (37%)
83	45171	221.4	0.000	H–8»L+3 (10%)<br>H–7»L+4 (25%)<br>H–6»L+4 (37%)<br>H–5»L+3 (11%)
84	45342	220.5	0.081	H–23»LUMO (22%)<br>H–2»L+5 (30%)
85	45520	219.7	0.018	HOMO»L+9 (58%)
86	45557	219.5	0.002	H–8»L+4 (42%)<br>H–7»L+3 (20%)<br>H–5»L+4 (23%)
87	45603	219.3	0.027	H–8»L+3 (11%)<br>H–7»L+4 (27%)<br>H–6»L+4 (18%)<br>H–2»L+5 (21%)
88	45658	219.0	0.064	H–23»LUMO (56%)<br>H–2»L+5 (18%)
89	46081	217.0	0.002	H–10»L+3 (20%)<br>H–1»L+6 (19%)
90	46087	217.0	0.045	H–15»L+3 (11%)<br>H–10»L+4 (10%)<br>H–9»L+3 (38%)
91	46212	216.4	0.155	H–11»L+3 (27%)<br>H–2»L+6 (11%)
92	46299	216.0	0.187	H–14»L+3 (14%)<br>H–10»L+3 (18%)<br>H–1»L+6 (34%)
93	46428	215.4	0.009	H–14»L+4 (15%)<br>H–12»L+3 (34%)<br>H–11»L+3 (22%)
94	46532	214.9	0.035	H–14»L+3 (33%)<br>H–13»L+3 (13%)<br>H–12»L+4 (12%)<br>H–11»L+4 (15%)
95	46699	214.1	0.273	H–2»L+6 (16%)<br>HOMO»L+10 (17%)
96	46754	213.9	0.011	H–7»L+5 (14%)<br>H–4»L+5 (17%)<br>H–3»L+6 (12%)
97	46858	213.4	0.032	H–8»L+5 (11%)<br>H–7»L+6 (11%)<br>H–3»L+5 (15%)<br>H–2»L+6 (28%)
98	47287	211.5	0.021	H–24»LUMO (46%)<br>H–5»L+5 (21%)
99	47296	211.4	0.052	H–25»LUMO (35%)<br>H–6»L+5 (17%)<br>H–5»L+6 (14%)
100	47358	211.2	0.010	H–24»LUMO (29%)<br>H–6»L+6 (12%)<br>H–5»L+5 (28%)
101	47380	211.1	0.082	H–25»LUMO (51%)
102	47625	210.0	0.158	H–7»L+5 (10%)<br>H–6»L+5 (15%)<br>H–5»L+6 (10%)
103	47642	209.9	0.127	H–26»LUMO (10%)<br>HOMO»L+10 (40%)
104	47843	209.0	0.012	H–10»L+4 (22%)<br>H–9»L+3 (27%)
105	48025	208.2	0.005	H–18»L+1 (13%)<br>H–9»L+4 (21%)<br>H–7»L+5 (10%)
106	48038	208.2	0.018	H–10»L+4 (17%)<br>H–9»L+3 (11%)<br>H–1»L+7 (23%)
107	48122	207.8	0.039	H–16»L+3 (11%)<br>H–10»L+3 (17%)<br>H–9»L+4 (36%)
108	48466	206.3	0.000	H–4»L+5 (32%)<br>H–3»L+6 (31%)
109	48537	206.0	0.105	H–4»L+6 (18%)<br>H–3»L+5 (26%)<br>HOMO»L+10 (10%)
110	48611	205.7	0.001	H–14»L+3 (16%)<br>H–13»L+3 (15%)<br>H–11»L+4 (34%)
111	48701	205.3	0.000	H–14»L+4 (22%)<br>H–13»L+4 (25%)<br>H–11»L+3 (20%)
112	48754	205.1	0.019	H–18»L+1 (56%)
113	48786	205.0	0.037	H–26»LUMO (24%)<br>HOMO»L+10 (15%)
114	48976	204.2	0.017	H–14»L+4 (19%)<br>H–12»L+3 (16%)
115	48990	204.1	0.013	H–14»L+3 (17%)<br>H–13»L+3 (19%)<br>H–12»L+4 (40%)<br>H–11»L+4 (11%)
116	49214	203.2	0.026	HOMO»L+11 (64%)
117	49288	202.9	0.020	H–26»LUMO (12%)<br>H–14»L+4 (14%)<br>H–1»L+7 (14%)<br>H–1»L+9 (10%)
118	49410	202.4	0.013	H–28»LUMO (28%)<br>H–1»L+8 (10%)
119	49526	201.9	0.040	H–28»LUMO (15%)<br>H–17»L+2 (16%)<br>H–2»L+7 (16%)<br>HOMO»L+11 (10%)
120	49541	201.9	0.122	H–15»L+3 (15%)<br>H–13»L+4 (18%)
121	49654	201.4	0.014	H–27»LUMO (64%)

122	49755	201.0	0.005	H–9»L+5 (10%)<br>H–6»L+5 (19%)<br>H–5»L+6 (15%)
123	49786	200.9	0.008	H–28»LUMO (14%)<br>H–11»L+5 (20%)<br>H–9»L+5 (11%)
124	49864	200.5	0.006	H–15»L+3 (18%)<br>H–6»L+6 (20%)<br>H–5»L+5 (20%)
125	49914	200.3	0.016	H–14»L+5 (10%)<br>H–10»L+5 (16%)<br>H–9»L+6 (14%)
126	49956	200.2	0.006	H–6»L+5 (17%)<br>H–5»L+6 (22%)
127	50035	199.9	0.011	H–8»L+5 (26%)<br>H–7»L+6 (22%)
128	50108	199.6	0.005	H–15»L+3 (17%)<br>H–13»L+5 (11%)<br>H–6»L+6 (14%)
129	50119	199.5	0.018	H–8»L+6 (19%)<br>H–7»L+5 (24%)<br>H–1»L+8 (10%)
130	50157	199.4	0.035	H–17»L+2 (13%)<br>H–12»L+5 (10%)
131	50225	199.1	0.027	H–14»L+5 (22%)
132	50391	198.4	0.002	H–17»L+2 (16%)<br>H–16»L+3 (16%)<br>H–15»L+4 (39%)
133	50478	198.1	0.010	H–21»L+1 (22%)<br>H–19»L+1 (44%)
134	50698	197.2	0.051	H–20»L+1 (24%)<br>H–8»L+6 (10%)
135	50819	196.8	0.019	H–16»L+4 (25%)<br>H–7»L+6 (13%)<br>H–3»L+7 (11%)
136	50829	196.7	0.049	H–22»L+1 (10%)<br>H–20»L+1 (17%)<br>H–4»L+7 (12%)
137	50934	196.3	0.139	H–1»L+8 (13%)
138	51060	195.8	0.009	H–21»L+1 (21%)<br>H–16»L+4 (11%)
139	51172	195.4	0.091	H–21»L+1 (13%)<br>H–16»L+4 (24%)<br>H–3»L+7 (19%)
140	51178	195.4	0.298	H–17»L+2 (12%)<br>H–4»L+7 (12%)
141	51322	194.8	0.014	H–33»LUMO (43%)<br>H–30»LUMO (16%)
142	51422	194.5	0.081	H–6»L+7 (14%)<br>H–5»L+8 (14%)<br>H–4»L+7 (13%)
143	51444	194.4	0.005	H–5»L+7 (12%)<br>H–2»L+8 (13%)
144	51507	194.2	0.003	H–17»L+2 (11%)<br>H–4»L+7 (11%)
145	51513	194.1	0.001	H–21»L+1 (14%)<br>H–5»L+7 (18%)<br>H–1»L+9 (10%)
146	51828	192.9	0.003	H–5»L+7 (19%)<br>H–2»L+8 (34%)<br>H–1»L+9 (23%)
147	52066	192.1	0.092	H–6»L+7 (16%)<br>H–2»L+9 (38%)
148	52213	191.5	0.001	H–34»LUMO (14%)<br>H–29»LUMO (14%)<br>H–22»L+1 (15%)
149	52290	191.2	0.052	H–16»L+5 (15%)<br>H–15»L+6 (17%)<br>H–9»L+6 (12%)<br>HOMO»L+12 (11%)
150	52359	191.0	0.001	H–37»LUMO (12%)<br>H–35»LUMO (14%)<br>H–22»L+1 (26%)
151	52412	190.8	0.023	H–23»L+1 (44%)<br>HOMO»L+12 (34%)
152	52461	190.6	0.001	H–15»L+5 (19%)<br>H–9»L+5 (10%)<br>H–6»L+7 (20%)
153	52759	189.5	0.027	H–10»L+5 (12%)<br>H–8»L+7 (16%)
154	52819	189.3	0.000	H–7»L+7 (36%)<br>H–3»L+8 (11%)<br>H–2»L+9 (10%)
155	52915	189.0	0.001	H–31»LUMO (26%)<br>H–29»LUMO (22%)
156	52947	188.9	0.019	H–10»L+5 (23%)<br>H–9»L+6 (21%)<br>H–8»L+7 (25%)
157	53007	188.7	0.002	H–16»L+6 (10%)<br>H–10»L+6 (24%)<br>H–9»L+5 (22%)<br>H–9»L+7 (10%)
158	53118	188.3	0.012	H–30»LUMO (15%)<br>H–23»L+1 (10%)<br>HOMO»L+12 (16%)
159	53243	187.8	0.046	H–37»LUMO (17%)<br>H–34»LUMO (30%)<br>H–29»LUMO (21%)
160	53343	187.5	0.014	H–6»L+7 (10%)<br>H–5»L+8 (22%)
161	53347	187.5	0.006	H–5»L+9 (13%)<br>H–2»L+10 (10%)
162	53388	187.3	0.123	H–13»L+6 (18%)<br>H–12»L+5 (16%)<br>H–11»L+5 (11%)
163	53472	187.0	0.001	H–33»LUMO (16%)<br>H–30»LUMO (24%)<br>H–23»L+1 (11%)<br>H–11»L+6 (10%)
164	53550	186.7	0.000	H–14»L+5 (10%)<br>H–12»L+6 (20%)<br>H–11»L+6 (43%)
165	53618	186.5	0.015	H–31»LUMO (52%)<br>H–29»LUMO (17%)
166	53656	186.4	0.018	H–3»L+9 (12%)
167	53686	186.3	0.003	H–39»LUMO (15%)<br>H–32»LUMO (57%)<br>H–30»LUMO (15%)
168	53686	186.3	0.052	H–14»L+6 (26%)<br>H–13»L+6 (18%)<br>H–1»L+10 (17%)
169	53829	185.8	0.023	H–4»L+9 (22%)<br>H–3»L+8 (15%)<br>H–3»L+10 (13%)
170	53837	185.7	0.024	
171	53906	185.5	0.089	H–14»L+6 (32%)<br>H–1»L+10 (15%)
172	54004	185.2	0.126	H–4»L+8 (14%)<br>H–2»L+10 (27%)
173	54322	184.1	0.035	H–37»LUMO (22%)<br>H–17»L+3 (19%)
174	54348	184.0	0.001	H–39»LUMO (37%)<br>H–36»LUMO (15%)<br>H–32»LUMO (16%)

175	54349	184.0	0.043	H–17»L+3 (15%)<br>H–9»L+7 (14%)
176	54451	183.7	0.003	H–17»L+3 (26%)<br>H–9»L+7 (17%)
177	54465	183.6	0.028	H–12»L+6 (12%)
178	54623	183.1	0.029	H–10»L+7 (21%)<br>H–9»L+8 (12%)<br>H–6»L+8 (12%)
179	54662	182.9	0.003	H–15»L+5 (15%)<br>H–13»L+6 (12%)<br>H–9»L+7 (12%)
180	54867	182.3	0.028	H–4»L+10 (18%)<br>H–3»L+9 (13%)<br>H–3»L+11 (15%)<br>H–1»L+11 (10%)
181	54957	182.0	0.000	H–4»L+9 (22%)<br>H–4»L+11 (13%)<br>H–3»L+10 (18%)
182	54999	181.8	0.025	H–16»L+5 (36%)<br>H–15»L+6 (32%)
183	55063	181.6	0.004	H–40»LUMO (11%)<br>H–38»LUMO (23%)<br>H–24»L+1 (20%)
184	55131	181.4	0.006	H–38»LUMO (18%)<br>H–34»LUMO (14%)<br>H–24»L+1 (12%)
185	55155	181.3	0.001	H–39»LUMO (12%)<br>H–36»LUMO (39%)
186	55212	181.1	0.026	H–2»L+10 (10%)<br>H–1»L+11 (22%)
187	55226	181.1	0.010	H–24»L+1 (26%)<br>H–16»L+6 (20%)
188	55274	180.9	0.005	H–25»L+1 (80%)
189	55326	180.7	0.007	HOMO»L+13 (38%)
190	55406	180.5	0.015	H–8»L+9 (12%)<br>H–7»L+8 (13%)<br>H–5»L+9 (12%)<br>H–1»L+11 (14%)<br>HOMO»L+14 (12%)
191	55418	180.4	0.015	H–16»L+6 (16%)<br>H–6»L+9 (14%)
192	55478	180.3	0.005	H–17»L+4 (30%)
193	55485	180.2	0.004	H–8»L+8 (19%)<br>H–7»L+9 (17%)
194	55665	179.6	0.000	H–17»L+4 (11%)<br>H–5»L+9 (15%)
195	55881	179.0	0.003	H–10»L+7 (10%)<br>HOMO»L+14 (45%)
196	55899	178.9	0.015	H–11»L+7 (19%)
197	55948	178.7	0.000	H–40»LUMO (30%)<br>H–35»LUMO (21%)<br>H–2»L+11 (15%)
198	55995	178.6	0.000	H–12»L+7 (31%)<br>H–11»L+7 (35%)
199	56108	178.2	0.065	H–14»L+7 (10%)<br>H–10»L+7 (19%)<br>H–9»L+8 (12%)<br>HOMO»L+14 (11%)
200	56199	177.9	0.006	H–6»L+9 (24%)

[a] Oscillator strength. [b] Contributions smaller than 10% are not included. H = HOMO, L = LUMO. Orbitals are numbered consecutively regardless of possible degeneracies.

Table S3. Electronic transitions calculated for **cNDA3^{O}** using the TDA/B3LYP/6-31G(d,p) level of theory.

No.	Energy (cm^{-1})	λ (nm)	f[a]	Major excitations[b]
1	15597	641.1	0.491	HOMO»LUMO (98%)
2	18821	531.3	0.007	H–1»LUMO (93%)
3	19202	520.8	0.001	H–2»LUMO (94%)
4	20856	479.5	0.012	HOMO»L+1 (86%)
5	22081	452.9	0.018	H–4»LUMO (18%)<br>H–3»LUMO (75%)
6	22276	448.9	0.029	H–4»LUMO (76%)<br>H–3»LUMO (14%)
7	23337	428.5	0.004	H–7»LUMO (19%)<br>H–6»LUMO (57%)<br>H–5»LUMO (19%)
8	23426	426.9	0.026	H–7»LUMO (26%)<br>H–6»LUMO (34%)<br>H–5»LUMO (26%)
9	23638	423.1	0.011	H–8»LUMO (86%)
10	23793	420.3	0.117	H–7»LUMO (48%)<br>H–5»LUMO (38%)
11	24708	404.7	0.001	H–14»LUMO (28%)<br>H–13»LUMO (31%)<br>H–12»LUMO (23%)<br>H–11»LUMO (14%)
12	24858	402.3	0.003	H–14»LUMO (27%)<br>H–13»LUMO (49%)<br>H–12»LUMO (14%)
13	25742	388.5	0.077	H–9»LUMO (91%)
14	26282	380.5	0.001	H–10»LUMO (87%)
15	26677	374.9	0.010	H–13»LUMO (13%)<br>H–11»LUMO (65%)
16	26956	371.0	0.028	H–14»LUMO (22%)<br>H–12»LUMO (25%)<br>H–2»L+1 (12%)<br>H–1»L+1 (27%)
17	27132	368.6	0.013	H–12»LUMO (13%)<br>H–2»L+1 (62%)
18	27479	363.9	0.107	H–12»LUMO (15%)<br>H–2»L+1 (11%)<br>H–1»L+1 (48%)
19	28264	353.8	0.007	H–15»LUMO (82%)
20	28950	345.4	0.021	H–16»LUMO (86%)
21	29682	336.9	0.047	H–3»L+1 (85%)
22	29938	334.0	0.021	H–4»L+1 (88%)
23	30512	327.7	0.158	H–5»L+1 (16%)<br>HOMO»L+2 (50%)
24	31097	321.6	0.016	H–6»L+1 (90%)
25	31344	319.0	0.025	H–7»L+1 (28%)<br>H–5»L+1 (61%)
26	31377	318.7	0.014	H–8»L+1 (79%)
27	31502	317.4	0.014	H–8»L+1 (13%)<br>H–7»L+1 (55%)<br>H–5»L+1 (14%)
28	32660	306.2	0.037	H–10»L+1 (10%)<br>HOMO»L+3 (80%)
29	33203	301.2	0.005	H–9»L+1 (59%)<br>HOMO»L+4 (19%)
30	33993	294.2	0.019	H–17»LUMO (41%)<br>H–2»L+2 (18%)<br>HOMO»L+4 (16%)
31	34141	292.9	0.060	H–10»L+1 (72%)
32	34283	291.7	0.004	H–11»L+1 (61%)<br>H–1»L+2 (12%)
33	34396	290.7	0.000	H–12»L+1 (44%)<br>H–1»L+2 (40%)
34	34568	289.3	0.002	H–12»L+1 (20%)<br>H–9»L+1 (15%)<br>H–1»L+2 (14%)<br>HOMO»L+4 (31%)
35	34746	287.8	0.005	H–12»L+1 (24%)<br>H–11»L+1 (17%)<br>H–2»L+2 (13%)<br>H–1»L+2 (14%)<br>HOMO»L+4 (15%)
36	34887	286.6	0.006	H–13»L+1 (91%)
37	35075	285.1	0.001	H–17»LUMO (14%)<br>H–14»L+1 (48%)<br>H–2»L+2 (19%)
38	35266	283.6	0.002	H–17»LUMO (17%)<br>H–14»L+1 (35%)<br>H–2»L+2 (30%)
39	36318	275.3	0.002	H–15»L+1 (72%)
40	36729	272.3	0.048	H–16»L+1 (78%)
41	37749	264.9	0.001	H–3»L+2 (93%)
42	37977	263.3	0.003	H–4»L+2 (91%)
43	38402	260.4	0.058	HOMO»L+5 (87%)
44	38741	258.1	0.043	H–18»LUMO (19%)<br>HOMO»L+6 (73%)
45	39068	256.0	0.004	H–6»L+2 (11%)<br>H–5»L+2 (67%)
46	39221	255.0	0.009	H–7»L+2 (10%)<br>H–2»L+4 (10%)<br>H–1»L+3 (46%)
47	39263	254.7	0.004	H–6»L+2 (72%)<br>H–5»L+2 (10%)
48	39315	254.4	0.003	H–7»L+2 (63%)<br>H–1»L+3 (10%)
49	39413	253.7	0.062	H–2»L+3 (63%)<br>H–1»L+4 (17%)
50	39578	252.7	0.000	H–8»L+2 (89%)
51	39772	251.4	0.004	H–18»LUMO (62%)<br>H–7»L+2 (13%)<br>HOMO»L+6 (14%)
52	40639	246.1	0.030	H–9»L+2 (31%)<br>H–4»L+3 (17%)<br>H–3»L+3 (15%)<br>H–3»L+4 (14%)
53	40817	245.0	0.010	H–10»L+2 (12%)<br>H–4»L+3 (22%)<br>H–4»L+4 (12%)<br>H–3»L+3 (25%)
54	41038	243.7	0.002	H–14»L+2 (19%)<br>H–13»L+2 (21%)<br>H–12»L+2 (19%)<br>H–11»L+2 (16%)
55	41125	243.2	0.002	H–14»L+2 (22%)<br>H–13»L+2 (39%)<br>H–12»L+2 (15%)
56	41381	241.7	0.017	H–9»L+2 (25%)<br>H–3»L+3 (14%)<br>H–1»L+4 (17%)
57	41452	241.2	0.005	H–4»L+3 (13%)<br>H–1»L+4 (41%)
58	41630	240.2	0.013	H–10»L+2 (32%)<br>H–2»L+4 (15%)

59	41782	239.3	0.014	H–2»L+4 (50%)<br>H–1»L+4 (12%)
60	41963	238.3	0.004	H–19»LUMO (19%)<br>HOMO»L+7 (68%)
61	42188	237.0	0.025	H–17»L+1 (10%)<br>H–11»L+2 (16%)
62	42299	236.4	0.006	H–19»LUMO (27%)<br>HOMO»L+7 (12%)
63	42331	236.2	0.028	H–17»L+1 (11%)<br>H–7»L+3 (11%)<br>H–6»L+3 (31%)<br>H–6»L+4 (18%)
64	42412	235.8	0.003	H–8»L+3 (54%)<br>H–8»L+4 (17%)
65	42456	235.5	0.043	H–17»L+1 (30%)<br>H–11»L+2 (22%)
66	42590	234.8	0.004	H–21»LUMO (70%)<br>H–17»L+1 (12%)
67	42686	234.3	0.008	H–14»L+2 (21%)<br>H–12»L+2 (26%)
68	42877	233.2	0.017	H–17»L+1 (10%)<br>H–16»L+2 (10%)<br>H–5»L+3 (19%)
69	42965	232.7	0.020	H–17»L+1 (13%)<br>H–7»L+3 (13%)<br>HOMO»L+8 (10%)
70	43306	230.9	0.016	HOMO»L+8 (20%)
71	43354	230.7	0.038	H–20»LUMO (17%)<br>H–12»L+2 (10%)<br>HOMO»L+9 (13%)
72	43588	229.4	0.019	H–3»L+3 (23%)<br>H–3»L+4 (43%)
73	43787	228.4	0.007	H–20»LUMO (38%)
74	43938	227.6	0.025	H–4»L+3 (15%)<br>H–4»L+4 (40%)
75	44043	227.1	0.032	H–22»LUMO (26%)<br>HOMO»L+8 (14%)
76	44091	226.8	0.012	H–15»L+2 (32%)
77	44224	226.1	0.035	H–22»LUMO (19%)<br>H–4»L+4 (12%)
78	44235	226.1	0.053	H–15»L+2 (18%)<br>H–5»L+3 (10%)<br>H–3»L+4 (12%)
79	44406	225.2	0.039	HOMO»L+8 (31%)
80	44499	224.7	0.003	H–16»L+2 (32%)<br>H–12»L+3 (18%)
81	44613	224.2	0.003	H–24»LUMO (26%)<br>H–23»LUMO (48%)
82	45009	222.2	0.010	H–6»L+4 (17%)<br>H–5»L+3 (11%)<br>H–5»L+4 (27%)<br>HOMO»L+9 (15%)
83	45099	221.7	0.019	H–5»L+4 (15%)<br>HOMO»L+9 (35%)
84	45244	221.0	0.019	H–24»LUMO (36%)<br>H–23»LUMO (19%)
85	45399	220.3	0.082	H–24»LUMO (10%)<br>H–6»L+4 (17%)<br>H–1»L+5 (17%)
86	45432	220.1	0.030	H–7»L+4 (46%)
87	45565	219.5	0.030	H–7»L+4 (11%)<br>H–6»L+3 (12%)<br>H–6»L+4 (23%)<br>H–5»L+4 (11%)<br>H–1»L+5 (16%)
88	45668	219.0	0.141	H–2»L+5 (33%)<br>H–1»L+5 (17%)
89	45946	217.6	0.005	H–8»L+3 (18%)<br>H–8»L+4 (64%)
90	46090	217.0	0.150	H–9»L+3 (39%)
91	46203	216.4	0.035	H–11»L+3 (13%)<br>H–10»L+3 (35%)
92	46266	216.1	0.153	HOMO»L+10 (16%)
93	46352	215.7	0.017	H–25»LUMO (13%)<br>H–1»L+6 (31%)<br>HOMO»L+10 (18%)
94	46574	214.7	0.030	H–3»L+5 (17%)<br>H–3»L+6 (13%)<br>H–2»L+6 (10%)
95	46643	214.4	0.036	H–26»LUMO (74%)
96	46683	214.2	0.086	H–4»L+5 (14%)<br>H–4»L+6 (12%)<br>H–1»L+6 (15%)
97	46818	213.6	0.057	H–2»L+6 (26%)
98	46927	213.1	0.020	H–14»L+3 (21%)<br>H–12»L+3 (14%)<br>H–2»L+6 (16%)
99	47053	212.5	0.041	H–13»L+3 (49%)<br>H–13»L+4 (20%)
100	47126	212.2	0.256	H–5»L+5 (13%)<br>H–5»L+6 (11%)
101	47266	211.6	0.030	H–27»LUMO (79%)
102	47289	211.5	0.151	H–14»L+3 (12%)<br>H–7»L+5 (16%)
103	47432	210.8	0.025	H–6»L+5 (10%)<br>H–5»L+5 (22%)
104	47554	210.3	0.107	H–25»LUMO (11%)<br>H–8»L+5 (11%)<br>H–8»L+6 (11%)<br>HOMO»L+10 (10%)
105	47600	210.1	0.030	H–25»LUMO (27%)<br>H–7»L+5 (12%)<br>HOMO»L+10 (16%)
106	47800	209.2	0.026	H–9»L+3 (12%)<br>H–8»L+5 (13%)<br>H–8»L+6 (11%)
107	47859	208.9	0.039	H–18»L+1 (21%)<br>H–9»L+3 (10%)
108	48063	208.1	0.010	H–9»L+4 (21%)
109	48345	206.8	0.016	H–18»L+1 (11%)<br>H–10»L+4 (13%)<br>H–9»L+4 (24%)
110	48391	206.7	0.019	H–18»L+1 (26%)<br>H–1»L+7 (13%)
111	48515	206.1	0.010	H–3»L+5 (32%)<br>H–3»L+6 (34%)
112	48638	205.6	0.011	H–4»L+5 (34%)<br>H–4»L+6 (34%)
113	48755	205.1	0.004	H–11»L+3 (16%)<br>H–11»L+4 (39%)
114	48950	204.3	0.024	H–28»LUMO (33%)<br>HOMO»L+11 (31%)
115	49030	204.0	0.005	H–12»L+3 (14%)<br>H–12»L+4 (41%)
116	49179	203.3	0.009	H–28»LUMO (41%)<br>HOMO»L+11 (24%)
117	49185	203.3	0.005	H–13»L+3 (28%)<br>H–13»L+4 (42%)
118	49307	202.8	0.013	H–29»LUMO (53%)<br>HOMO»L+11 (13%)
119	49510	202.0	0.069	H–14»L+3 (13%)<br>H–14»L+4 (21%)
120	49601	201.6	0.093	H–1»L+7 (19%)<br>H–1»L+9 (11%)

121	49615	201.6	0.055	H–14»L+4 (10%)<br>H–12»L+4 (13%)
122	49745	201.0	0.021	H–14»L+4 (11%)<br>H–2»L+7 (15%)<br>H–2»L+8 (11%)
123	49797	200.8	0.008	H–5»L+6 (16%)
124	49852	200.6	0.008	H–19»L+1 (10%)<br>H–9»L+5 (16%)<br>H–5»L+5 (12%)<br>H–5»L+6 (11%)
125	49911	200.4	0.019	H–11»L+5 (11%)<br>H–10»L+6 (10%)
126	50000	200.0	0.001	H–7»L+5 (31%)<br>H–7»L+6 (20%)<br>H–6»L+6 (11%)
127	50070	199.7	0.034	H–15»L+3 (17%)<br>H–7»L+6 (10%)<br>H–6»L+5 (16%)
128	50139	199.4	0.021	H–17»L+2 (17%)
129	50205	199.2	0.039	H–17»L+2 (10%)
130	50296	198.8	0.046	H–19»L+1 (25%)
131	50335	198.7	0.002	H–19»L+1 (17%)<br>H–8»L+5 (25%)<br>H–8»L+6 (14%)
132	50440	198.3	0.015	H–14»L+4 (17%)<br>H–14»L+5 (19%)
133	50540	197.9	0.027	H–13»L+4 (12%)<br>H–13»L+5 (30%)
134	50582	197.7	0.029	H–21»L+1 (10%)<br>H–16»L+3 (17%)<br>H–15»L+4 (26%)
135	50703	197.2	0.004	H–21»L+1 (38%)
136	50777	196.9	0.070	H–30»LUMO (18%)<br>H–21»L+1 (14%)
137	50834	196.7	0.043	H–30»LUMO (30%)
138	51022	196.0	0.023	H–30»LUMO (19%)<br>H–16»L+4 (18%)
139	51125	195.6	0.091	H–17»L+2 (11%)
140	51209	195.3	0.001	H–20»L+1 (10%)<br>H–3»L+7 (13%)<br>H–1»L+8 (13%)
141	51280	195.0	0.091	H–16»L+4 (17%)<br>HOMO»L+12 (13%)
142	51312	194.9	0.115	H–4»L+7 (18%)
143	51400	194.6	0.039	
144	51558	194.0	0.135	H–7»L+7 (10%)
145	51588	193.8	0.017	H–7»L+7 (15%)<br>H–5»L+7 (10%)
146	51716	193.4	0.019	H–5»L+7 (26%)
147	51725	193.3	0.023	H–16»L+4 (14%)<br>HOMO»L+12 (15%)
148	51887	192.7	0.043	H–31»LUMO (30%)<br>H–17»L+2 (10%)
149	51986	192.4	0.077	H–1»L+8 (18%)<br>H–1»L+9 (22%)
150	52199	191.6	0.009	H–37»LUMO (16%)<br>H–22»L+1 (20%)
151	52225	191.5	0.045	H–38»LUMO (10%)<br>H–36»LUMO (10%)
152	52299	191.2	0.013	H–36»LUMO (12%)<br>H–22»L+1 (14%)<br>H–20»L+1 (14%)
153	52336	191.1	0.073	H–2»L+8 (10%)<br>H–2»L+9 (35%)
154	52491	190.5	0.028	H–15»L+6 (14%)<br>H–9»L+6 (10%)<br>H–5»L+7 (10%)
155	52531	190.4	0.015	H–15»L+5 (12%)<br>H–9»L+5 (14%)<br>H–7»L+7 (13%)
156	52796	189.4	0.001	H–6»L+7 (35%)<br>H–6»L+9 (14%)<br>H–3»L+8 (10%)
157	52890	189.1	0.006	H–10»L+5 (10%)<br>H–8»L+7 (15%)
158	52972	188.8	0.012	H–34»LUMO (14%)<br>H–32»LUMO (42%)
159	53054	188.5	0.018	H–8»L+7 (11%)
160	53175	188.1	0.011	H–33»LUMO (10%)<br>H–10»L+5 (12%)<br>H–10»L+6 (11%)
161	53202	188.0	0.005	H–33»LUMO (25%)<br>H–24»L+1 (13%)<br>H–23»L+1 (14%)
162	53217	187.9	0.003	H–33»LUMO (31%)<br>H–24»L+1 (15%)
163	53305	187.6	0.002	H–40»LUMO (10%)<br>H–34»LUMO (12%)<br>H–32»LUMO (14%)
164	53326	187.5	0.010	H–34»LUMO (13%)
165	53384	187.3	0.029	H–11»L+5 (15%)
166	53438	187.1	0.009	H–5»L+8 (10%)<br>H–5»L+9 (15%)
167	53496	186.9	0.000	H–35»LUMO (25%)<br>H–23»L+1 (30%)
168	53562	186.7	0.010	H–35»LUMO (16%)<br>H–24»L+1 (13%)
169	53611	186.5	0.012	H–12»L+6 (12%)<br>H–3»L+8 (14%)<br>H–3»L+9 (12%)
170	53700	186.2	0.017	H–12»L+5 (21%)<br>H–12»L+6 (11%)<br>H–11»L+6 (10%)
171	53842	185.7	0.069	H–1»L+10 (25%)
172	53918	185.5	0.033	H–4»L+9 (15%)<br>H–1»L+10 (10%)
173	54059	185.0	0.187	H–2»L+10 (32%)
174	54118	184.8	0.010	H–13»L+5 (20%)<br>H–13»L+6 (57%)
175	54257	184.3	0.012	H–14»L+5 (10%)<br>H–14»L+6 (27%)
176	54367	183.9	0.010	H–39»LUMO (16%)<br>H–34»LUMO (10%)<br>H–9»L+7 (18%)
177	54380	183.9	0.053	H–41»LUMO (11%)<br>H–38»LUMO (15%)<br>H–35»LUMO (10%)
178	54443	183.7	0.034	H–41»LUMO (18%)<br>H–39»LUMO (16%)<br>H–35»LUMO (12%)
179	54506	183.5	0.022	H–9»L+7 (18%)
180	54584	183.2	0.006	H–8»L+7 (13%)<br>H–4»L+8 (12%)
181	54647	183.0	0.042	HOMO»L+13 (38%)
182	54726	182.7	0.004	H–10»L+7 (16%)<br>H–9»L+8 (13%)
183	54901	182.1	0.005	H–6»L+8 (20%)<br>H–3»L+9 (11%)<br>H–3»L+10 (20%)
184	54987	181.9	0.003	H–26»L+1 (35%)
185	55053	181.6	0.012	H–26»L+1 (34%)
186	55090	181.5	0.010	H–38»LUMO (12%)<br>H–37»LUMO (12%)<br>H–17»L+3 (17%)
187	55152	181.3	0.000	H–4»L+9 (11%)

188	55193	181.2	0.017	H–40»LUMO (10%)<br>H–36»LUMO (16%)<br>H–17»L+3 (10%)
189	55266	180.9	0.002	
190	55297	180.8	0.008	H–16»L+5 (25%)<br>H–15»L+6 (17%)
191	55401	180.5	0.010	H–6»L+8 (12%)<br>HOMO»L+14 (13%)
192	55442	180.4	0.004	H–27»L+1 (27%)<br>H–17»L+3 (10%)<br>H–16»L+6 (18%)
193	55463	180.3	0.007	H–18»L+2 (19%)<br>HOMO»L+14 (36%)
194	55550	180.0	0.006	H–27»L+1 (23%)<br>H–7»L+8 (12%)<br>H–7»L+9 (14%)
195	55619	179.8	0.014	H–27»L+1 (26%)<br>H–25»L+1 (10%)<br>H–16»L+6 (11%)
196	55718	179.5	0.004	H–18»L+2 (13%)<br>H–8»L+8 (11%)<br>H–8»L+9 (16%)
197	55860	179.0	0.001	H–18»L+2 (27%)<br>HOMO»L+14 (12%)
198	55927	178.8	0.029	H–1»L+11 (10%)
199	55976	178.6	0.023	H–25»L+1 (35%)
200	56179	178.0	0.002	H–11»L+7 (16%)

[a] Oscillator strength. [b] Contributions smaller than 10% are not included. H = HOMO, L = LUMO. Orbitals are numbered consecutively regardless of possible degeneracies.

Table S4. Electronic transitions calculated for **cNMI3^{H}** using the TDA/B3LYP/6-31G(d,p) level of theory.

No.	Energy (cm^{-1})	λ (nm)	*f*[a]	Major excitations[b]
1	19756	506.2	0.463	HOMO»LUMO (99%)
2	21940	455.8	0.119	HOMO»L+1 (91%)
3	23294	429.3	0.008	H–7»LUMO (34%)<br>H–6»L+1 (18%)<br>H–3»LUMO (43%)
4	23441	426.6	0.002	H–7»L+1 (11%)<br>H–6»LUMO (68%)<br>H–3»L+1 (16%)
5	24026	416.2	0.003	H–1»LUMO (82%)<br>H–1»L+1 (13%)
6	24090	415.1	0.002	H–2»LUMO (83%)<br>H–2»L+1 (12%)
7	24898	401.6	0.000	H–4»LUMO (70%)<br>H–4»L+1 (19%)
8	25027	399.6	0.000	H–5»LUMO (71%)<br>H–5»L+1 (21%)
9	26251	380.9	0.154	H–7»LUMO (28%)<br>H–6»L+1 (14%)<br>H–3»LUMO (45%)
10	26597	376.0	0.014	H–1»L+1 (73%)
11	26700	374.5	0.015	H–7»L+1 (13%)<br>H–6»LUMO (15%)<br>H–3»L+1 (26%)<br>H–2»L+1 (28%)
12	26769	373.6	0.008	H–7»L+1 (10%)<br>H–3»L+1 (15%)<br>H–2»L+1 (52%)
13	27019	370.1	0.099	H–7»LUMO (32%)<br>H–6»L+1 (61%)
14	27716	360.8	0.041	H–7»L+1 (53%)<br>H–3»L+1 (29%)
15	27800	359.7	0.008	H–9»LUMO (36%)<br>H–9»L+1 (20%)<br>H–4»LUMO (13%)
16	27857	359.0	0.002	H–8»LUMO (42%)<br>H–8»L+1 (24%)<br>H–5»LUMO (17%)
17	28294	353.4	0.000	H–9»LUMO (13%)<br>H–4»LUMO (10%)<br>H–4»L+1 (68%)
18	28406	352.0	0.000	H–8»LUMO (12%)<br>H–5»LUMO (11%)<br>H–5»L+1 (71%)
19	30408	328.9	0.001	H–12»L+1 (11%)<br>H–10»LUMO (75%)
20	30687	325.9	0.034	H–12»LUMO (57%)<br>H–10»L+1 (22%)
21	31268	319.8	0.005	H–16»LUMO (52%)<br>H–16»L+1 (11%)<br>H–15»L+1 (16%)
22	31289	319.6	0.002	H–16»L+1 (18%)<br>H–15»LUMO (56%)<br>H–15»L+1 (12%)
23	31465	317.8	0.082	H–11»LUMO (32%)<br>HOMO»L+2 (44%)
24	32172	310.8	0.051	H–11»LUMO (50%)<br>HOMO»L+2 (38%)
25	32832	304.6	0.000	H–8»LUMO (30%)<br>H–8»L+1 (63%)
26	32868	304.2	0.000	H–9»LUMO (27%)<br>H–9»L+1 (61%)
27	32894	304.0	0.005	H–13»LUMO (61%)<br>HOMO»L+3 (16%)
28	33115	302.0	0.006	H–12»LUMO (28%)<br>H–10»L+1 (57%)
29	33387	299.5	0.003	H–12»L+1 (44%)<br>H–11»L+1 (20%)<br>H–10»LUMO (16%)
30	33439	299.1	0.009	H–14»LUMO (60%)<br>H–10»L+1 (11%)
31	33604	297.6	0.016	H–12»L+1 (20%)<br>H–11»L+1 (73%)
32	35242	283.8	0.008	H–14»L+1 (22%)<br>H–13»LUMO (20%)<br>HOMO»L+3 (27%)<br>HOMO»L+4 (22%)
33	35355	282.8	0.001	H–14»L+1 (11%)<br>HOMO»L+4 (66%)
34	35463	282.0	0.000	H–14»LUMO (19%)<br>H–13»L+1 (68%)
35	36165	276.5	0.044	H–14»L+1 (32%)<br>H–3»L+2 (12%)<br>HOMO»L+3 (31%)
36	36584	273.3	0.031	H–17»LUMO (67%)
37	36636	273.0	0.036	H–13»L+1 (10%)<br>HOMO»L+5 (73%)
38	36709	272.4	0.008	H–16»LUMO (10%)<br>H–15»LUMO (22%)<br>H–15»L+1 (54%)
39	36765	272.0	0.007	H–16»LUMO (24%)<br>H–16»L+1 (58%)
40	36926	270.8	0.002	H–1»L+2 (81%)
41	37022	270.1	0.019	H–2»L+2 (85%)
42	37322	267.9	0.358	H–17»L+1 (12%)<br>H–6»L+2 (73%)
43	37510	266.6	0.067	H–3»L+2 (70%)
44	38196	261.8	0.005	H–4»L+2 (83%)
45	38344	260.8	0.001	H–5»L+2 (87%)
46	38359	260.7	0.263	H–7»L+2 (64%)
47	38873	257.2	0.011	H–17»L+1 (76%)<br>H–6»L+2 (10%)
48	39991	250.1	0.170	H–7»L+3 (11%)<br>H–3»L+3 (26%)<br>HOMO»L+6 (22%)
49	40079	249.5	0.015	H–7»L+3 (13%)<br>H–3»L+3 (15%)<br>H–3»L+4 (13%)<br>HOMO»L+6 (31%)
50	40169	248.9	0.006	H–7»L+5 (11%)<br>H–6»L+3 (49%)<br>H–3»L+5 (14%)
51	40420	247.4	0.007	H–1»L+3 (18%)<br>H–1»L+4 (47%)<br>H–1»L+5 (21%)
52	40476	247.1	0.004	H–18»LUMO (22%)<br>H–2»L+3 (11%)<br>H–2»L+4 (36%)<br>H–2»L+5 (12%)
53	40504	246.9	0.001	H–18»LUMO (57%)<br>H–18»L+1 (11%)<br>H–2»L+4 (15%)

54	40663	245.9	0.006	H–1»L+3 (61%)<br>H–1»L+4 (16%)
55	40779	245.2	0.008	H–2»L+3 (59%)<br>H–2»L+4 (18%)
56	40904	244.5	0.051	H–3»L+4 (50%)<br>HOMO»L+6 (10%)
57	40944	244.2	0.011	H–6»L+4 (10%)<br>HOMO»L+7 (67%)
58	41386	241.6	0.012	H–19»LUMO (78%)<br>H–19»L+1 (16%)
59	41416	241.5	0.001	H–9»L+2 (10%)<br>H–4»L+4 (44%)<br>H–4»L+5 (14%)
60	41538	240.7	0.000	H–8»L+2 (15%)<br>H–5»L+4 (42%)<br>H–5»L+5 (15%)
61	41592	240.4	0.043	H–7»L+3 (48%)<br>H–7»L+4 (24%)<br>H–3»L+3 (11%)
62	41740	239.6	0.004	H–20»LUMO (11%)<br>H–9»L+2 (25%)<br>H–4»L+3 (28%)<br>H–4»L+5 (10%)
63	41863	238.9	0.000	H–8»L+2 (30%)<br>H–5»L+3 (33%)<br>H–5»L+5 (13%)
64	41935	238.5	0.401	H–7»L+4 (36%)<br>H–3»L+3 (21%)
65	42046	237.8	0.021	H–20»LUMO (44%)<br>H–4»L+3 (28%)
66	42204	236.9	0.003	H–20»LUMO (21%)<br>H–9»L+2 (31%)<br>H–4»L+3 (20%)
67	42249	236.7	0.000	H–8»L+2 (18%)<br>H–5»L+3 (47%)<br>H–5»L+4 (18%)
68	42301	236.4	0.010	H–1»L+5 (66%)
69	42421	235.7	0.009	H–2»L+5 (61%)
70	42492	235.3	0.018	H–21»LUMO (13%)<br>H–4»L+11 (16%)<br>H–2»L+8 (39%)
71	42553	235.0	0.033	H–21»LUMO (40%)<br>H–7»L+5 (10%)<br>H–2»L+8 (10%)
72	42587	234.8	0.006	H–5»L+10 (16%)<br>H–5»L+11 (16%)<br>H–1»L+9 (50%)
73	42609	234.7	0.044	H–7»L+5 (18%)<br>H–6»L+3 (19%)<br>H–3»L+5 (33%)
74	42731	234.0	0.693	H–6»L+4 (59%)
75	42767	233.8	0.002	H–22»LUMO (77%)<br>H–22»L+1 (17%)
76	42770	233.8	0.065	H–7»L+4 (15%)<br>H–6»L+5 (50%)
77	42907	233.1	0.009	H–21»LUMO (10%)<br>H–7»L+5 (23%)<br>H–3»L+5 (26%)
78	43105	232.0	0.005	H–11»L+2 (54%)<br>HOMO»L+10 (22%)<br>HOMO»L+12 (15%)
79	43233	231.3	0.000	H–18»LUMO (16%)<br>H–18»L+1 (83%)
80	43609	229.3	0.026	H–10»L+2 (76%)
81	43773	228.5	0.022	H–11»L+2 (24%)<br>HOMO»L+8 (40%)
82	43842	228.1	0.004	H–23»LUMO (17%)<br>H–4»L+5 (39%)
83	43870	227.9	0.006	H–4»L+5 (16%)<br>HOMO»L+8 (39%)
84	43880	227.9	0.003	H–23»LUMO (59%)<br>H–23»L+1 (10%)
85	43970	227.4	0.000	H–5»L+3 (12%)<br>H–5»L+4 (14%)<br>H–5»L+5 (60%)
86	44064	226.9	0.001	H–19»LUMO (16%)<br>H–19»L+1 (74%)
87	44077	226.9	0.000	HOMO»L+9 (96%)
88	44198	226.3	0.011	H–12»L+2 (49%)<br>HOMO»L+10 (20%)
89	44243	226.0	0.018	H–25»LUMO (56%)
90	44486	224.8	0.001	H–1»L+6 (52%)<br>H–1»L+7 (30%)
91	44496	224.7	0.000	H–24»LUMO (69%)<br>H–24»L+1 (13%)
92	44514	224.6	0.001	H–16»L+2 (11%)<br>H–9»L+6 (11%)<br>H–4»L+6 (14%)<br>H–4»L+7 (10%)
93	44530	224.6	0.001	H–2»L+6 (59%)<br>H–2»L+7 (29%)
94	44571	224.4	0.000	H–15»L+2 (10%)<br>H–5»L+6 (10%)
95	44584	224.3	0.002	HOMO»L+10 (10%)<br>HOMO»L+11 (66%)<br>HOMO»L+12 (10%)
96	44651	224.0	0.002	HOMO»L+10 (16%)<br>HOMO»L+11 (25%)<br>HOMO»L+12 (51%)
97	44796	223.2	0.046	H–27»LUMO (14%)<br>H–26»LUMO (49%)
98	44838	223.0	0.000	H–20»LUMO (16%)<br>H–20»L+1 (72%)
99	45003	222.2	0.001	H–9»L+3 (19%)<br>H–9»L+4 (24%)<br>H–9»L+5 (24%)
100	45015	222.2	0.000	H–8»L+3 (21%)<br>H–8»L+4 (25%)<br>H–8»L+5 (29%)
101	45148	221.5	0.052	H–28»LUMO (57%)
102	45174	221.4	0.000	H–11»L+3 (11%)<br>H–7»L+6 (14%)<br>H–6»L+7 (18%)<br>H–3»L+6 (27%)
103	45242	221.0	0.000	H–21»LUMO (15%)<br>H–21»L+1 (78%)
104	45309	220.7	0.085	H–6»L+6 (37%)<br>H–3»L+7 (28%)
105	45521	219.7	0.000	H–22»LUMO (18%)<br>H–22»L+1 (79%)
106	45530	219.6	0.001	H–27»LUMO (54%)<br>H–27»L+1 (10%)<br>H–26»LUMO (15%)
107	45611	219.2	0.000	H–13»L+2 (55%)<br>H–3»L+6 (11%)
108	45753	218.6	0.008	H–16»L+2 (12%)<br>H–14»L+2 (40%)<br>H–4»L+6 (16%)
109	45842	218.1	0.005	H–14»L+2 (33%)<br>H–4»L+6 (12%)
110	45910	217.8	0.003	H–15»L+2 (22%)<br>H–13»L+2 (10%)<br>H–5»L+6 (14%)<br>H–5»L+7 (10%)

111	45940	217.7	0.002	H–13»L+2 (11%)<br>H–5»L+6 (13%)<br>H–5»L+7 (10%)
112	46055	217.1	0.000	H–15»L+2 (10%)<br>H–9»L+3 (24%)<br>H–8»L+2 (12%)<br>H–8»L+3 (18%)
113	46059	217.1	0.001	H–16»L+2 (14%)<br>H–9»L+2 (10%)<br>H–9»L+3 (12%)<br>H–8»L+3 (27%)
114	46144	216.7	0.001	H–36»LUMO (23%)<br>H–29»LUMO (17%)
115	46228	216.3	0.000	H–37»LUMO (37%)<br>H–37»L+1 (16%)<br>H–33»LUMO (12%)
116	46251	216.2	0.002	H–7»L+7 (14%)<br>H–1»L+7 (22%)
117	46346	215.8	0.000	H–29»LUMO (52%)<br>H–29»L+1 (10%)<br>H–25»L+1 (11%)
118	46371	215.7	0.000	H–23»L+1 (53%)
119	46424	215.4	0.000	H–25»L+1 (30%)<br>H–1»L+7 (12%)
120	46488	215.1	0.000	H–2»L+6 (10%)<br>H–2»L+7 (38%)<br>H–1»L+6 (17%)
121	46649	214.4	0.004	H–7»L+7 (14%)<br>H–3»L+7 (29%)<br>H–2»L+6 (13%)<br>H–2»L+7 (11%)
122	46863	213.4	0.026	H–10»L+4 (13%)<br>H–7»L+7 (16%)<br>H–6»L+6 (20%)<br>H–3»L+7 (10%)
123	46887	213.3	0.005	H–30»LUMO (78%)<br>H–30»L+1 (14%)
124	46952	213.0	0.021	H–7»L+6 (17%)<br>H–6»L+7 (30%)
125	47108	212.3	0.000	H–24»LUMO (14%)<br>H–24»L+1 (79%)
126	47208	211.8	0.005	H–26»L+1 (22%)<br>H–11»L+3 (26%)
127	47254	211.6	0.020	H–26»L+1 (14%)<br>H–11»L+3 (29%)<br>H–11»L+4 (18%)
128	47377	211.1	0.011	H–10»L+3 (55%)<br>H–10»L+4 (12%)
129	47465	210.7	0.091	H–10»L+4 (22%)<br>H–7»L+7 (16%)<br>H–6»L+6 (10%)
130	47534	210.4	0.089	H–17»L+2 (10%)<br>H–12»L+3 (18%)<br>HOMO»L+13 (12%)
131	47565	210.2	0.052	H–17»L+2 (26%)<br>HOMO»L+13 (40%)
132	47702	209.6	0.002	H–9»L+7 (12%)<br>H–4»L+6 (13%)
133	47733	209.5	0.017	H–28»L+1 (12%)<br>H–8»L+7 (10%)
134	47752	209.4	0.007	H–28»L+1 (21%)
135	47788	209.3	0.024	H–28»L+1 (17%)<br>H–12»L+3 (18%)<br>H–12»L+4 (15%)
136	47914	208.7	0.027	H–4»L+6 (10%)<br>H–4»L+7 (20%)<br>H–4»L+8 (12%)
137	47967	208.5	0.019	H–4»L+6 (12%)<br>H–4»L+7 (23%)<br>H–4»L+8 (11%)
138	48021	208.2	0.056	H–5»L+9 (27%)<br>H–1»L+10 (24%)<br>H–1»L+11 (15%)
139	48057	208.1	0.000	H–5»L+6 (30%)<br>H–5»L+7 (45%)
140	48183	207.5	0.000	H–27»LUMO (11%)<br>H–27»L+1 (61%)<br>H–26»L+1 (22%)
141	48249	207.3	0.003	H–11»L+3 (12%)<br>H–11»L+4 (47%)
142	48310	207.0	0.000	H–31»LUMO (78%)<br>H–31»L+1 (16%)
143	48509	206.1	0.009	H–3»L+10 (18%)<br>H–3»L+12 (12%)<br>H–1»L+12 (12%)
144	48698	205.3	0.137	H–13»L+3 (17%)<br>H–12»L+5 (20%)<br>H–11»L+5 (10%)<br>H–10»L+3 (17%)<br>H–10»L+4 (11%)
145	48719	205.3	0.039	H–11»L+5 (10%)<br>H–8»L+4 (16%)<br>H–8»L+5 (37%)
146	48751	205.1	0.005	H–9»L+4 (14%)<br>H–9»L+5 (38%)
147	48805	204.9	0.098	H–11»L+5 (46%)
148	48912	204.4	0.008	H–2»L+10 (15%)<br>H–2»L+12 (11%)<br>H–1»L+10 (20%)<br>H–1»L+12 (18%)
149	48938	204.3	0.005	H–29»LUMO (14%)<br>H–29»L+1 (79%)
150	48994	204.1	0.001	H–12»L+4 (11%)<br>H–10»L+5 (21%)
151	49039	203.9	0.002	H–16»L+3 (11%)<br>H–16»L+5 (12%)<br>H–15»L+3 (14%)
152	49051	203.9	0.002	H–2»L+10 (22%)<br>H–2»L+12 (14%)
153	49087	203.7	0.005	H–16»L+3 (13%)<br>H–12»L+4 (15%)<br>H–10»L+5 (25%)
154	49121	203.6	0.001	H–6»L+8 (11%)<br>H–3»L+8 (44%)<br>H–1»L+8 (30%)
155	49283	202.9	0.020	H–32»LUMO (14%)<br>H–7»L+10 (13%)<br>H–7»L+12 (12%)<br>H–3»L+10 (16%)
156	49339	202.7	0.002	H–6»L+10 (22%)<br>H–6»L+12 (17%)<br>H–1»L+8 (15%)
157	49366	202.6	0.000	H–6»L+9 (12%)<br>H–3»L+9 (59%)<br>H–2»L+9 (19%)
158	49390	202.5	0.001	H–3»L+8 (18%)<br>H–1»L+8 (38%)
159	49469	202.1	0.000	H–30»LUMO (15%)<br>H–30»L+1 (82%)
160	49642	201.4	0.034	H–13»L+3 (31%)<br>H–13»L+4 (35%)
161	49677	201.3	0.000	H–3»L+9 (14%)<br>H–2»L+9 (68%)
162	49683	201.3	0.017	H–39»LUMO (15%)<br>H–34»LUMO (12%)

163	49700	201.2	0.026	H–32»LUMO (18%)<br>H–14»L+4 (24%)
164	49736	201.1	0.017	H–33»LUMO (29%)
165	49818	200.7	0.010	H–14»L+3 (11%)
166	49855	200.6	0.031	H–34»LUMO (19%)<br>H–14»L+3 (15%)
167	49909	200.4	0.006	H–34»LUMO (32%)
168	49929	200.3	0.006	H–3»L+11 (33%)<br>H–1»L+11 (25%)
169	49961	200.2	0.032	H–14»L+3 (11%)<br>H–3»L+12 (10%)<br>H–1»L+12 (12%)
170	49968	200.1	0.000	H–7»L+8 (51%)<br>H–6»L+8 (15%)
171	50109	199.6	0.021	H–17»L+2 (41%)<br>HOMO»L+13 (28%)
172	50117	199.5	0.001	H–37»LUMO (10%)<br>H–35»LUMO (61%)<br>H–35»L+1 (10%)
173	50164	199.3	0.001	H–3»L+11 (32%)<br>H–2»L+10 (11%)<br>H–2»L+11 (10%)<br>H–2»L+12 (18%)
174	50180	199.3	0.008	H–6»L+11 (10%)<br>H–3»L+10 (11%)<br>H–3»L+12 (26%)<br>H–2»L+11 (14%)<br>H–1»L+12 (10%)
175	50194	199.2	0.005	H–39»LUMO (13%)<br>H–15»L+4 (11%)<br>H–7»L+9 (19%)
176	50215	199.1	0.001	H–7»L+9 (36%)<br>H–6»L+9 (15%)
177	50317	198.7	0.003	H–41»LUMO (15%)<br>H–39»L+1 (10%)<br>H–16»L+3 (13%)<br>H–16»L+4 (17%)
178	50347	198.6	0.003	H–4»L+10 (39%)<br>H–4»L+12 (26%)
179	50442	198.2	0.003	H–5»L+10 (35%)<br>H–5»L+12 (27%)
180	50481	198.1	0.175	H–13»L+4 (15%)<br>H–12»L+5 (14%)<br>H–6»L+10 (10%)
181	50699	197.2	0.291	H–33»L+1 (12%)<br>H–9»L+8 (14%)<br>H–7»L+10 (17%)
182	50752	197.0	0.005	H–32»L+1 (33%)<br>H–7»L+11 (20%)
183	50827	196.7	0.037	H–32»L+1 (14%)<br>H–7»L+12 (15%)<br>H–6»L+11 (10%)
184	50850	196.7	0.010	H–7»L+11 (36%)<br>H–7»L+12 (11%)<br>H–6»L+12 (10%)
185	50868	196.6	0.002	H–7»L+8 (27%)<br>H–6»L+8 (48%)<br>H–3»L+8 (12%)
186	50919	196.4	0.062	H–33»L+1 (10%)<br>H–9»L+8 (50%)
187	51019	196.0	0.000	H–31»LUMO (16%)<br>H–31»L+1 (82%)
188	51065	195.8	0.015	H–8»L+9 (61%)
189	51096	195.7	0.000	H–7»L+9 (28%)<br>H–6»L+9 (34%)<br>H–4»L+9 (25%)<br>H–3»L+9 (12%)
190	51137	195.6	0.000	H–5»L+8 (94%)
191	51166	195.4	0.001	H–33»L+1 (10%)<br>H–14»L+4 (18%)<br>H–13»L+5 (48%)
192	51244	195.1	0.026	H–14»L+5 (52%)
193	51258	195.1	0.000	H–6»L+9 (17%)<br>H–4»L+9 (73%)
194	51340	194.8	0.004	HOMO»L+14 (58%)
195	51346	194.8	0.013	HOMO»L+14 (28%)
196	51399	194.6	0.012	H–15»L+6 (11%)<br>H–15»L+7 (13%)
197	51409	194.5	0.117	H–36»LUMO (17%)<br>H–33»L+1 (12%)
198	51440	194.4	0.013	H–38»LUMO (13%)<br>H–13»L+8 (12%)<br>H–12»L+8 (20%)<br>H–10»L+8 (14%)
199	51503	194.2	0.039	H–38»LUMO (16%)
200	51534	194.0	0.010	H–7»L+11 (12%)<br>H–6»L+10 (10%)<br>H–6»L+11 (11%)

[a] Oscillator strength. [b] Contributions smaller than 10% are not included. H = HOMO, L = LUMO. Orbitals are numbered consecutively regardless of possible degeneracies.

NMR spectra

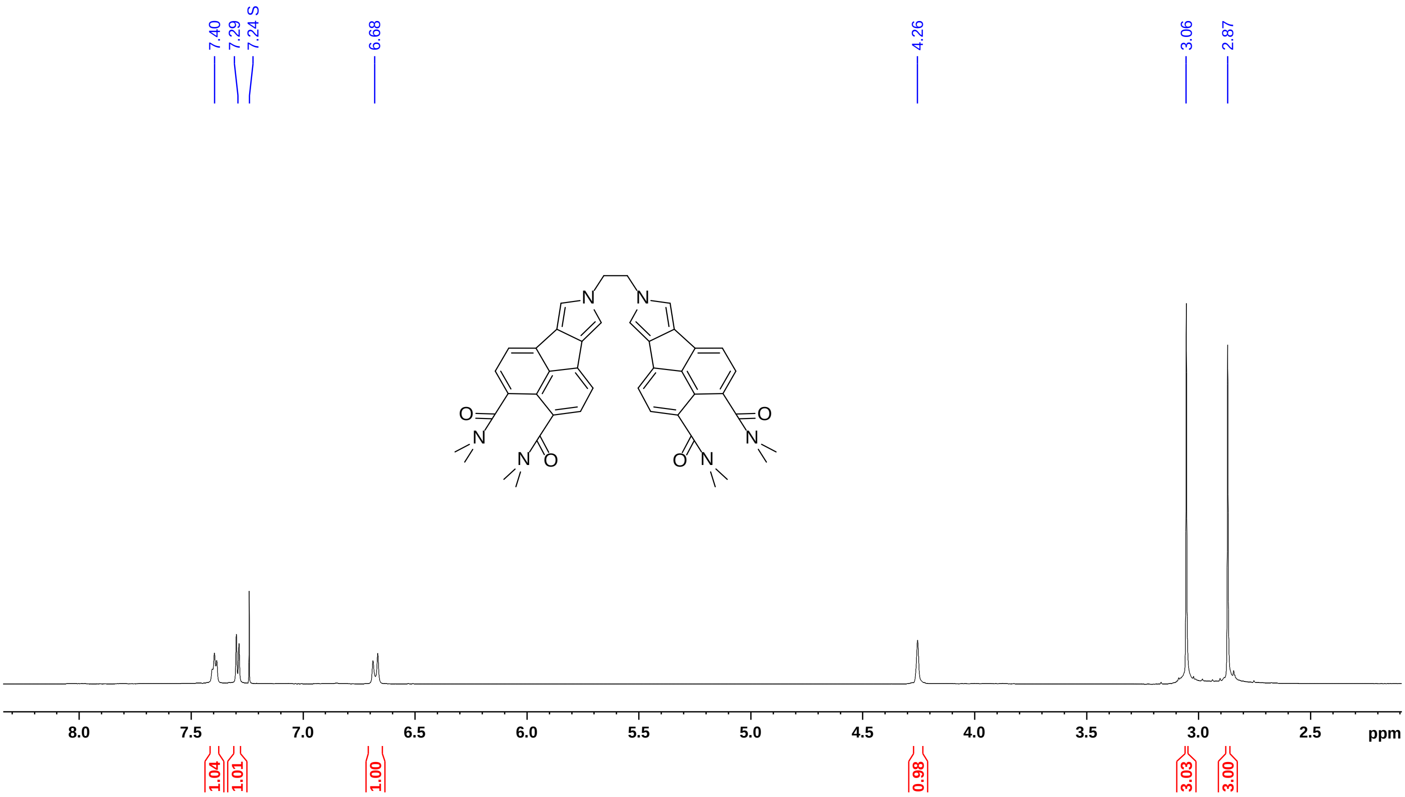

Figure S34. ^{1}H NMR spectrum of **NDA2**^{H} (600 MHz, chloroform-*d* , 300 K).

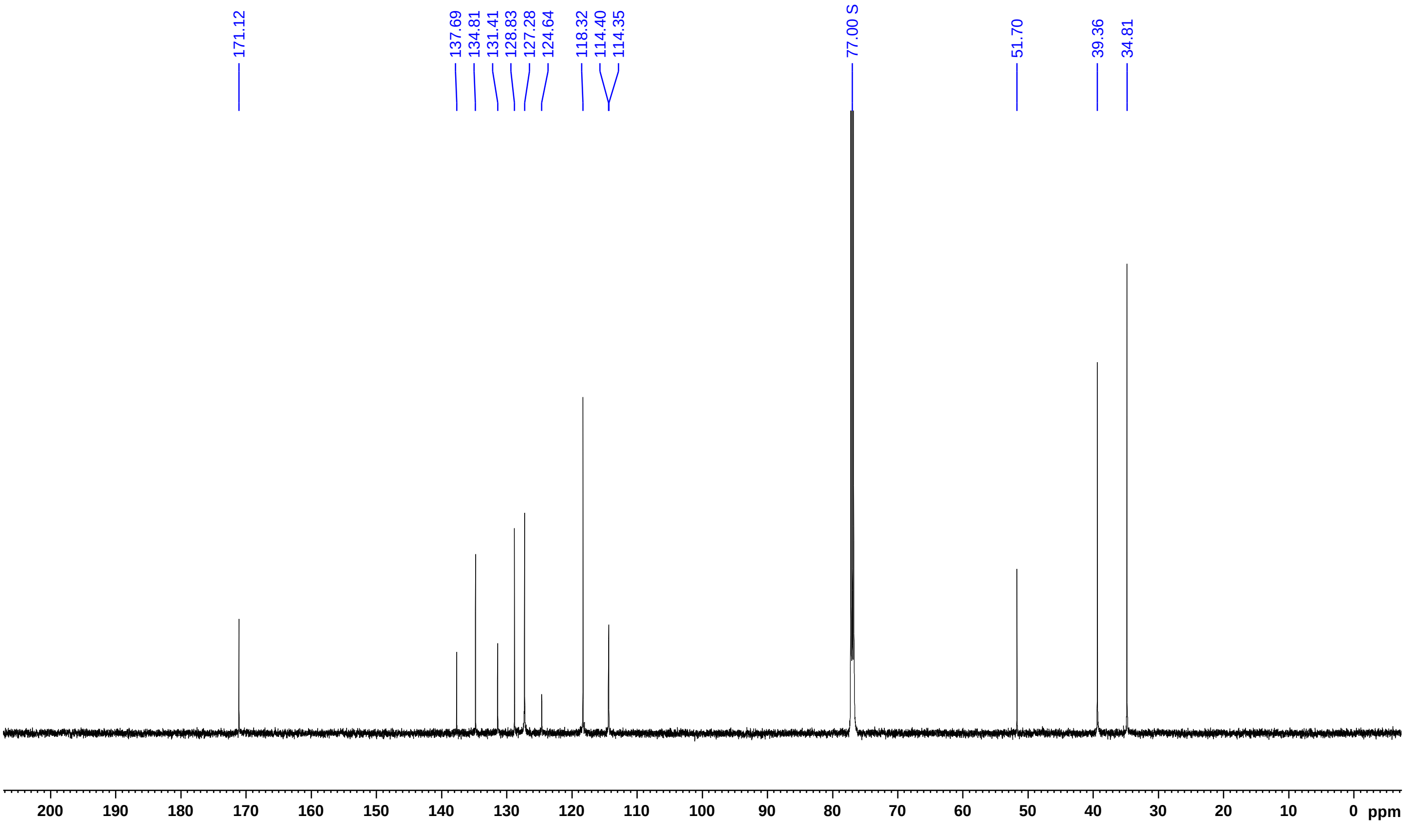

Figure S35. ^{13}C NMR spectrum of **NDA2**^{H} (151 MHz, chloroform-*d* , 300 K).

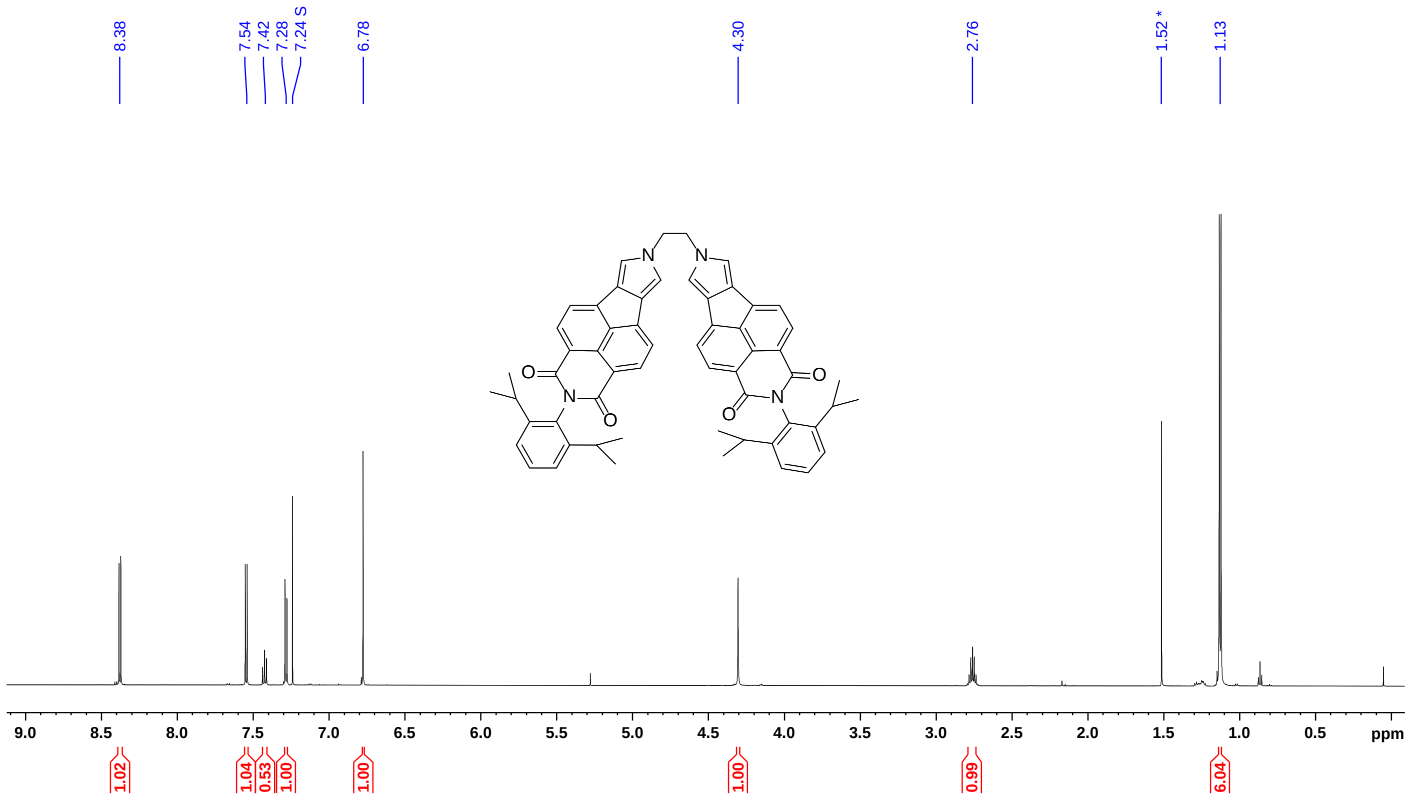

Figure S36. ^{1}H NMR spectrum of **NMI2**^{H} (600 MHz, chloroform-*d* , 300 K).

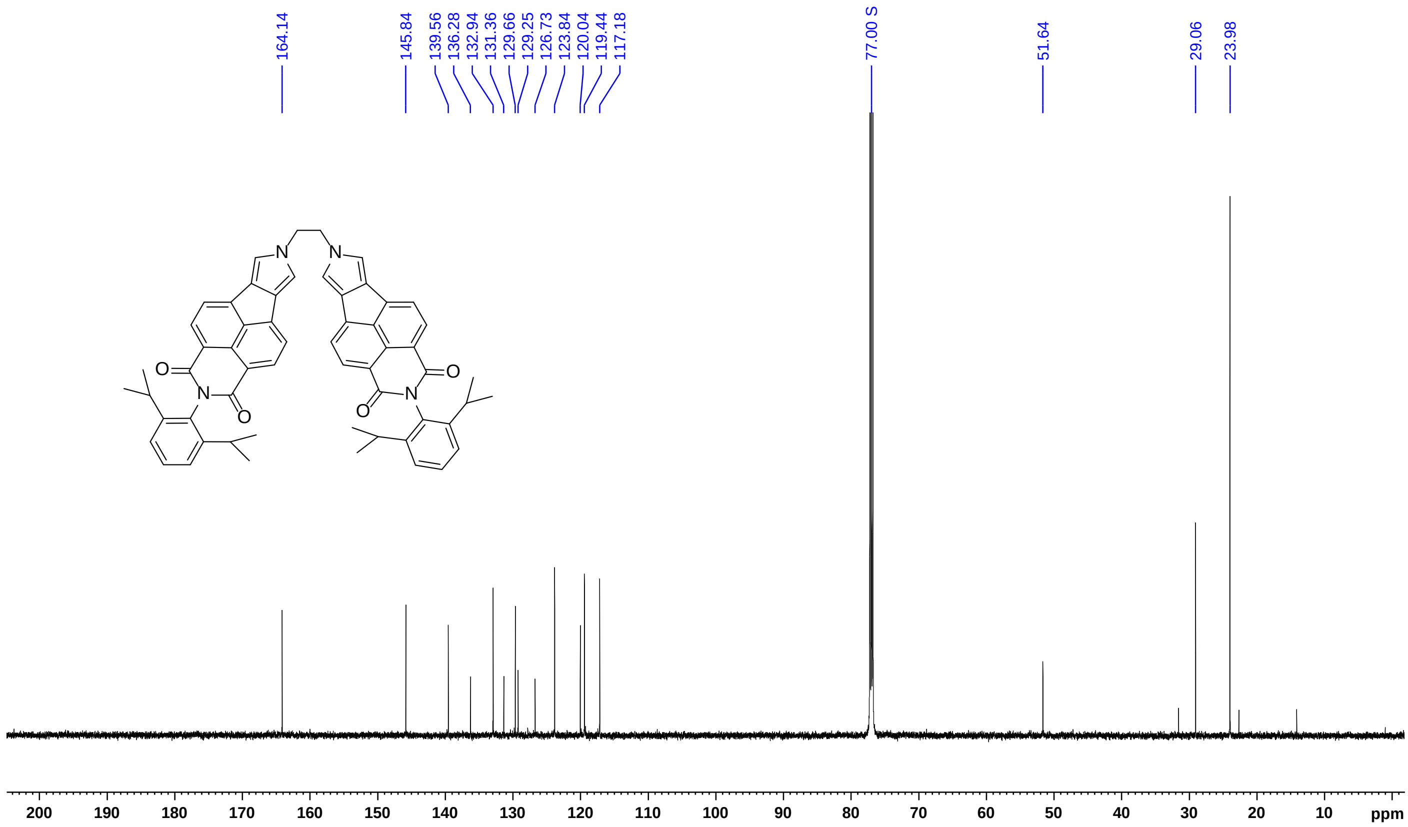

Figure S37. ^{13}C NMR spectrum of **NMI2**^{H} (151 MHz, chloroform-*d* , 300 K).

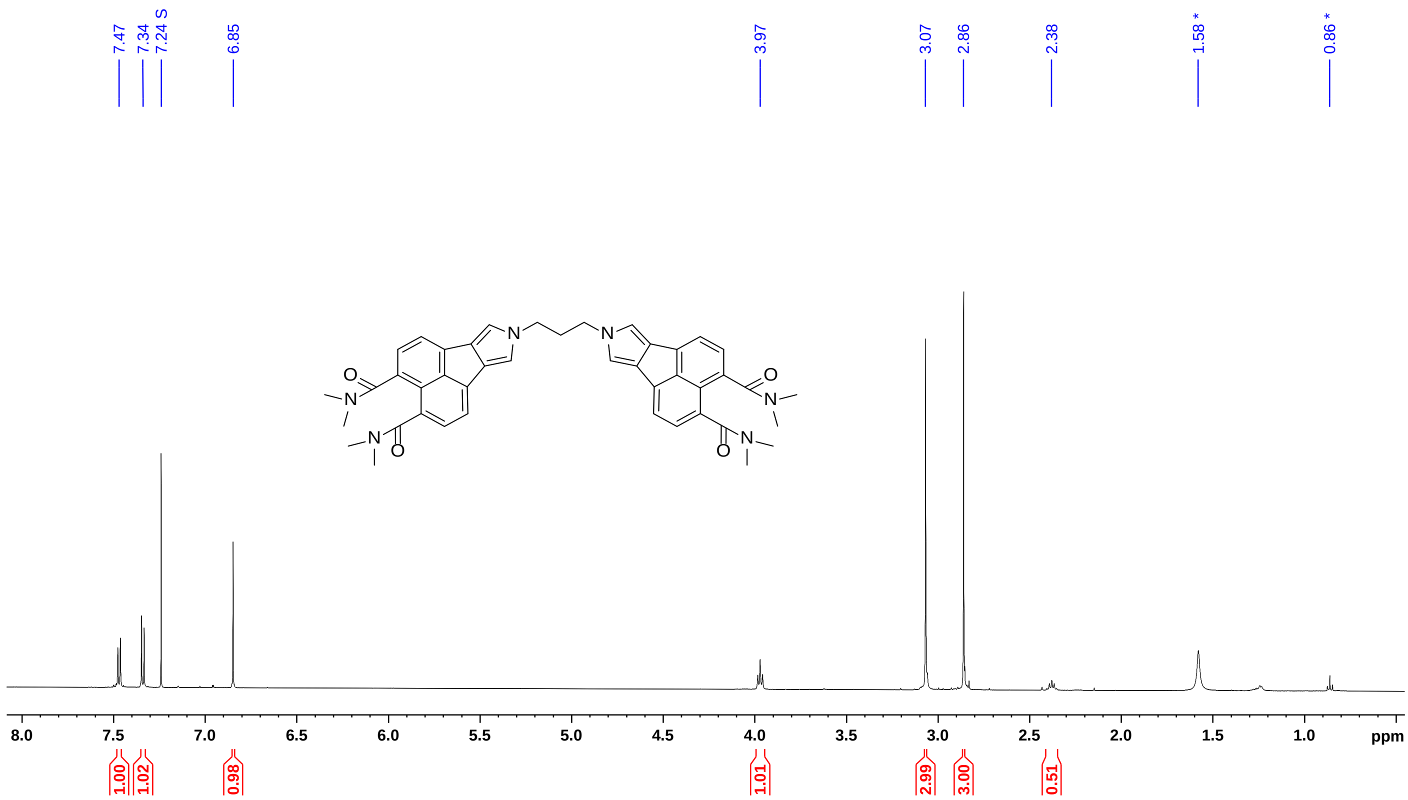

Figure S38. ^{1}H NMR spectrum of **NDA3**^{H} (600 MHz, chloroform-*d* , 300 K).

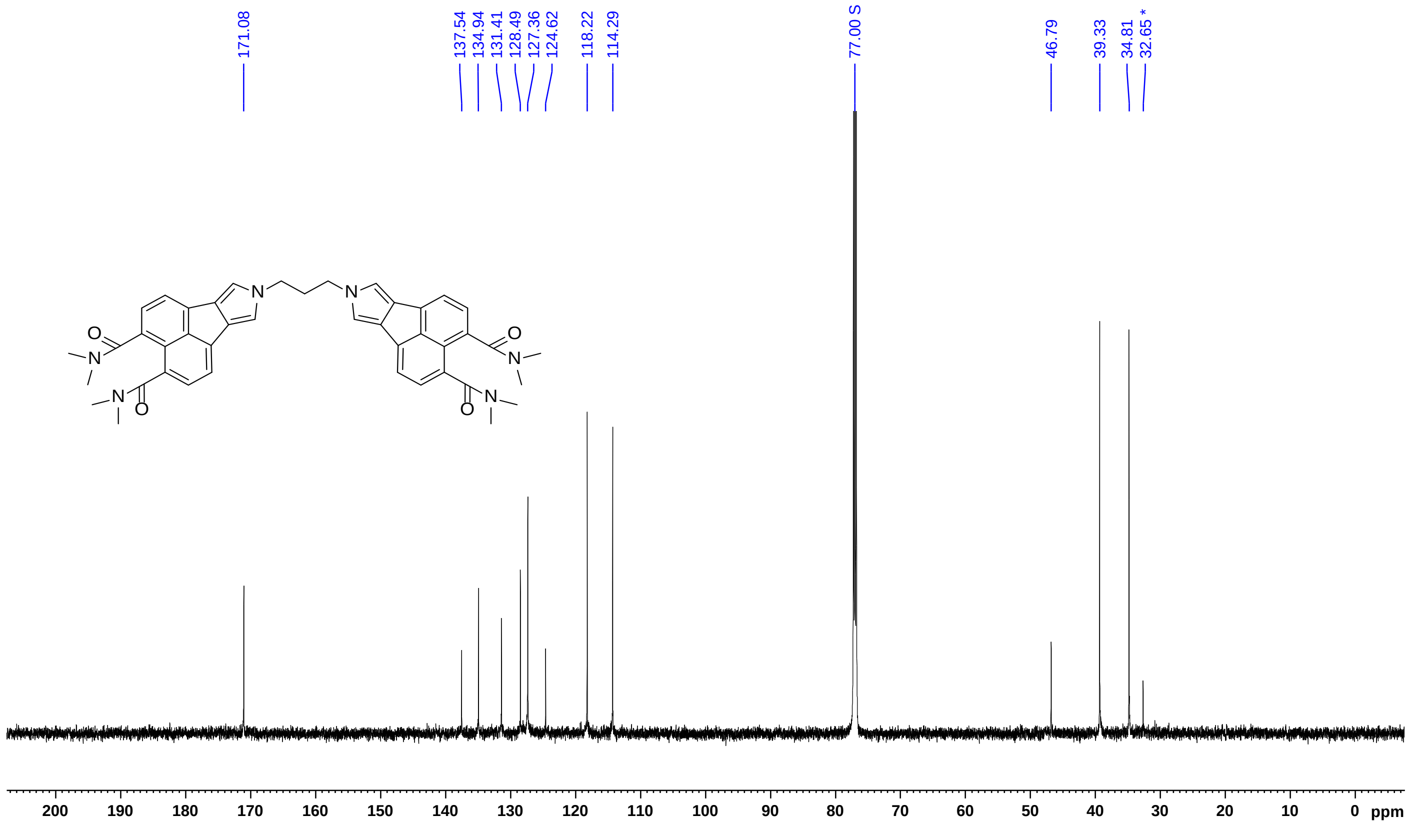

Figure S39. ^{13}C NMR spectrum of **NDA3**^{H} (600 MHz, chloroform-*d* , 300 K).

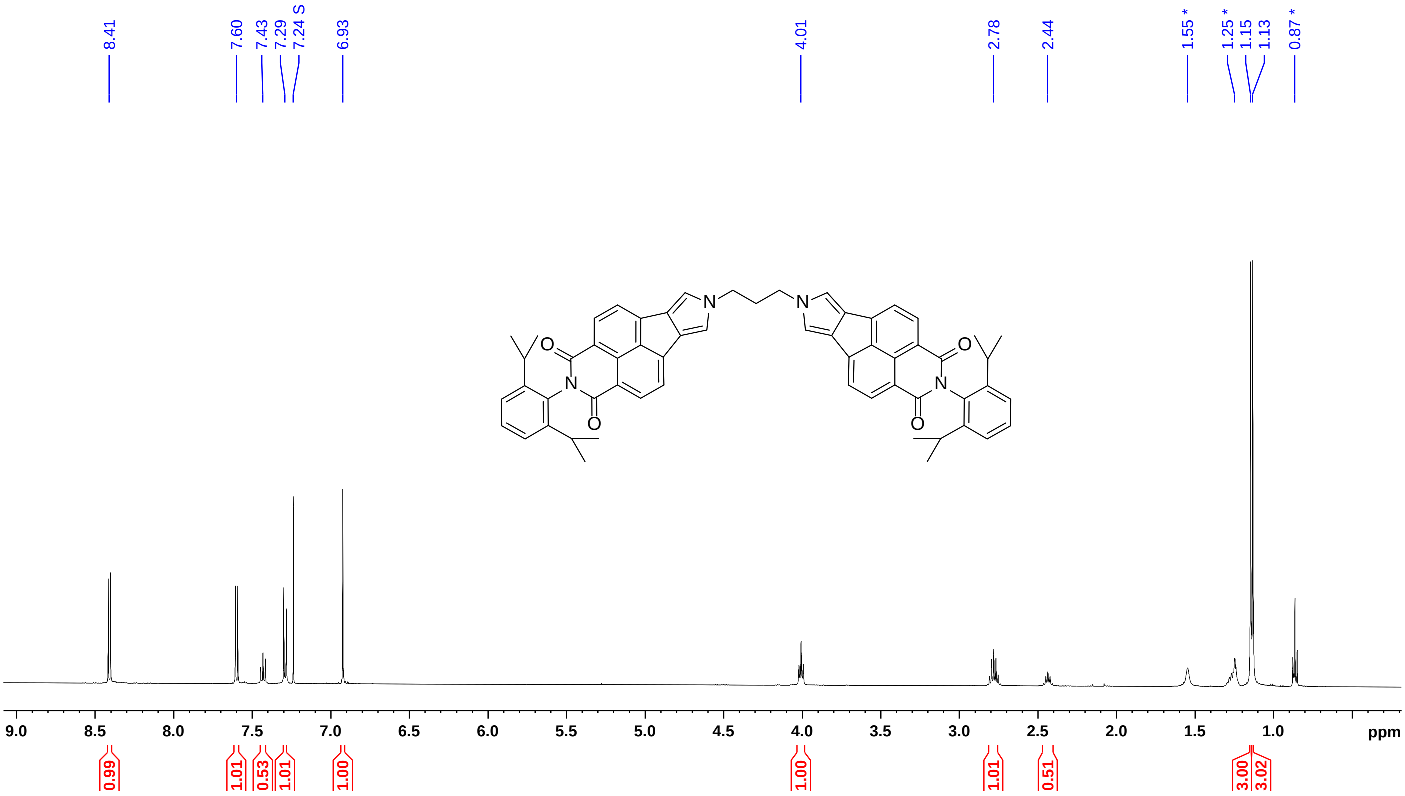

Figure S40. ^{1}H NMR spectrum of **NMI3**^{H} (600 MHz, chloroform-*d* , 300 K).

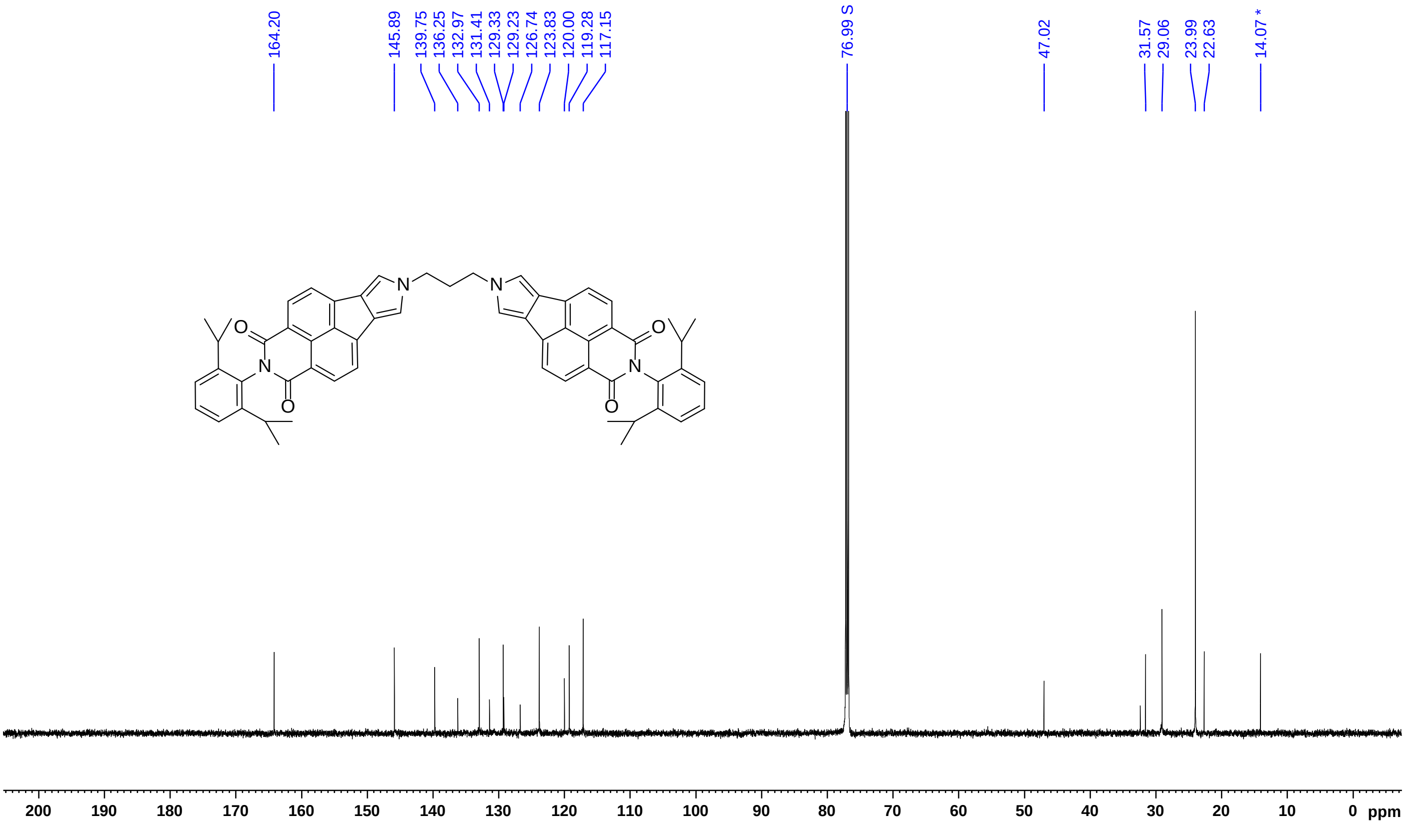

Figure S41. ^{13}C NMR spectrum of **NMI3**^{H} (151 MHz, chloroform-*d* , 300 K).

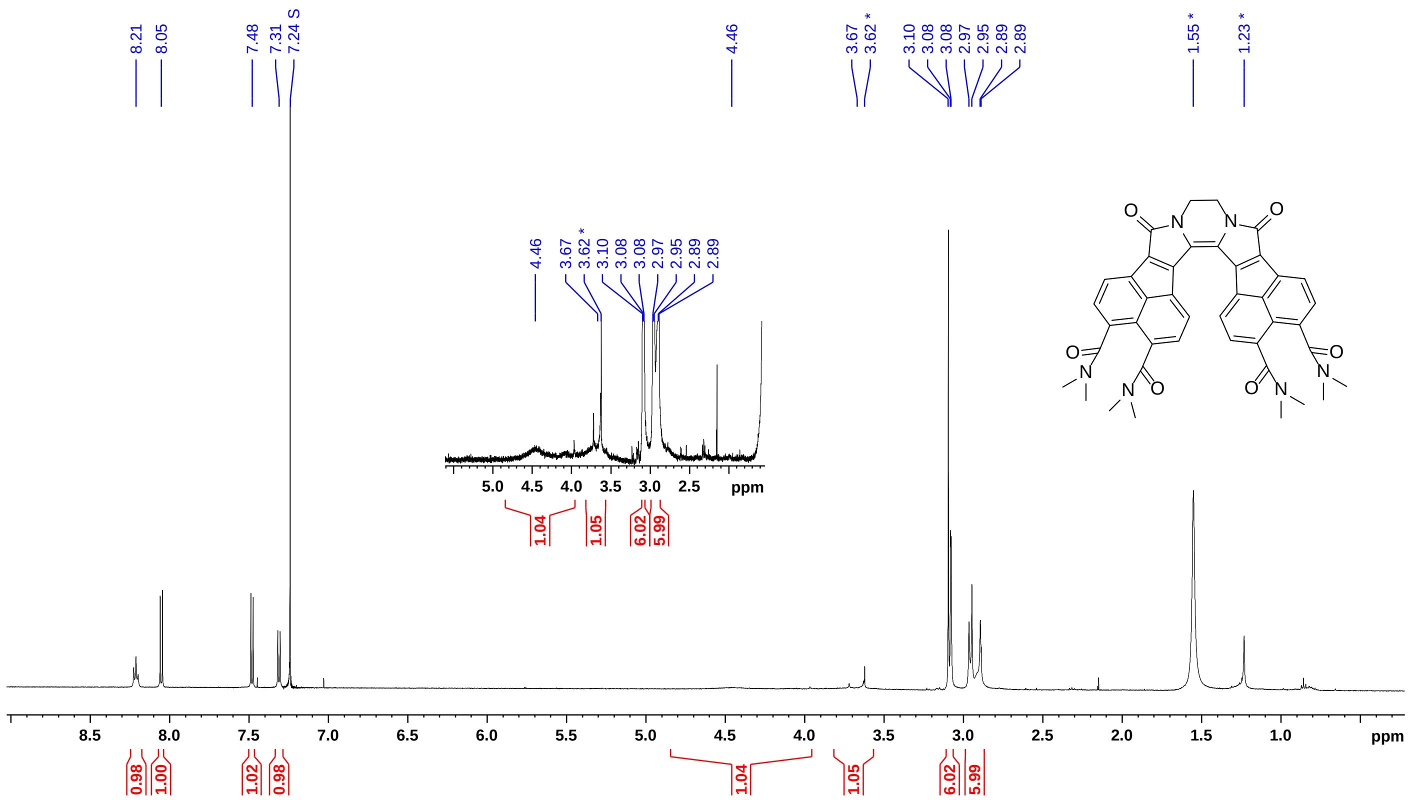

Figure S42. ^{1}H NMR spectrum of **cNDA2**^{O} (500 MHz, chloroform-*d* , 300 K).

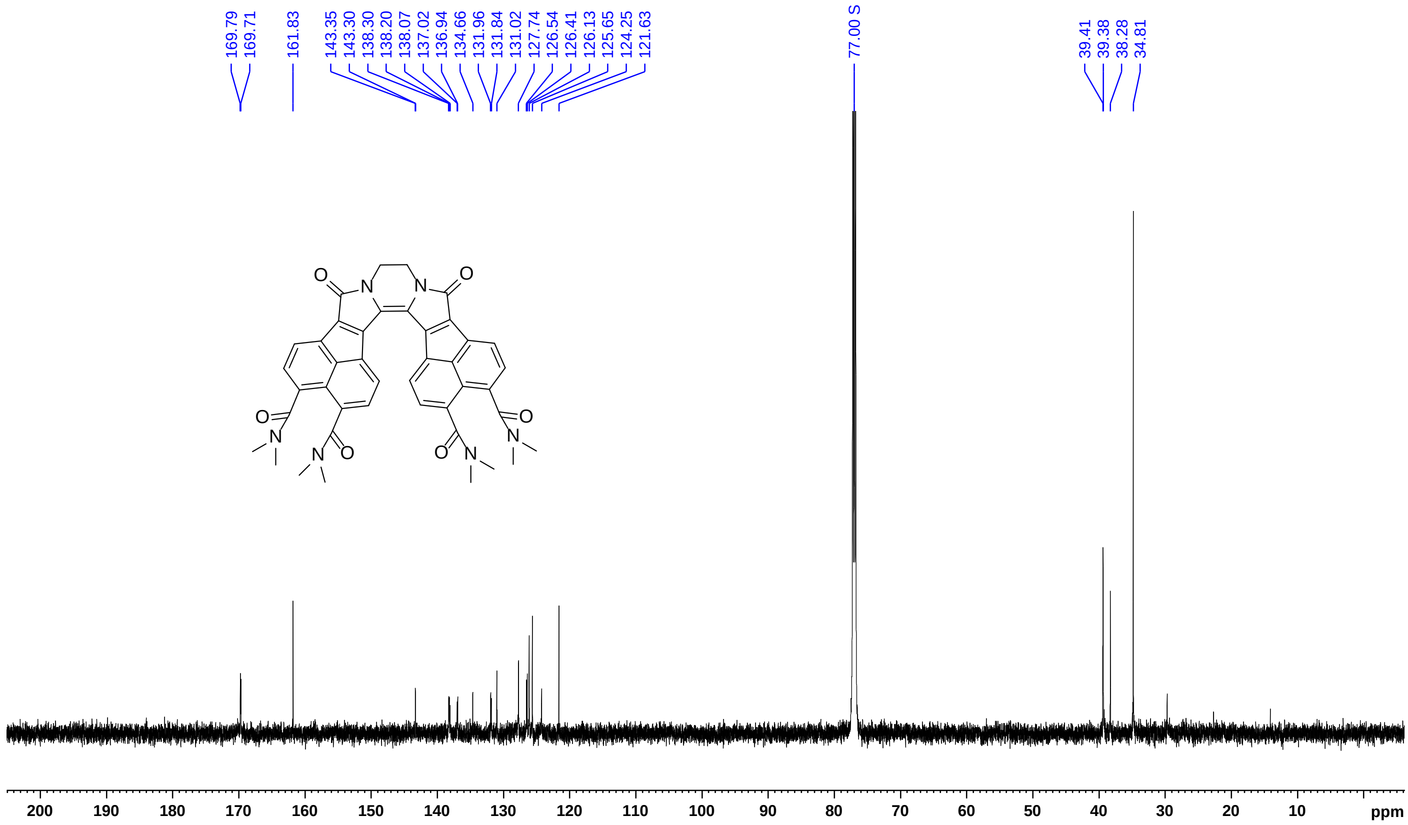

Figure S43. ^{13}C NMR spectrum of **cNDA2**^{O} (151 MHz, chloroform-*d* , 300 K).

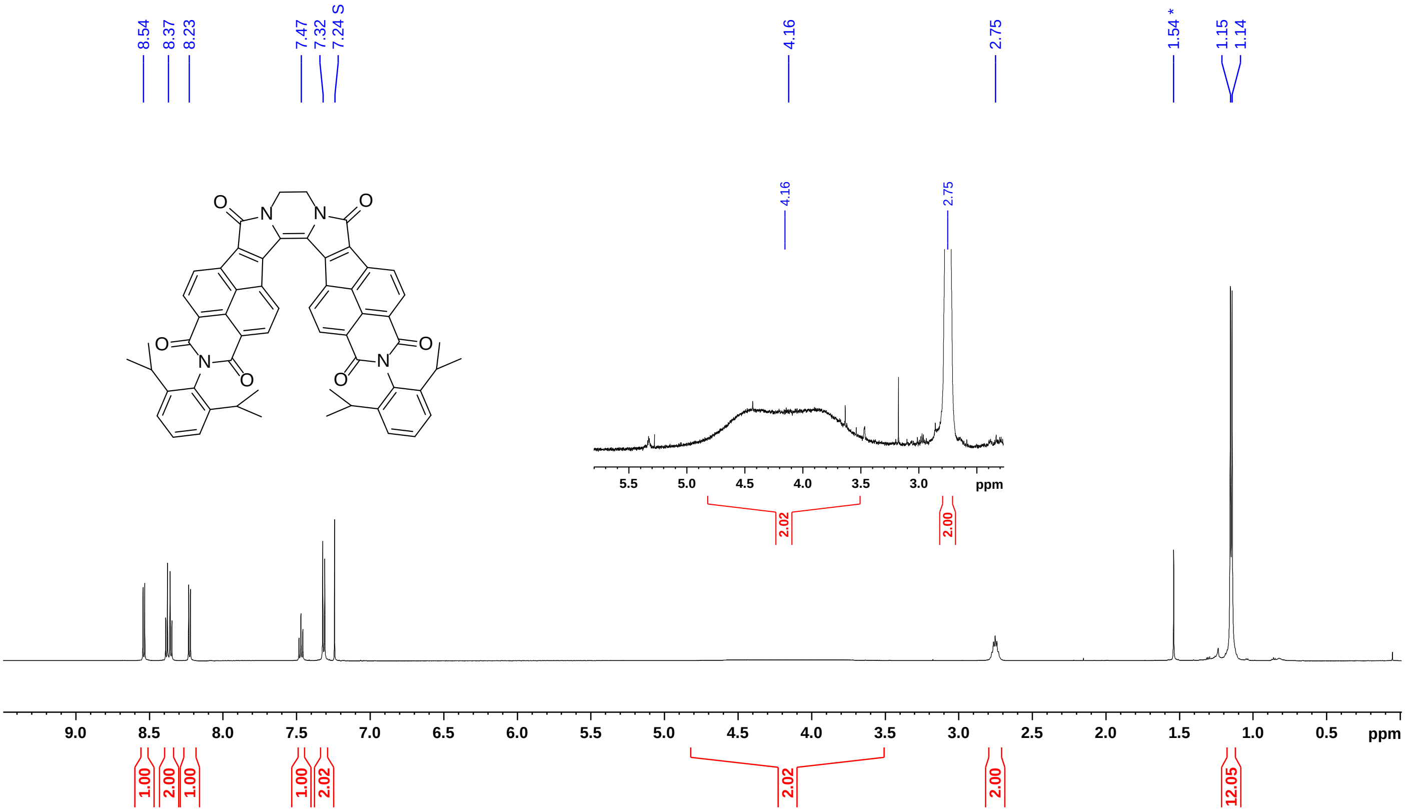

Figure S44. ^{1}H NMR spectrum of **cNMI2**^{O} (600 MHz, chloroform-*d* , 300 K).

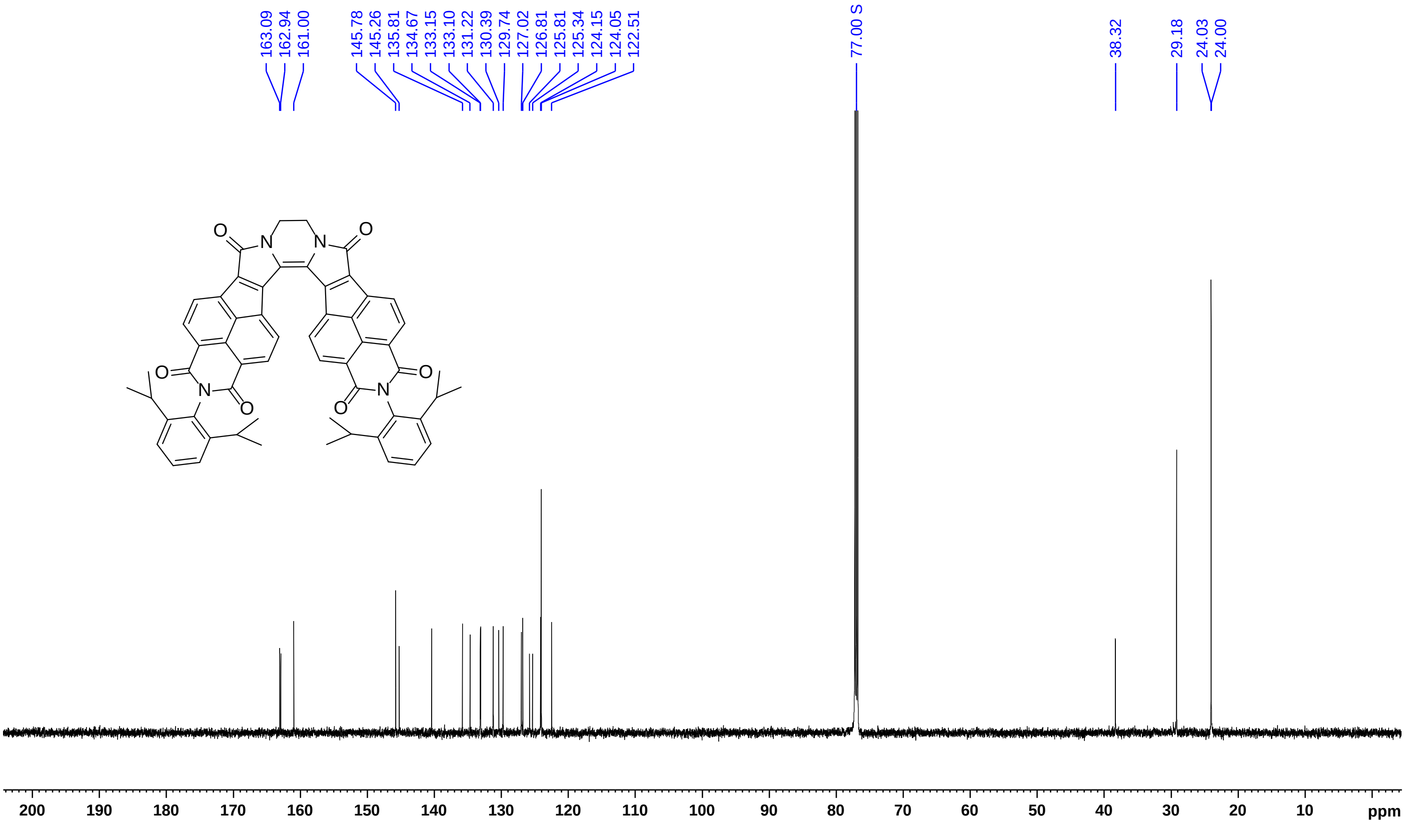

Figure S45. ^{13}C NMR spectrum of **cNMI2**^{O} (151 MHz, chloroform-*d* , 300 K).

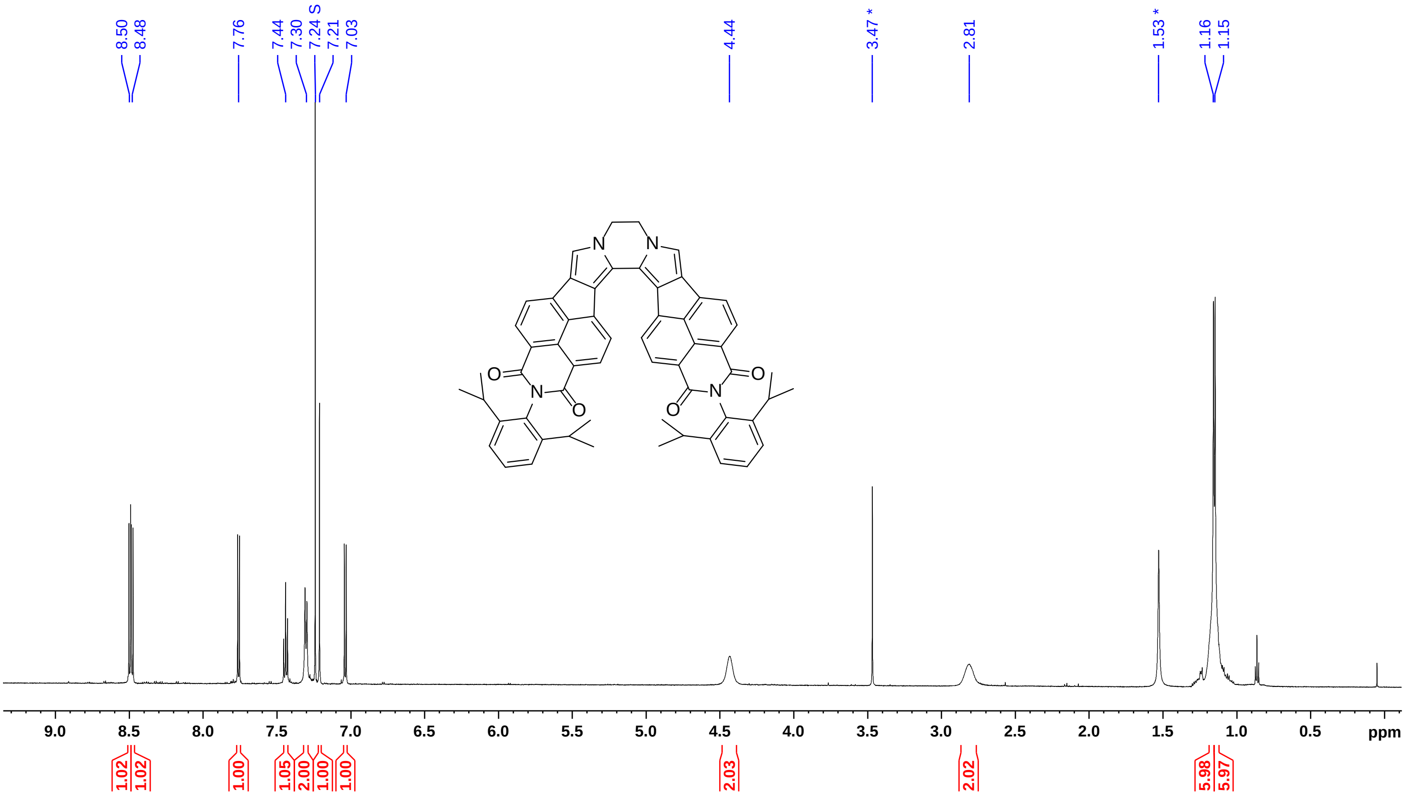

Figure S46. ^{1}H NMR spectrum of **cNMI2**^{H} (600 MHz, chloroform-*d* , 300 K).

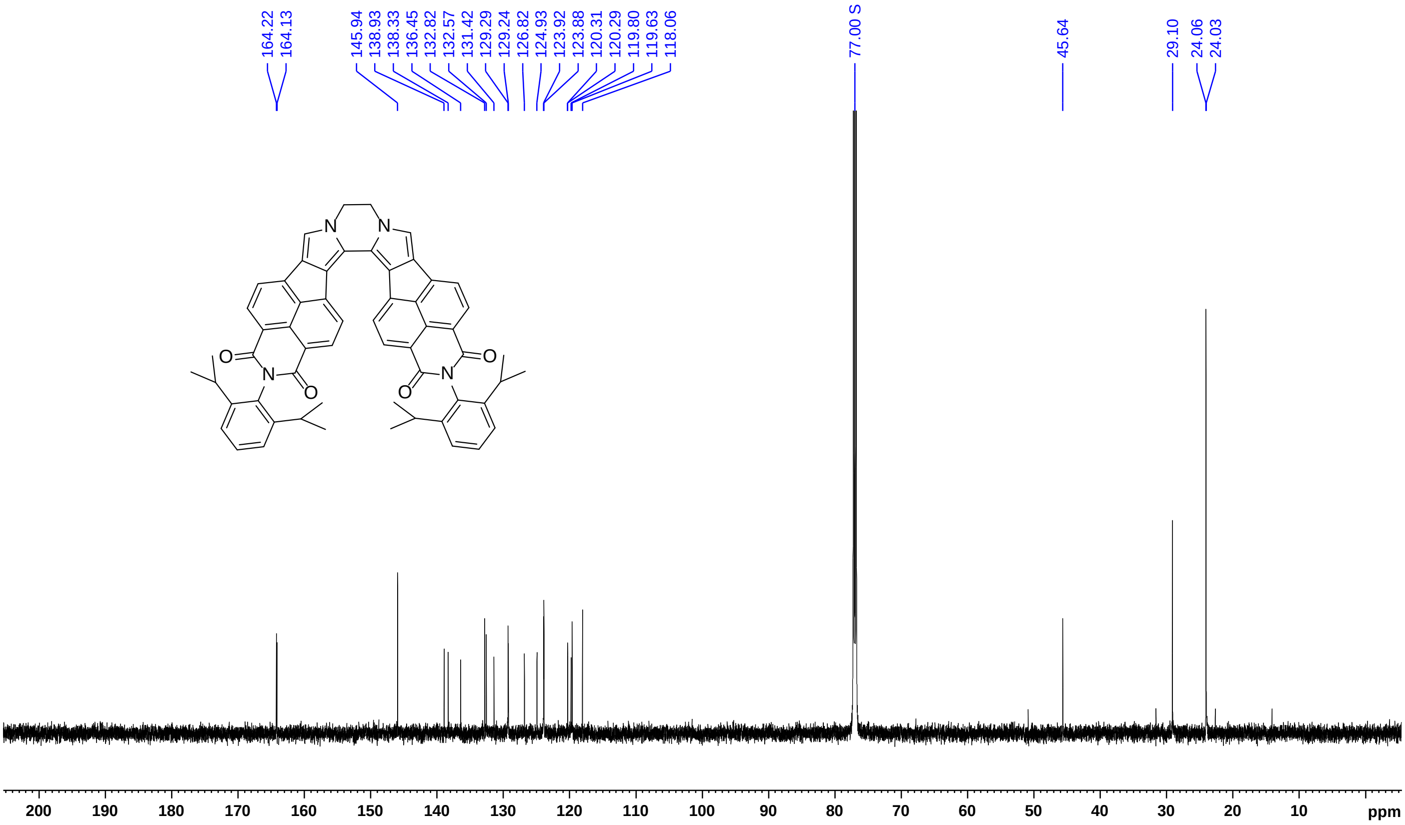

Figure S47. ^{13}C NMR spectrum of **cNMI2**^{H} (151 MHz, chloroform-*d* , 300 K).

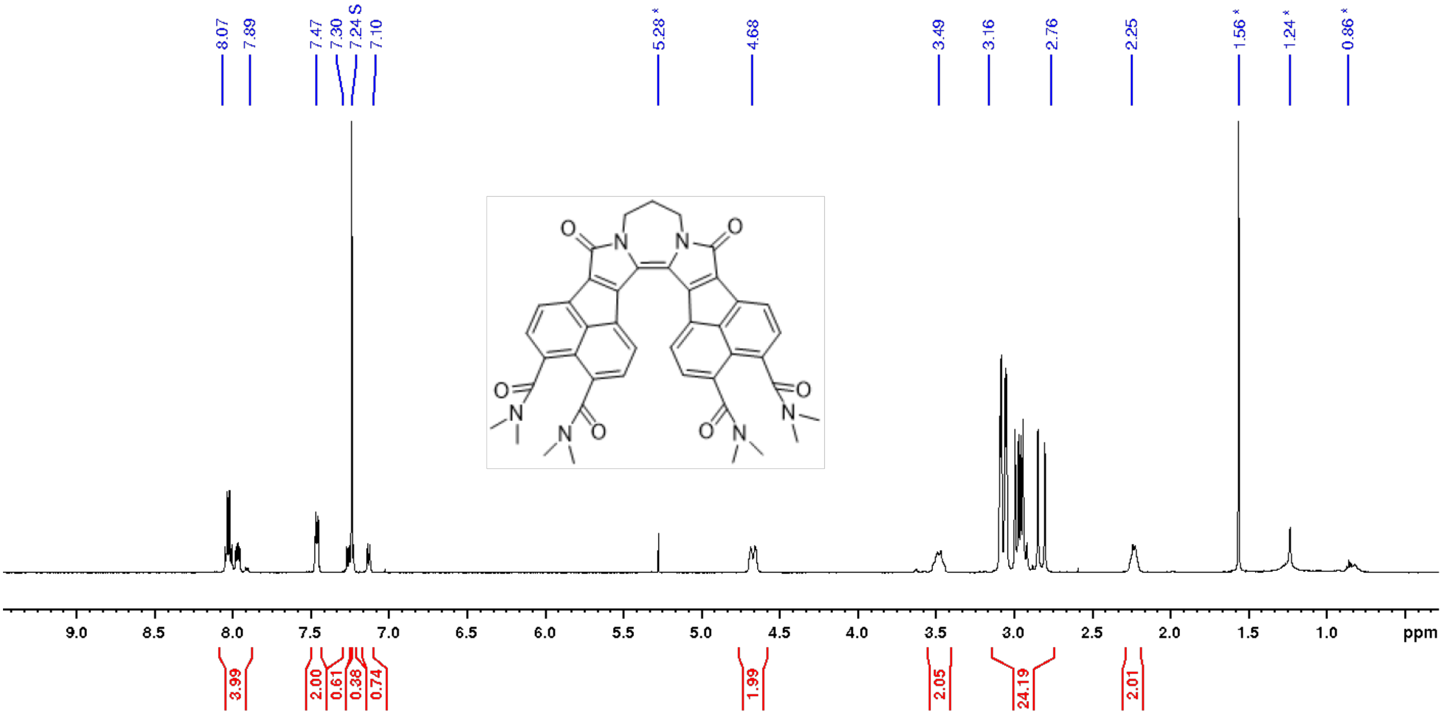

Figure S48. ^{1}H NMR spectrum of **cNDA3**^{O} (500 MHz, chloroform-$d_{\#}$, 300 K).

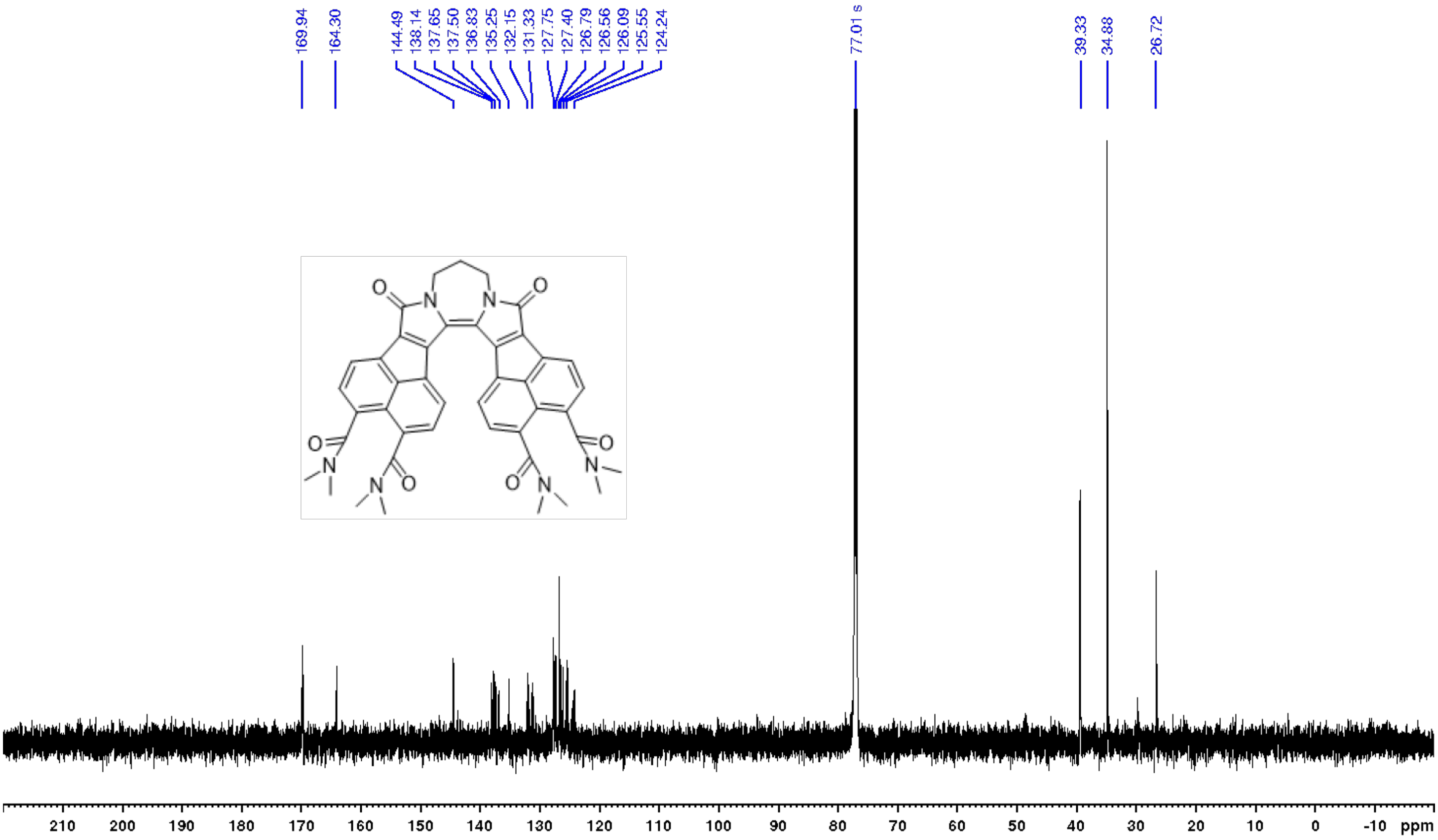

Figure S49. ^{13}C NMR spectrum of **cNDA3**^{O} (151 MHz, chloroform-$d_{\#}$, 300 K).

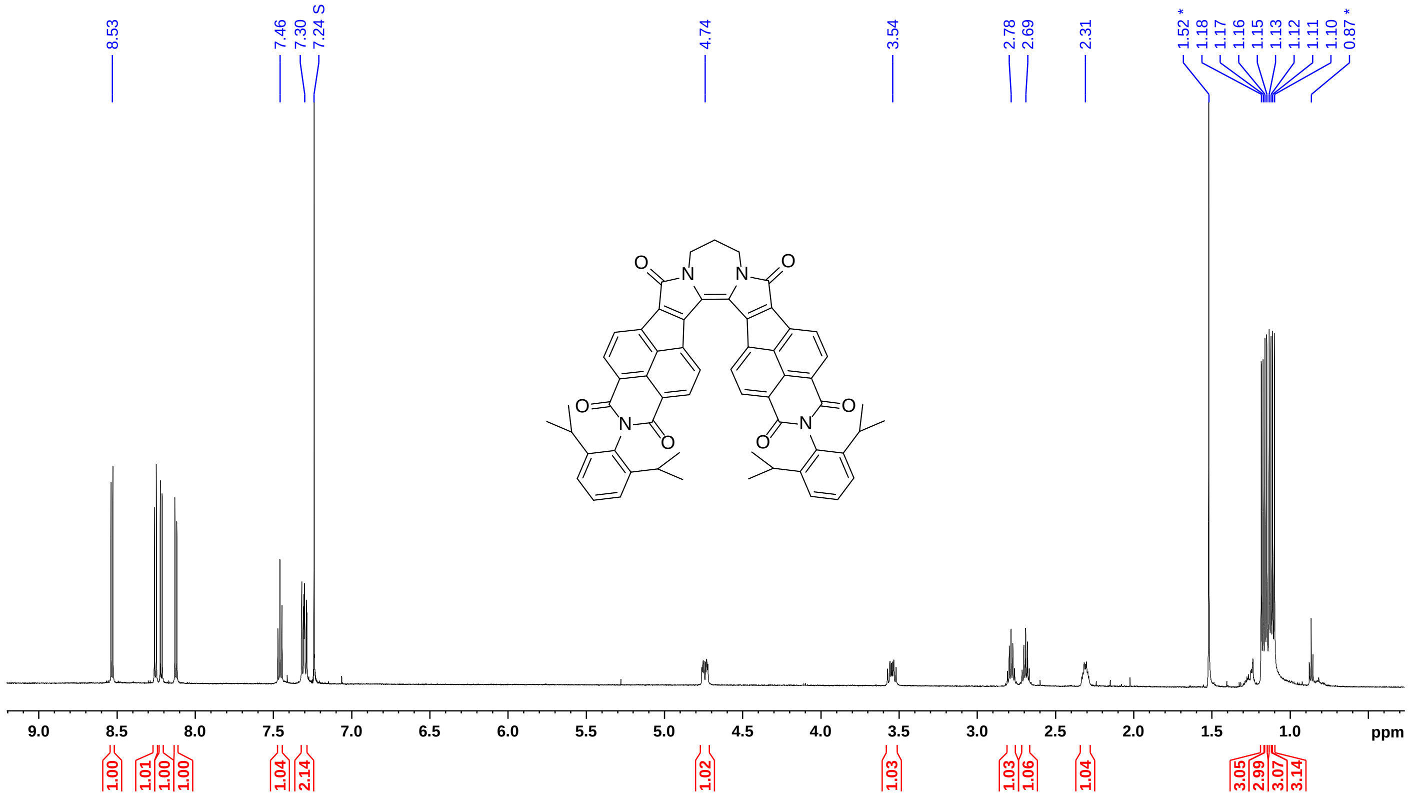

Figure S50. ^{1}H NMR spectrum of **cNMI3**^{O} (600 MHz, chloroform-*d* , 300 K).

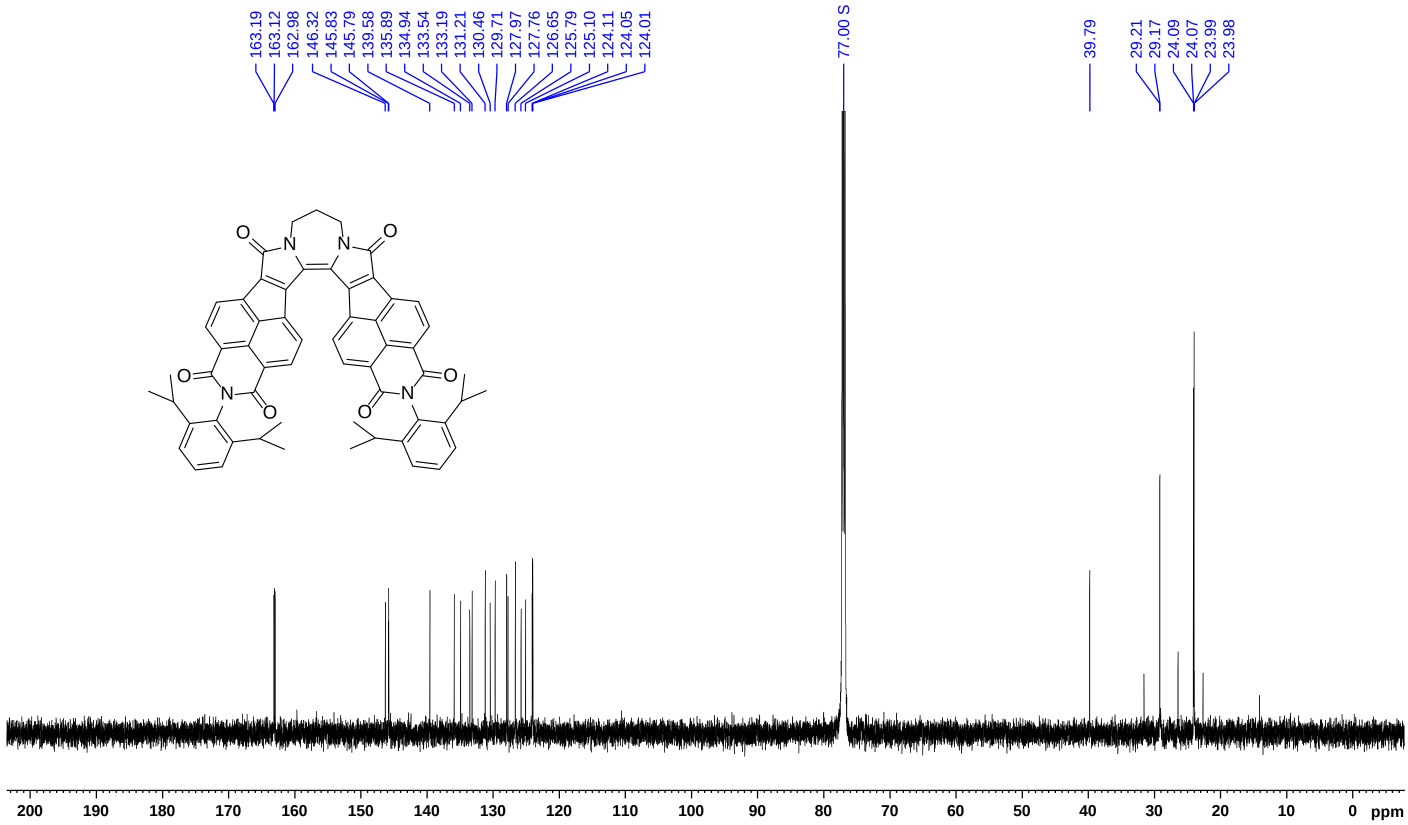

Figure S51. ^{13}C NMR spectrum of **cNMI**3° (600 MHz, chloroform-*d* , 300 K).

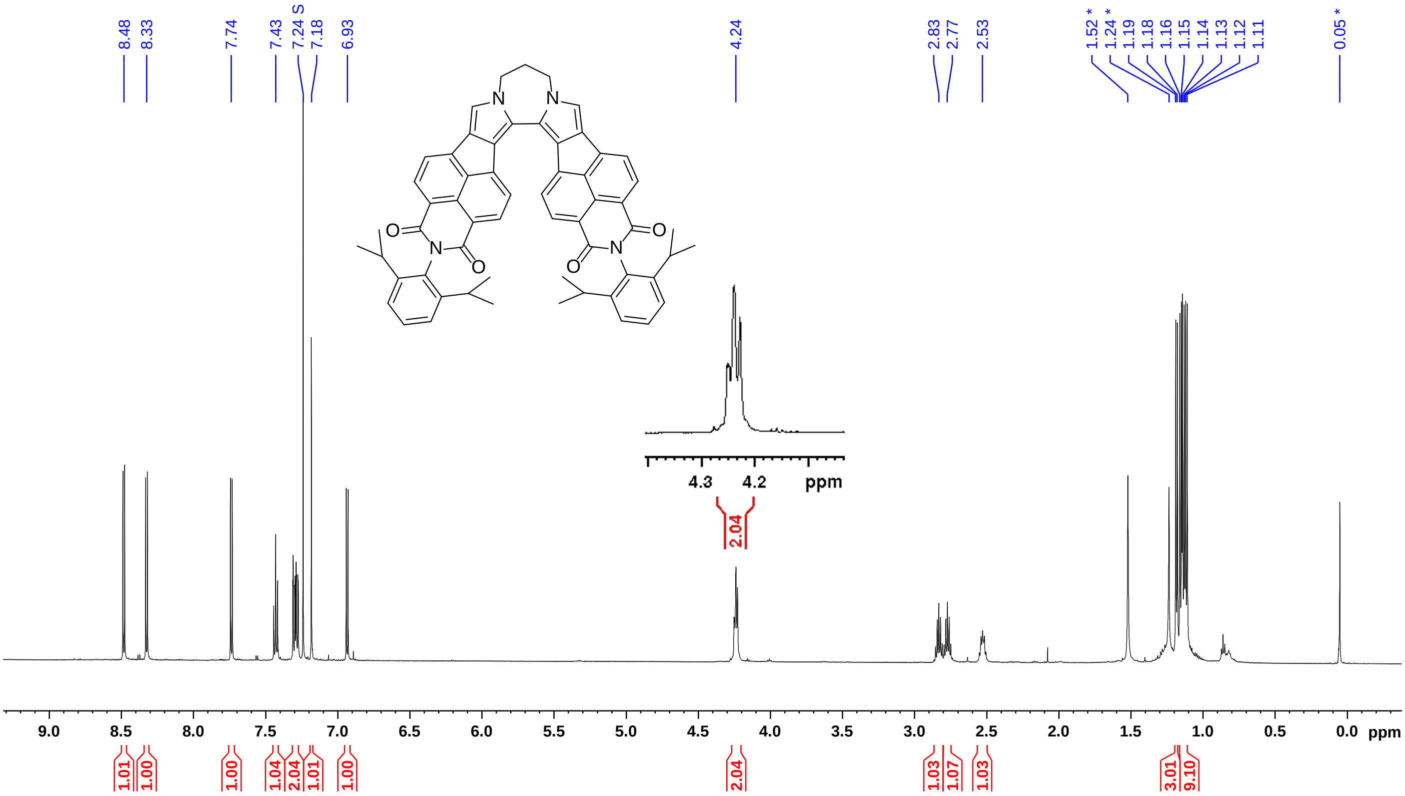

Figure S52. ^{1}H NMR spectrum of **cNMI3**^{H} (600 MHz, chloroform-*d* , 300 K) The diatereotopic protons of the bridge are nearly isochronous (4.24 ppm) and are observed as one multiplet with a higher-order splitting pattern.

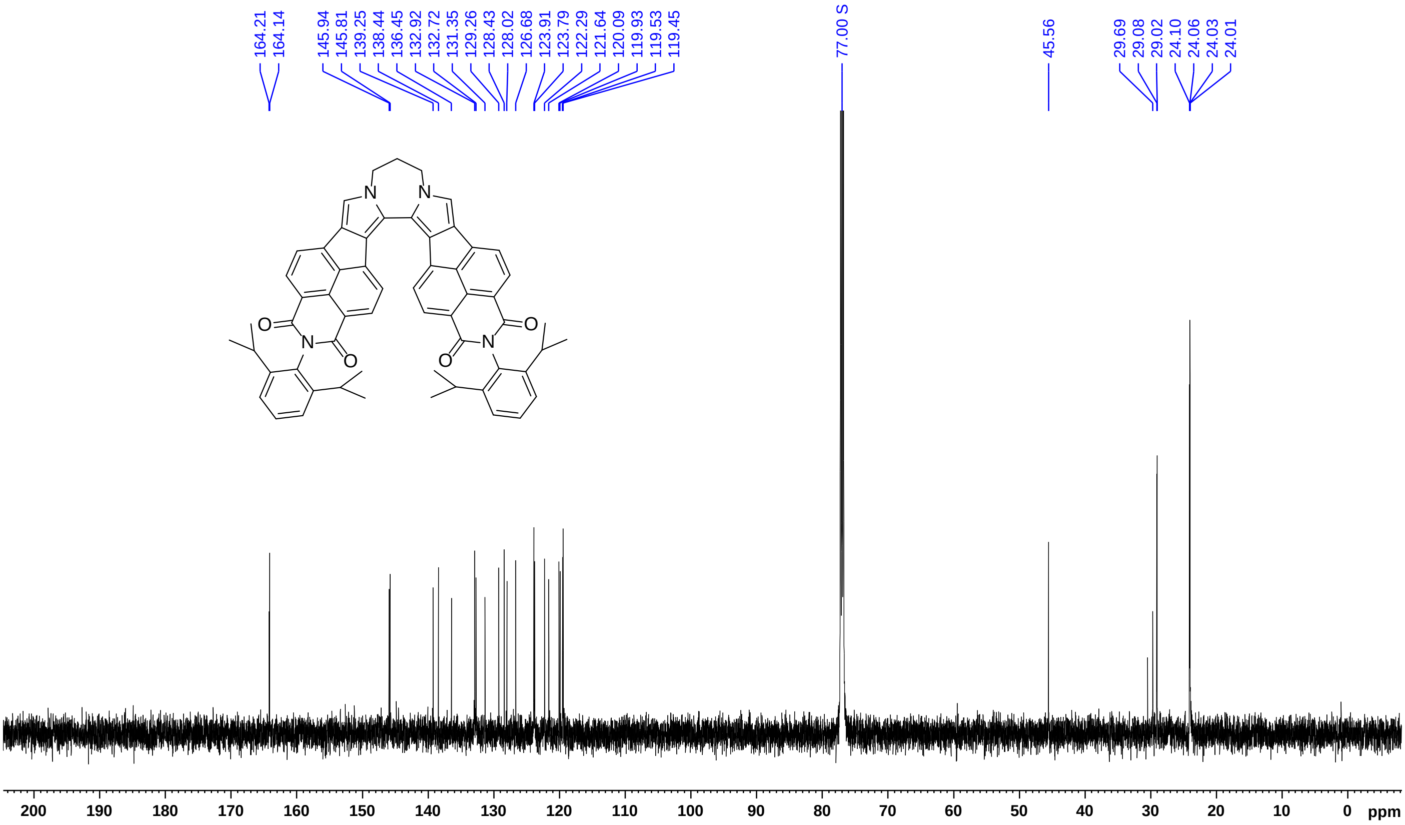

Figure S53. ^{13}C NMR spectrum of **cNMI3**^{H} (151 MHz, chloroform-*d* , 300 K).

Mass spectra

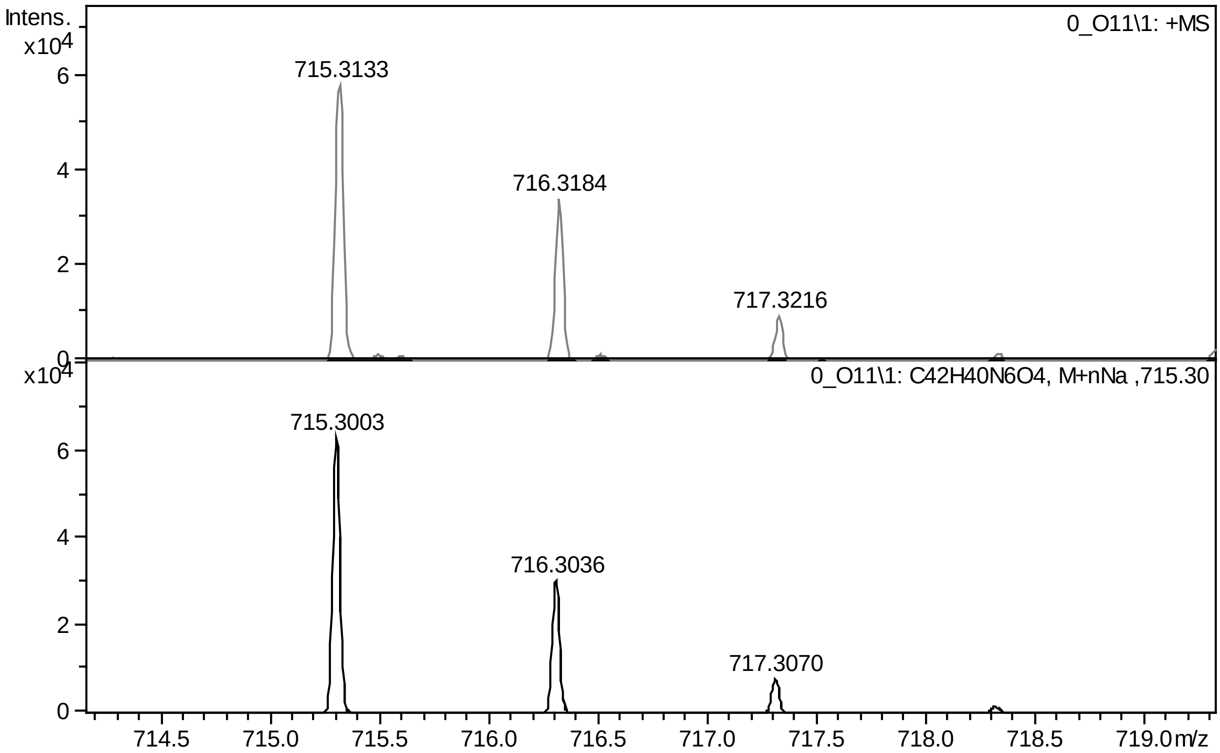

Figure S54. High resolution mass spectrum of **NDA2^{H}** (MALDI–TOF, top: experimental, bottom: simulated).

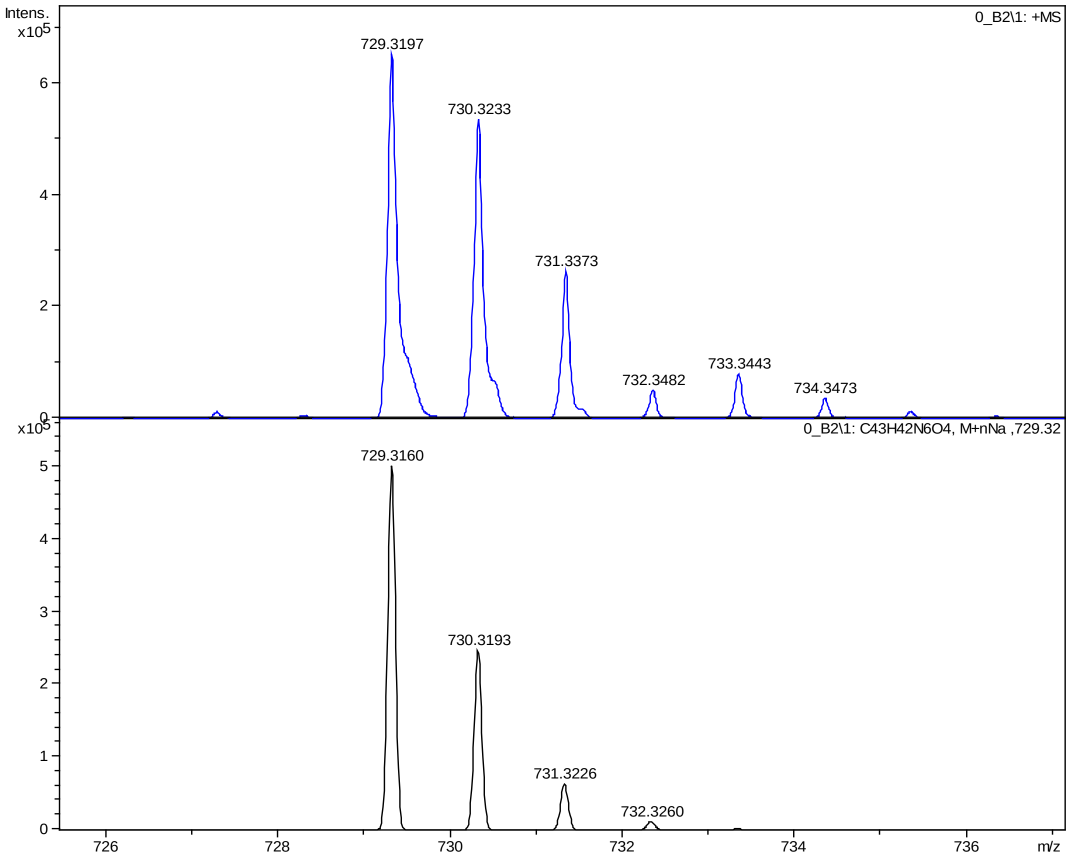

Figure S55. High resolution mass spectrum of **NDA3^{H}** (MALDI–TOF, top: experimental, bottom: simulated).

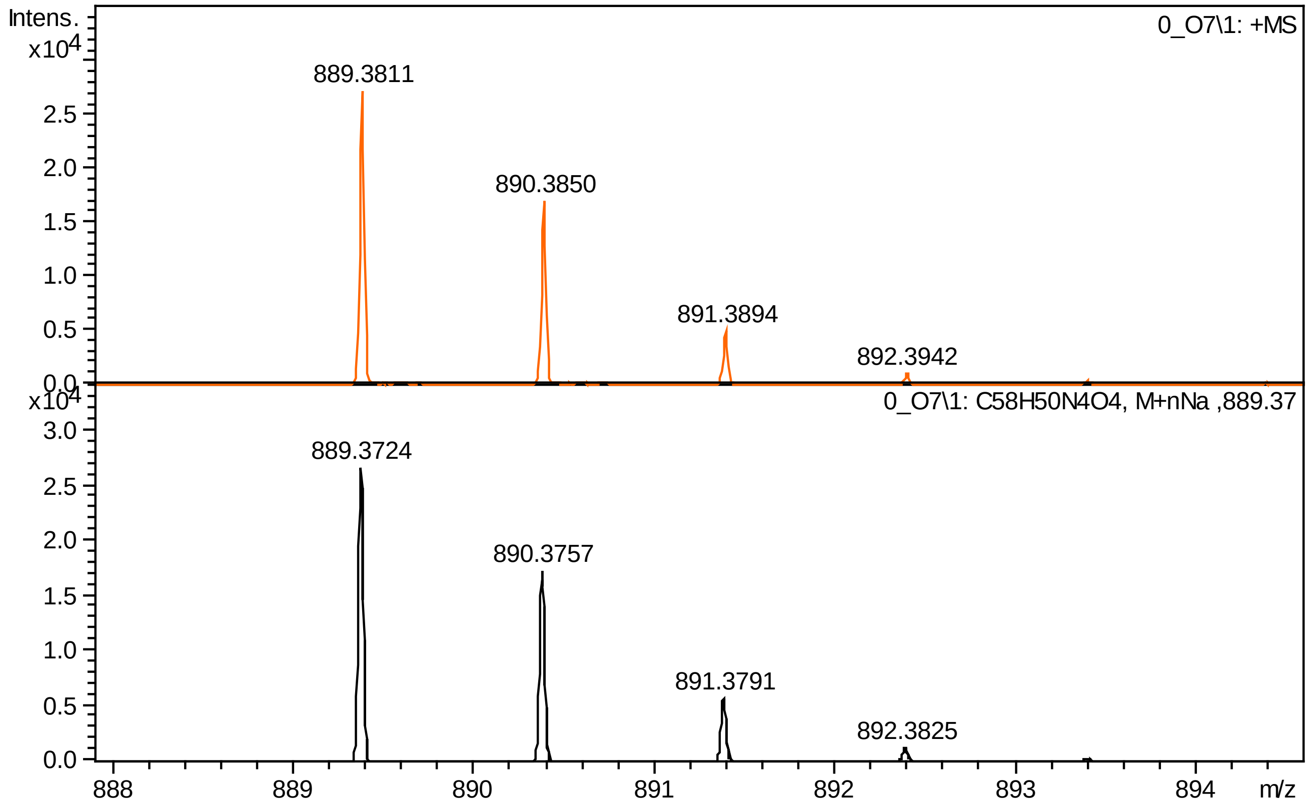

Figure S56. High resolution mass spectrum of **NMI2^{H}** (MALDI–TOF, top: experimental, bottom: simulated).

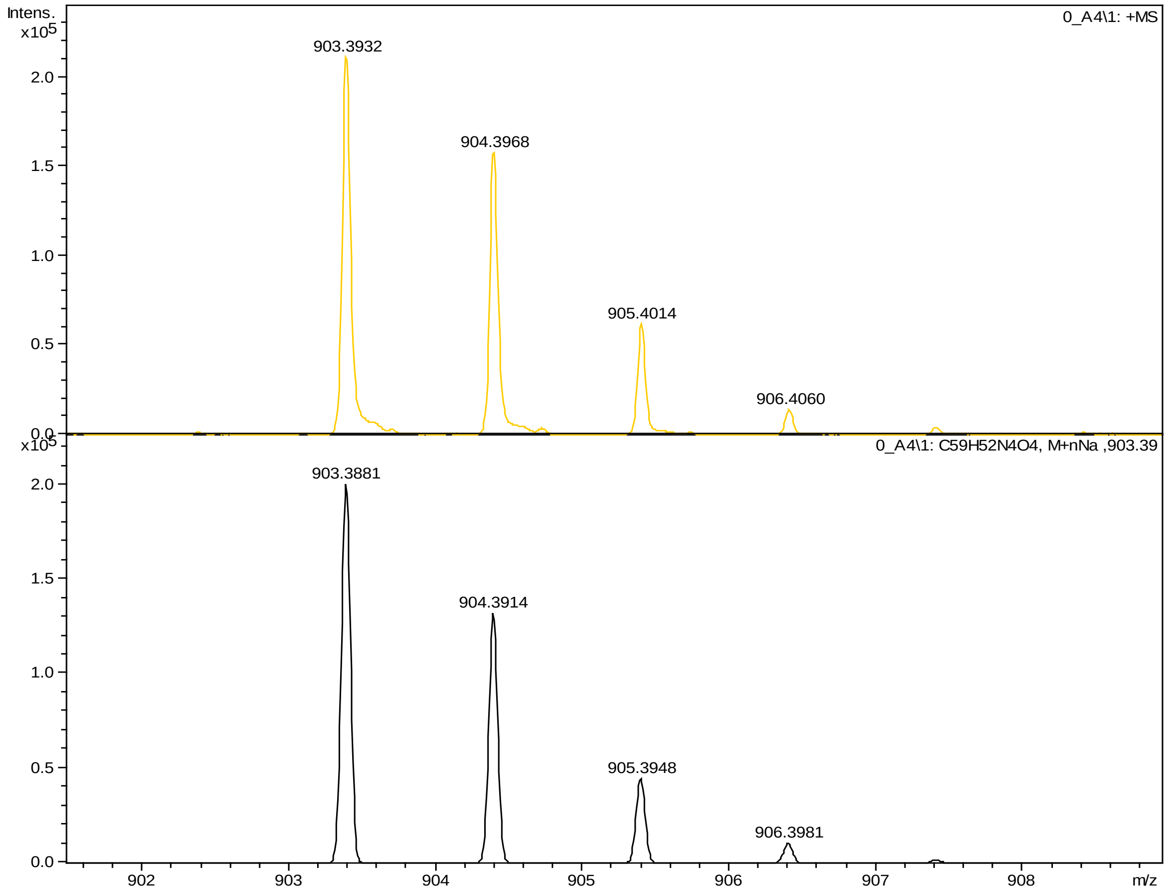

Figure S57. High resolution mass spectrum of **NMI3^{H}** (MALDI–TOF, top: experimental, bottom: simulated).

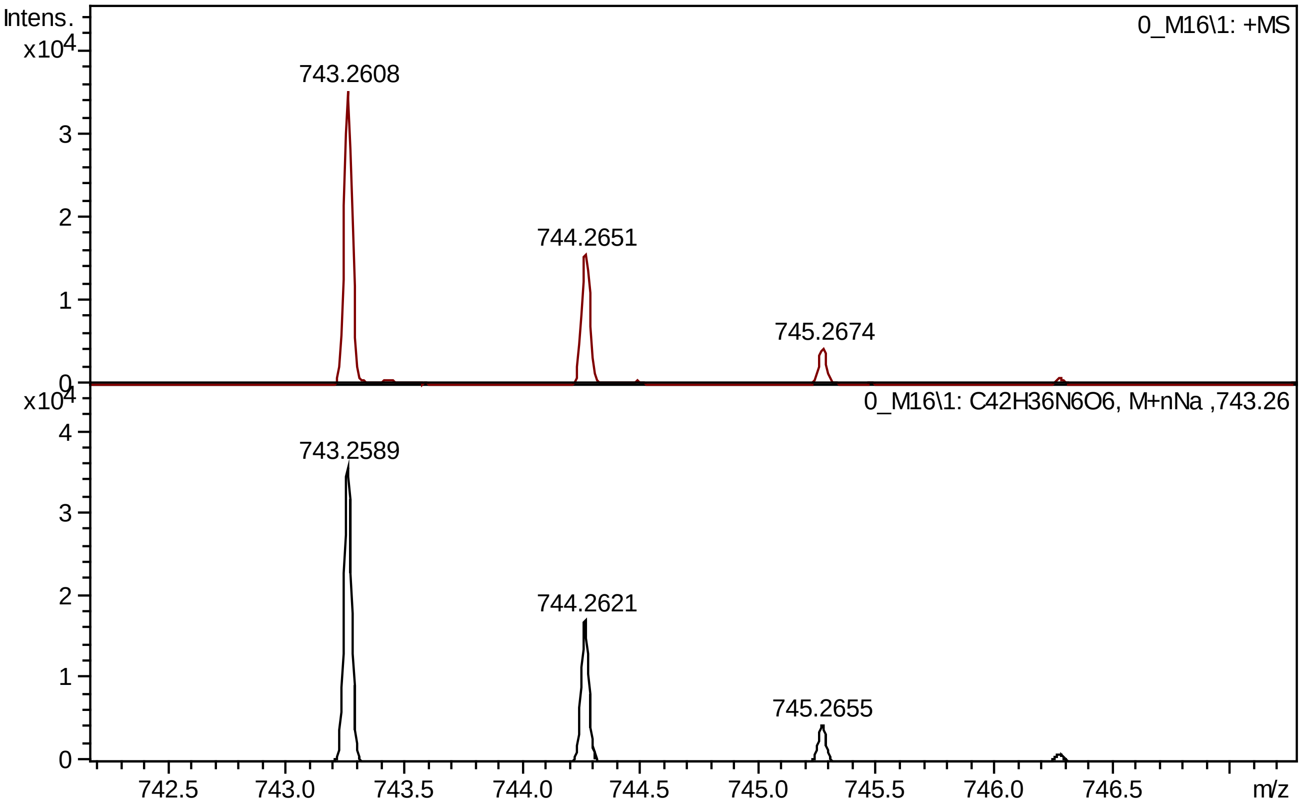

Figure S58. High resolution mass spectrum of **cNDA2°** (MALDI–TOF, top: experimental, bottom: simulated).

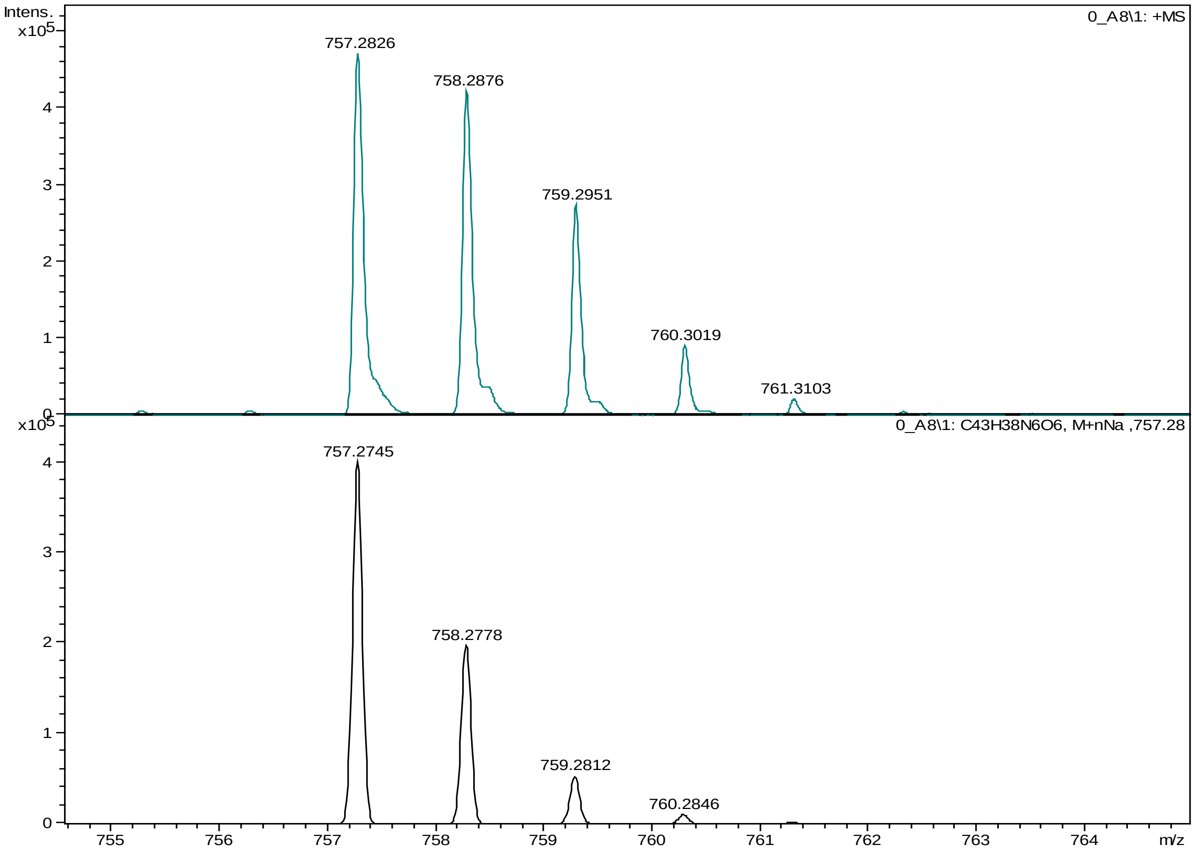

Figure S59. High resolution mass spectrum of **cNDA3°** (MALDI–TOF, top: experimental, bottom: simulated).

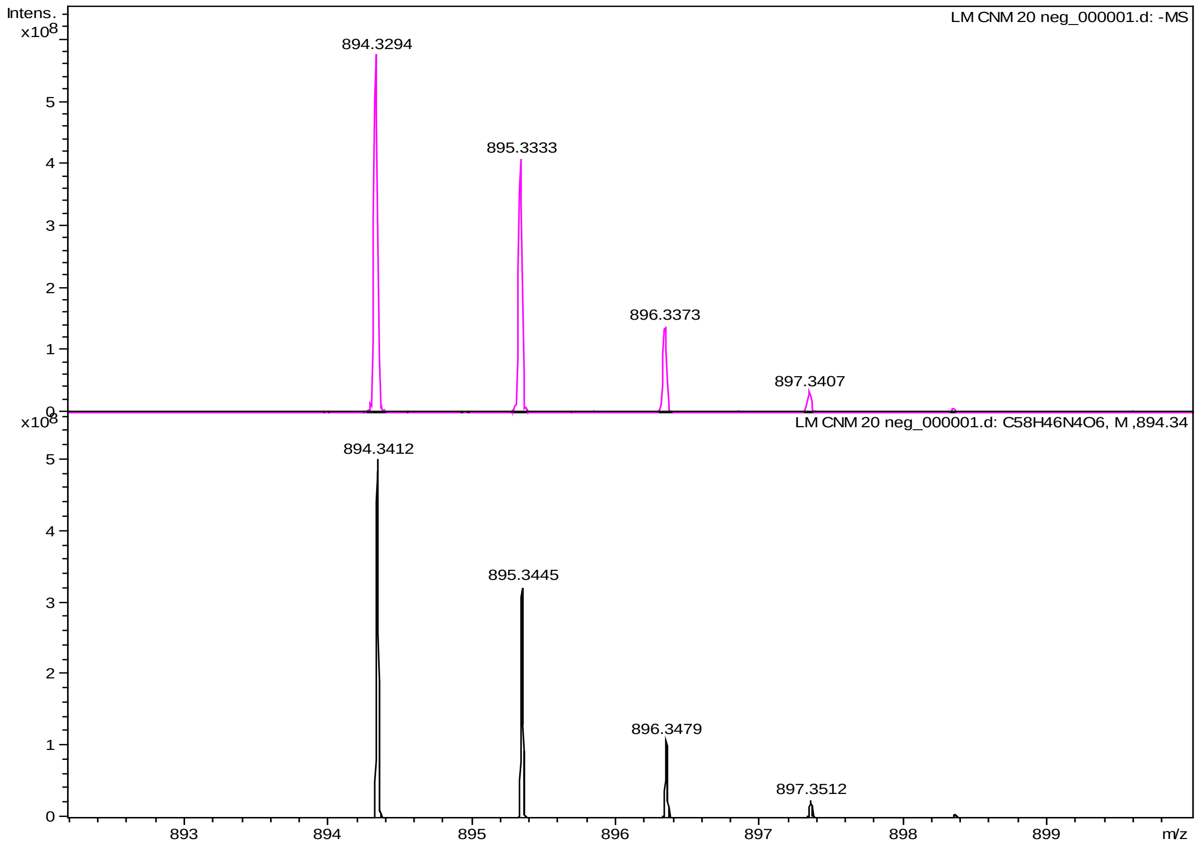

Figure S60. High resolution mass spectrum of **cNMI2^O** (MALDI–TOF, top: experimental, bottom: simulated).

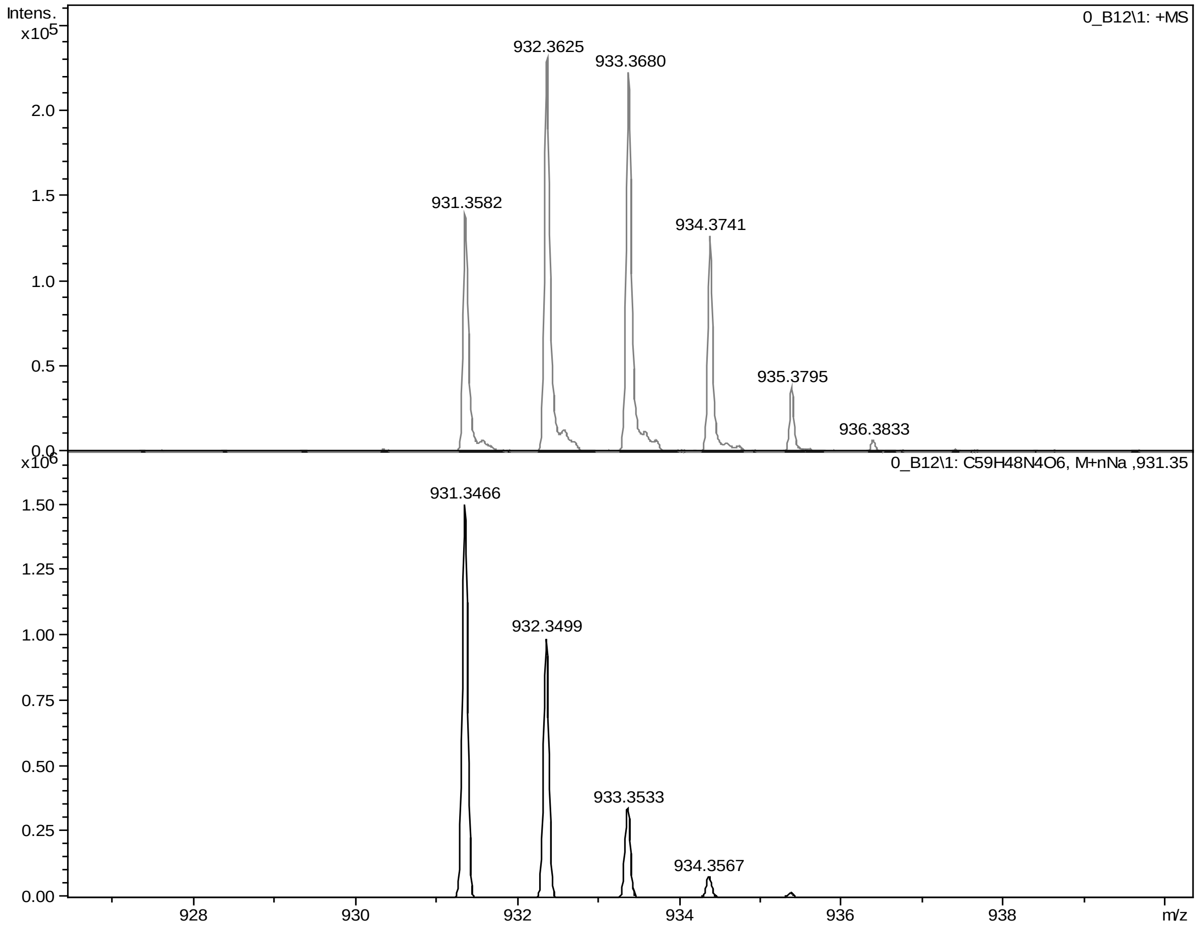

Figure S61. High resolution mass spectrum of **cNMI3^{O}** (MALDI–TOF, top: experimental, bottom: simulated).

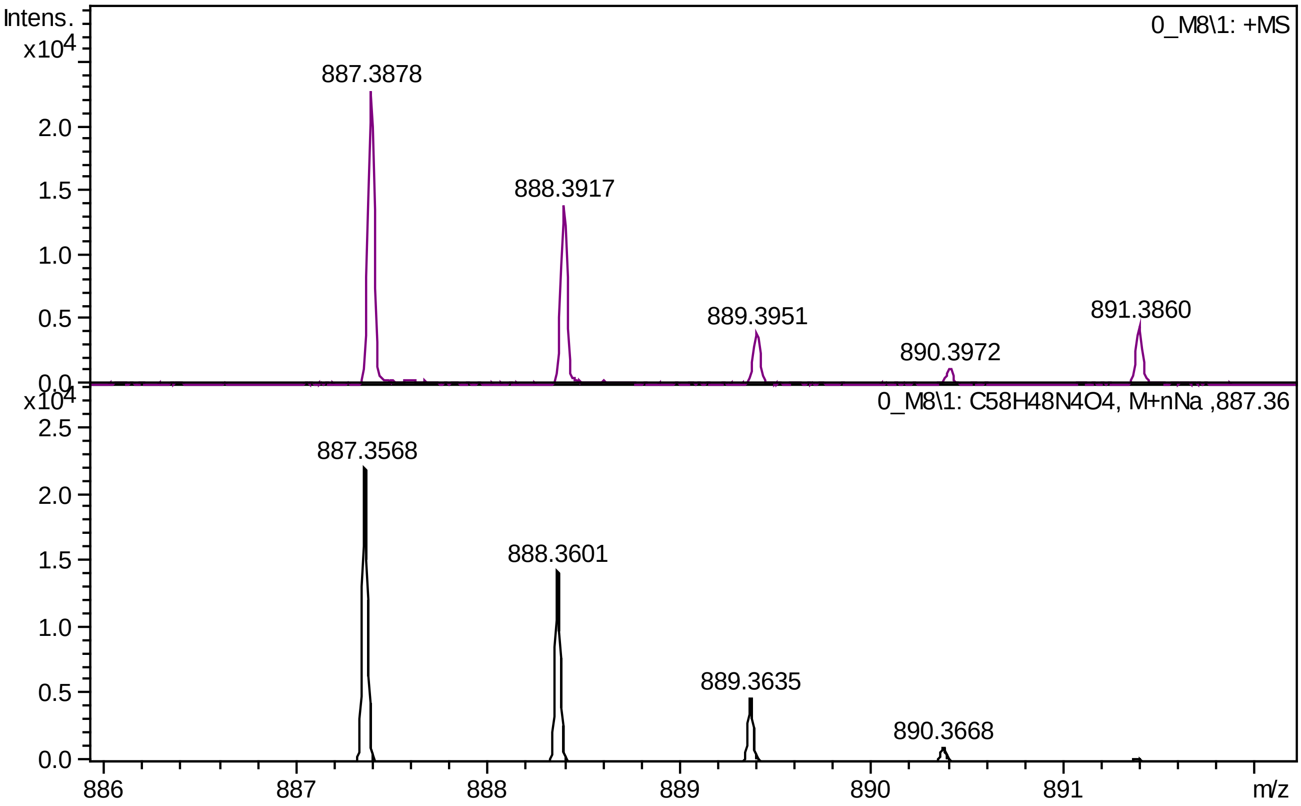

Figure S62. High resolution mass spectrum of **cNMI2^{H}** (MALDI–TOF, top: experimental, bottom: simulated).

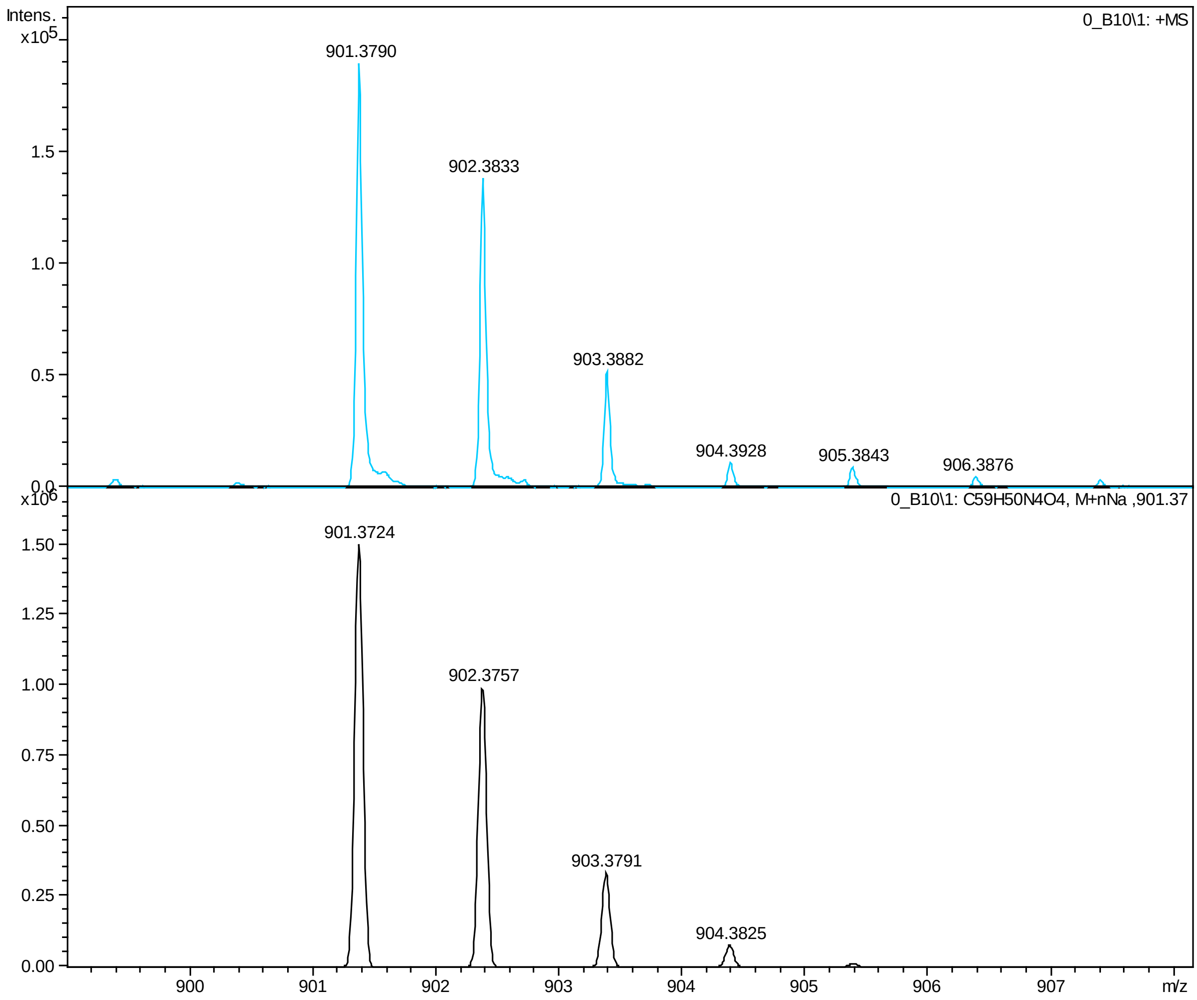

Figure S63. High resolution mass spectrum of **cNMI3^H** (MALDI–TOF, top: experimental, bottom: simulated).

References

(1) Zhylitskaya, H.; Cybińska, J.; Chmielewski, P.; Lis, T.; Stępień, M. Bandgap Engineering in π-Extended Pyrroles. a Modular Approach to Electron-Deficient Chromophores with Multi-Redox Activity. *J. Am. Chem. Soc.* **2016**, *138* (35), 11390–11398. https://doi.org/10.1021/jacs.6b07826.

(2) Żyła-Karwowska, M.; Moshniaha, L.; Hong, Y.; Zhylitskaya, H.; Cybińska, J.; Chmielewski, P. J.; Lis, T.; Kim, D.; Stępień, M. Electron-Deficient Bipyrrole Boomerangs: Bright Fluorophores Obtained via Double C–H Bond Activation. *Chem. – Eur. J.* **2018**, *24* (29), 7525–7530. https://doi.org/10.1002/chem.201801199.

(3) Frisch, M. J.; Trucks, G. W.; Schlegel, H. B.; Scuseria, G. E.; Robb, M. A.; Cheeseman, J. R.; Scalmani, G.; Barone, V.; Petersson, G. A.; Nakatsuji, H.; Li, X.; Caricato, M.; Izmaylov, A. F.; Zheng, G.; Sonnenberg, J. L.; Hada, M.; Ehara, M.; Toyota, K.; Fukuda, R.; Hasegawa, J.; Ishida, M.; Nakajima, T.; Honda, Y.; Kitao, O.; Nakai, H.; Vreven, T.; Montgomery, Jr., J. A.; Peralta, J. E.; Ogliaro, F.; Bearpark, M.; Heyd, J. J.; Brothers, E.; Kudin, K. N.; Staroverov, V. N.; Kobayashi, R.; Normand, J.; Raghavachari, K.; Rendell, A.; Burant, J. C.; Iyengar, S. S.; Tomasi, J.; Cossi, M.; Millam, J. M.; Klene, M.; Adamo, C.; Gomperts, R.; Stratmann, R. E.; Yazyev, O.; Austin, A. J.; Cammi, R.; Pomelli, C.; Ochterski, J. W.; Martin, R. L.; Morokuma, K.; Zakrzewski, V. G.; Voth, G. A.; Salvador, P.; Dannenberg, J. J.; Dapprich, S.; Daniels, A. D.; Farkas, O.; Foresman, J. B.; Fox, D. J. *Gaussian 16, Revision B.01*; Wallingford CT, 2016.

(4) Becke, A. D. Density-Functional Exchange-Energy Approximation with Correct Asymptotic Behavior. *Phys. Rev. A* **1988**, *38* (6), 3098–3100.

(5) Becke, A. D. Density-functional Thermochemistry. III. The Role of Exact Exchange. *J. Chem. Phys.* **1993**, *98* (7), 5648–5652. https://doi.org/10.1063/1.464913.

(6) Lee, C.; Yang, W.; Parr, R. G. Development of the Colle-Salvetti Correlation-Energy Formula into a Functional of the Electron Density. *Phys. Rev. B* **1988**, *37* (2), 785–789. https://doi.org/10.1103/PhysRevB.37.785.

(7) Chai, J.-D.; Head-Gordon, M. Long-Range Corrected Hybrid Density Functionals with Damped Atom-Atom Dispersion Corrections. *Phys. Chem. Chem. Phys. PCCP* **2008**, *10* (44), 6615–6620. https://doi.org/10.1039/b810189b.

(8) Hirata, S.; Head-Gordon, M. Time-Dependent Density Functional Theory within the Tamm–Dancoff Approximation. *Chem. Phys. Lett.* **1999**, *314* (3–4), 291–299. https://doi.org/10.1016/S0009-2614(99)01149-5.
